# Biospeciation of Oxidovanadium(IV) Imidazolyl–Carboxylate Complexes and Their Action on Glucose-Stimulated Insulin Secretion in Pancreatic Cells

**DOI:** 10.3390/molecules29030724

**Published:** 2024-02-04

**Authors:** Vital Ugirinema, Frank Odei-Addo, Carminita L. Frost, Zenixole R. Tshentu

**Affiliations:** 1 Department of Chemistry, Nelson Mandela University, P.O. Box 77000, Port Elizabeth 6031, South Africa; 2 Department of Chemistry, College of Science and Technology, University of Rwanda, Kigali P.O. Box 3900, Rwanda; 3 Department of Biochemistry and Microbiology, Nelson Mandela University, P.O. Box 77000, Port Elizabeth 6031, South Africa; frank.odeiaddo@gmail.com

**Keywords:** vanadium, biospeciation, diabetes, glucose-stimulated insulin secretion

## Abstract

The reaction of the vanadyl ion (VO^2+^) with imidazole-4-carboxylic acid (Im4COOH), imidazole-2-carboxylic acid (Im2COOH) and methylimidazole-2-carboxylic acid (MeIm2COOH), respectively, in the presence of small bioligands (bL) [oxalate (Ox), lactate (Lact), citrate (Cit) and phosphate (Phos)] and high-molecular-weight (HMW) human serum proteins [albumin (HSA) and transferrin (hTf)] were studied in aqueous solution using potentiometric acid–base titrations. The species distribution diagrams for the high-molecular-mass (HMM) proteins with oxidovanadium(IV) under physiological pH were dominated by VO(HMM)_2_, VOL(HMM) for unsubstituted ligands (L^−^ = Im4COO^−^ and Im2COO^−^). However, for the N-substituted MeIm2COOH, the species distribution diagrams under physiological pH were dominated by VOL_2_, VO(HMM)_2_ and VO_2_L_2_(HMM). These species were further confirmed by LC-MS, MALDI-TOF-MS and EPR studies. The glucose-stimulated insulin secretion (GSIS) action of the complexes was investigated using INS-1E cells at a 1 µM concentration, which was established through cytotoxicity studies via the MTT assay. The neutral complexes, especially VO(MeIm2COO)_2_, showed promising results in the stimulation of insulin secretion than the cationic [VO(MeIm2CH_2_OH)_2_]^2+^ complex and the vanadium salt. Oxidovanadium(IV) complexes reduced insulin stimulation significantly under normoglycaemic levels but showed positive effects on insulin secretion under hyperglycaemic conditions (33.3 mM glucose media). The islets exposed to oxidovanadium(IV) complexes under hyperglycaemic conditions displayed a significant increase in the stimulatory index with 1.19, 1.75, 1.53, 1.85, 2.20 and 1.29 observed for the positive control (sulfonylurea:gliclazide), VOSO_4_, VO(Im4COO)_2_, VO(Im2COO)_2_, VO(MeIm2COO)_2_ and VO(MeIm2CH_2_OH)_2_^2+^, respectively. This observation showed a potential further effect of vanadium complexes towards type 2 diabetes and has been demonstrated for the first time in this study.

## 1. Introduction

Diabetes mellitus (DM) is a disorder of metabolic homeostasis characterised by an elevated glucose concentration in blood plasma due to β-cell dysfunction [1]. The β-cell dysfunction makes the storage and the release of insulin complicated, and the body struggles to maintain a normal blood glucose concentration. Type I diabetes mellitus (TIDM) results from autoimmune destruction of the insulin-producing β-cells in the islets of Langerhans in the pancreas, resulting in the production of little or no insulin and type II diabetes mellitus (TIIDM) is characterised by insulin resistance by cells [2]. The current treatment of diabetes type I involves injection of insulin and patients are supposed to inject or wear an insulin pump subcutaneously. Type II diabetes mellitus is treated by several types of synthetic oral drugs together with a controlled diet, weight control and physical activity. Different classes of diabetes drugs like sulfonylureas, biguanides, thiozolidinediones and meglitinides have certain side effects that are undesirable [3,4,5].

**Figure 1:**
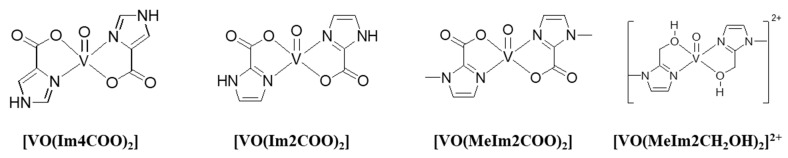
Chemical structures of the oxidovanadium(IV) complexes used in this study.

Oxidovanadium(IV) complexes have been extensively tested as anti-diabetic compounds, especially in +4 and +5 oxidation states [6], with maltolato and picolinato complexes such as BMOV and [VO(pic)_2_] emerging as lead complexes [7,8]. The insulin-enhancing effects of the oxovanadium(IV) picolinato complex have been clearly shown to be dependent on dose as well as delivery method. When [VO(pic)_2_] was administered orally to STZ-induced diabetic rats (0.2 mmol·kg^−1^ for 2 days followed by 0.1 mmol·kg^−1^ for 11 days), plasma glucose levels normalised while plasma insulin levels increased [1]. On the other hand, comparing [VO(pic)_2_] with BMOV [7,8], the picolinato complex had lower solubility and more gastrointestinal irritation for an equivalent dose, suggesting that there is room for further structural improvement in order to increase bioavailability and lessen side effects. In our own studies, we have introduced an imidazolyl–carboxylic acid moiety (Figure 1) that shows promise for the uptake of glucose, protein tyrosine phosphatase (PTP) inhibition and anticoagulative effects [9]. The other new systems introduced recently include the oxidovanadium(IV)–Schiff base system, which also shows potential as an insulin-mimetic compound, especially through PTP inhibition studies [10]. The imidazolyl–alcohol system was also introduced by us in this account in order to compare a cationic complex with neutral complexes. The glucose-stimulated insulin secretion (GSIS) action of the vanadium complexes is another effect that we set out to investigate [11]. GSIS is controlled by glucose-derived signals in pancreatic β-cells [12]. During hyperglycaemic conditions, insulin enhances glucose uptake and utilisation [13] and GSIS may determine whether oxidovanadium(IV) complexes have an effect on insulin secretion in INS-1E cells under normo- and hyperglycaemic conditions.

The most potent binders of vanadium(IV) are negatively charged O-donor-containing ligands such as citrate, oxalate, lactate and phosphate. The binding strengths of other serum components such as amino acids and sulphate are negligible, while plasma proteins bind significantly [14]. The work conducted in our laboratory with the imidazolyl–carboxylic acid moiety showed that the [VOL_2_] is the dominant species at neutral pH, suggesting the possibility of the complex being delivered as the un-dissociated form if it survives the digestion process in the stomach [15]. The presence of the carboxylate moiety in the ligands also allows for the interaction with vanadyl ions in the low pH range due to the low pK_a_ and the presence of the imidazole group ensures that complexation remains intact in the neutral pH range since it has an intermediate pK value. In the bloodstream, vanadium also comes into contact and complexes with high-molecular-mass (HMM) proteins [14,16]. Its interaction with serum proteins is an important aspect of metal-based drug metabolism since they are capable of affecting its distribution and biotransformation. The thermodynamic stability should not be weak because the complexes may decompose before it is transported into the circulation of blood plasma, and it should not be too strong because the complexes should be able to be substituted by small bioligands (bLs) and proteins in blood plasma. The design of organovanadium complexes therefore should meet optimum conditions.

Due to the potential application of vanadium complexes in medicine, especially in the treatment of diabetes mellitus (DM), there is considerable interest in the biospeciation of oxidovanadium(IV) complexes to understand the form of the species that might exist under physiological conditions [17]. It would be desirable to design anti-diabetic compounds that activate glucose uptake and at the same time stimulate the release of insulin from pancreatic cells; hence, we embarked on glucose-stimulated insulin secretion (GSIS) studies. The glucose-stimulated insulin secretion activity of oxidovanadium(IV) complexes was studied by comparing their stimulatory indexes in order to confirm their ability to sensitise insulin release from pancreatic cells. This account, therefore, presents the study of the biospeciation of oxidovanadium compounds in simulated blood plasma conditions by investigating the formation of binary and ternary compounds with imidazolyl–carboxylate ligands and biogenic ligands, respectively. The thermodynamic stability of binary and ternary complexes of oxidovanadium(IV) with carrier ligands and biogenic ligands was determined over a pH of 2–11 using potentiometric acid–base titrations [18]. Species distribution diagrams were generated in order to evaluate the species existing over the biological pH range and the species distribution plots of % vanadium as a function of pH were important to evaluate the species that exist under pH conditions in blood (pH = 7.4). Species existing at the biological pH were also determined by EPR, HPLC, LC-MS and MALDI-TOF-MS. The cytotoxicity and glucose-stimulated insulin secretion activity of the vanadium complexes was assessed using pancreatic β-cell lines (INS-1E). The results show promise for the stability of the complexes under biological conditions, and the stimulatory index from GSIS studies is confirmatory of the potential of vanadium complexes to stimulate the release of endogenous reserves of insulin under hyperglycaemic conditions and this has been demonstrated for the first time in this study.

## 2. Results and Discussion

### 2.1. Potentiometry and HYPERQUAD

In order to have a clear understanding of various species existing over the biological pH range, the stability constants approach for chemical speciation was employed. This will also help with an understanding of the necessary species for the antidiabetic effect.

#### 2.1.1. Speciation of Vanadyl Complexes with Small Bioligands

**Figure 2:**
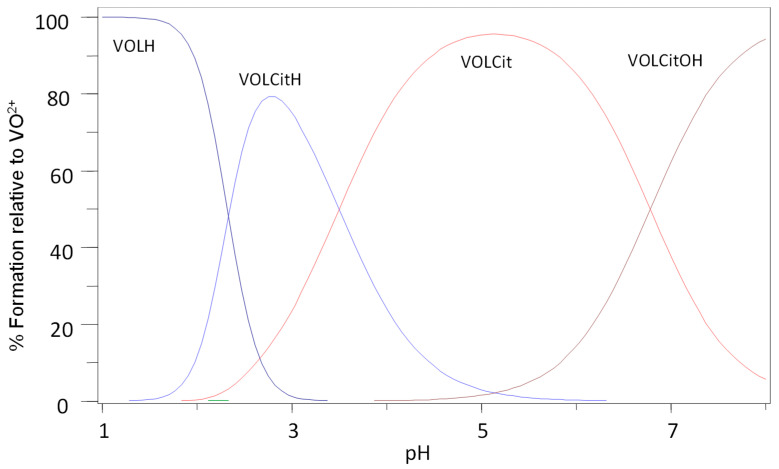
Species distribution diagram for the complexation of VO(IV) with MeIm2COOH (LH) and citric acid (Cit), *C_VO_* = 0.002 mol·L^−1^, VO:L:Cit (1:2:2).

Bioligands such as oxalic acid, citric acid, lactic acid and phosphate are constituents of blood serum, and because of their high affinity for hard metal ions, they are the most likely low-molecular-mass binders of VO(IV). The binding strengths of other serum components such as amino acids and sulphate are negligible. The ternary stability constants of oxidovanadium(IV) with citrate are presented in Table 1. It can be seen from the magnitude of the constants that the ligand system presented herein showed greater stabilisation of the ternary complexes compared with ligand systems presented previously such as 6-methylpicolinic acid, picolinic acid and maltol [19]. The species distribution plot for the VO^2+^-l-Ox system (L = MeIm2COO^−^) is presented in Figure 2, while other ligand systems are presented in the supplementary information (Figures S5–S7).

**Figure 3:**
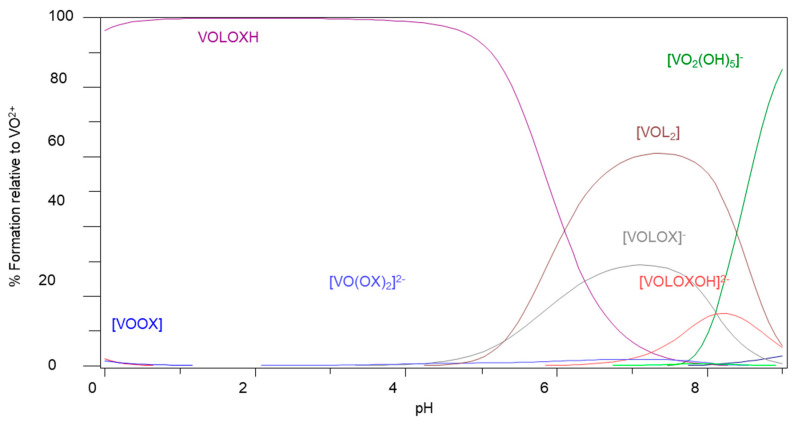
Species distribution diagram for the complexation of VO(IV) with Im4COOH and oxalic acid (Ox), C_VO_ = 0.002 mol·L^−1^, VO:L:Ox (1:2:2).

Once the oxidovanadium(IV) complexes have passed through the digestive tract and have been absorbed into the bloodstream, they may encounter numerous metal-binding bioligands. The species that would be of interest under physiological conditions for MeIm2COOH and citrate are [VOL_2_], [VOLOx]^−^ and [VOLOxOH]^2−^ since they have been shown to exist at pH 7.4. The highest percentage of vanadium is contained by the complex (VOL_2_) at pH 7.4 for Im2COOH/citrate system (Figure S1). This species is also dominant for Im4COOH but co-exists with other species at pH 7.4 such as [VOLOx]^−^ and [VOLOxOH]^2−^ but their percentage is less than that of [VOL_2_] (Figure 3)_._ Ternary complexes formed with other bioligands such as lactate and phosphate also tend to favour the species [VOLbL] and [VOLbLOH] with the former in a higher percentage at pH 7.4 (Figures S5 and S6).

#### 2.1.2. Speciation of Vanadyl Complexes with Proteins

(a) Speciation of VO^2+^-l–human serum albumin (HSA) systems

**Figure 4:**
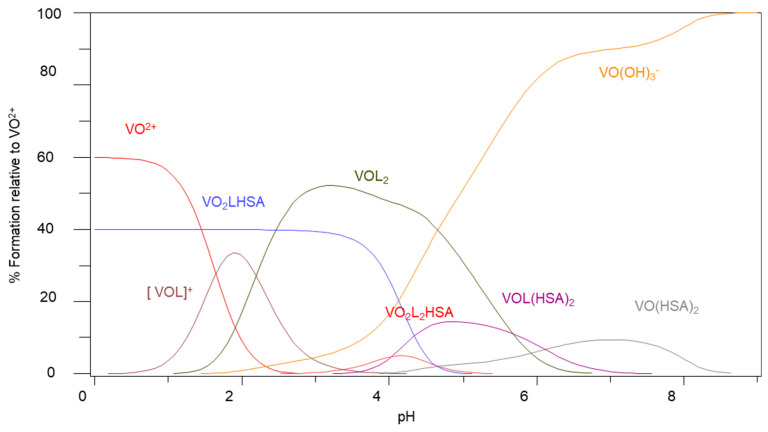
Species distribution diagram for the complexation of VO(IV) with Im4COOH (LH) and human serum albumin (HSA), *C_VO_* = 0.002 mol·L^−1^, VO:L:HSA (1:2:2).

**Figure 5:**
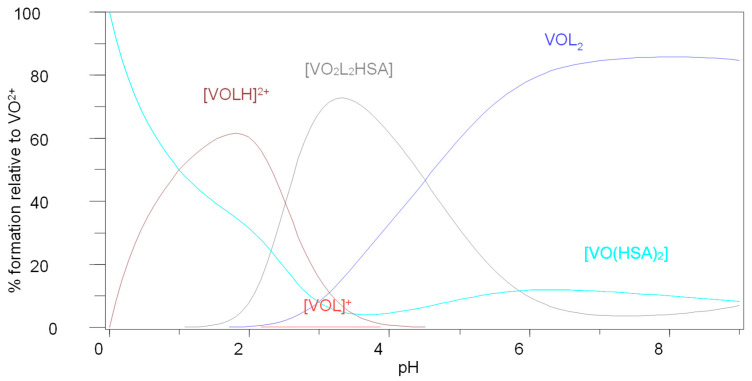
Species distribution diagram for the complexation of VO(IV) with MeIm2COOH (LH) and human serum albumin (HSA), CVO = 0.002 mol·L^−1^, VO:L:HSA (1:2:2).

The species that are of interest are VOL_2_, VOL(HSA)_2_ and VO(HSA)_2_ since they seem to form at pH 7.4 (pH of blood) for Im4COOH and Im2COOH. The presence of the hydrolysis species [VO(OH)_3_]^−^ seems to dominate with Im4COOH or Im2COOH and HSA as coordinating ligands (Figure 4). However, for the N-substituted imidazole–carboxylic system (MeIm2COOH), the species that seem to exist at pH 7.4 are VOL_2_, VO_2_L_2_(HSA) and VO(HSA)_2_ (Figure 5). It seems that the strongly coordinating MeIm2COO^−^ forms the dominant [VOL_2_] at pH 7.4 with the rest of the VO^2+^ being bound to HSA in the forms of VO(HSA)_2_ and VO_2_L_2_HSA.

(b) Speciation of VO^2+^-l–Human serum transferrin (hTf) systems

**Figure 6:**
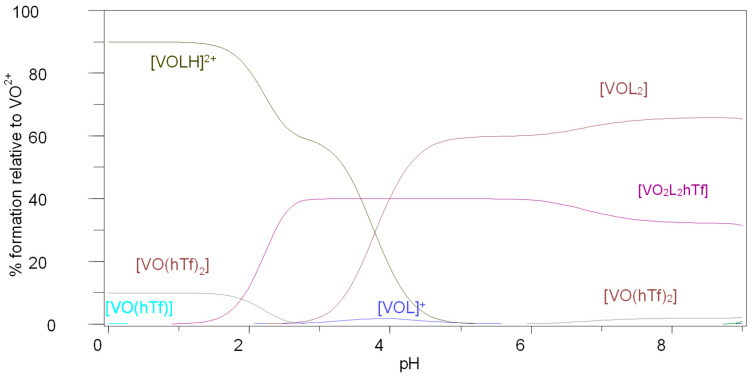
Species distribution diagram for the complexation of VO(IV) with MeIm2COOH (LH) and human serum transferrin (hTf), C_VO_ = 0.002 mol·L^−1^, VO:L:hTf (1:2:2).

The unsubstituted imidazole–carboxylic acid systems (Im4COOH and Im2COOH) share similar stability constants of 19.5 and 19.9, respectively, in the formation of the ternary complex (designated 1,1,1 in Table 2) and are higher than that of maltol. As was observed for HSA as a bioligand, the corresponding N-substituted MeIm2COOH system does not show formation of this species. The MeIm2COOH system also gives a much higher stability constant for the species designated by 2,2,1 and is similar to that of maltol. The constants (2,2,1) are 31.1, 30.8 and 36.4 for Im4COOH, Im2COOH and MeIm2COOH, respectively, as shown in Table 2. The VO-MeIm2COO-hTf system (Figure 6) showed a significant prevalence of ligand-bound vanadyl species ([VOL_2_]), existing over a biological pH range, without the presence of hydrolysis products as observed for the Im4COOH and Im2COOH systems. This is due to the strongly basic nature of the imidazole group in MeIm2COOH compared with other substituted imidazoles used in this study. This study compared the stability constants of the ternary VO-l-hTf systems with other similar ligand systems in terms of structures, especially those with promising anti-diabetic oxidovanadium(IV) compounds containing ligands such as pyridine-2-carboxylic acid (or picolinic acid) and maltol [19]. The stability constants designated as 2,2,1 (Table 2) showed that the ternary complex of VO^2+^-MeIm2COO-hTf possesses a similar stability compared to the oxidovanadium(IV)-maltolato complex.

### 2.2. HPLC, LC-MS and MALDI-TOF-MS

These techniques were used to separate, identify and confirm the species studied using potentiometry and HYPERQUAD. However, only the ternary complexes containing the small bioligands were confirmed by LC-MS (species formed at pH 7.4).

#### 2.2.1. HPLC Studies

(a) Speciation of VO-l–Phosphate systems

There was a shoulder to the peak with a retention time of 3.9 min (Figure S12) and it was assigned to [VOL(H_2_O)_2_]^+^. The peak that appeared at a retention time of 3.9 min was assigned to VOL_2_ and the peak that appeared at a retention time of 4.85 min was assigned to [VOPhos(H_2_O)_2_]^−^, where Phos is the phosphate used to make phosphate buffer solution used to prepare the sample before HPLC analysis. This confirmed the observation of stability constants of phosphate, which showed that it was a stronger binder compared with all the small bioligands investigated in this work. The peak for VO-MeIm2COOH is more intense than that of VO–phosphate perhaps because MeIm2COOH is a stronger binder of vanadyl compared with other imidazole–carboxylic acids.

(b) Speciation of VO-l–Lact system

The investigation of the interaction of oxidovadium(IV) with lactic acid and the carrier ligands using HPLC gave three sharp peaks at retention times of 2.16, 2.96 and 6.97 min for Im4COOH and Im2COOH and 2.18, 2.70 and 6.13 for MeIm2COOH (Figure S15). These peaks, according to LC-MS confirmation, correspond to [VOLLact(OH)]^−^, VOLLact and [VOLPhos]^2−^ where Lact is lactic acid, L is a carrier ligand and Phos is the phosphate ion. Other ternary systems such as VO-l–Cit and VO-l–Ox also showed separations that are in agreement with the species identified by LC-MS (Figures S13 and S14).

(c) Speciation of VO-l-HSA system

The investigation of speciation of oxidovadium(IV) with human serum albumin and the carrier ligands using HPLC gave four sharp peaks at retention times of 2.18, 2.69, 6.2 and 6.3 min for Im4COOH; 2.21, 2.72, 6.34 and 6.44 for Im2COOH; and 2.21, 6.23 and 6.44 for MeIm2COOH systems (Figure S16). These species, according to MALDI-TOF-MS, correspond to [VOPhos(H_2_O)]^−^, [VO(HSA)_2_] and [VOL_2_] as well as [VOL(HSA)_2_] for MeIm2COOH, where HSA is human serum albumin, L is a carrier ligand and Phos is the phosphate ion.

(d) Speciation of VO-l-hTf system

The study of the formation of the ternary complexes of oxidovanadium(IV) with human serum transferrin and the respective ligands using HPLC gave well-separated peaks but one was particularly broad. According to MALDI-TOF-MS, the peaks with retention times around 1.94, 2.68, 5.62 and 7.53 min correspond to [VOL(hTf)_2_]_,_ [VO(hTf)_2_] for Im4COOH and Im2COOH (Figure S17). The third peak was assigned to hydrolysis species such as VO(OH)_3_ and the fourth one was not successfully identified. The peaks at 2.71, 3.88 and 7.69 correspond to [VOL_2_]_,_ [VO_2_L_2_(hTf)] and [VOL(hTf)_2_], respectively, for MeIm2COOH (Figure S17).

#### 2.2.2. LC-MS Studies

The LC-MS studies were conducted in order to complete the assignment of the HPLC peaks and some of these assignments have been briefly covered in the previous section. These species also seem, to some extent, to correspond with the species that have been identified through solution chemistry modelling by potentiometry and HYPERQUAD. The reactions were carried out for 30 min before an LC-MS run.

(a) Speciation of VO-l–Phosphate system

The *m/z* = 289.99 corresponds to [VOL_2_], 214.98 corresponds to [VOL(H_2_O)_2_]^+^ and 279.86 was assigned to [VO(Phos)_2_(H_2_O)_2_]^4−^, where L is a ligand (Im4COOH and Im2COOH) and Phos represents the phosphate ion (Figures S18 and S19). The species at pH 7.4 for Meim2COOH with *m/z* of 226.08 corresponds to [VOL(H_2_O)_2_]^+^, 279.86 was assigned to [VO(Phos)_2_(H_2_O)_2_]^4−^, and 320.03 corresponds to VOL_2_ (Figure S20). These species were also identified using potentiometry and HYPERQUAD. The other peaks are related to the aqua species of oxidovanadium(IV) as well as the phosphate species of vanadium(IV) as expected.

(b) Speciation of VO-l–Lactic acid (Lact) system

The peak at *m/z* = 267.98 was assigned to VOL(Lact) and the peak at *m/z* = 287.12 was assigned to [VOL(Lact)OH]^−^ for Im4COOH and Im2COOH (Figures S27 and S28). The *m/z* = 280.11 corresponds to [VOL(Lact)] and *m/z* = 300.13 was assigned to [VOL(Lact)OH]^−^ for MeIm2COOH (Figure S29). These are the same species that were identified using potentiometry and HYPERQUAD at pH 7.4. The additional peaks are related to the aqua and phosphate species of oxidovanadium(IV); for example, the one at *m/z* = 155 corresponds to [VO(H_2_O)_5_]^2+^ and the one at 279.03 was assigned to [VO(Phos)_2_(H_2_O)]^4−^. Other systems such as VO-l–Citrate (Cit) and VO-l–Oxalate (Ox) have also been similarly characterised for the species existing at pH 7.4 and are presented in appendices (Figures S21–S26).

#### 2.2.3. Speciation of Vanadyl-l-HMM System Using MALDI-TOF-MS

Two main proteins have been proposed to bind and transport metal ions in human serum, namely, albumin and transferrin. It is possible to use MALDI-TOF-MS to study the formation of ternary complexes with carrier ligands and large bioligands [20].

(a) Speciation of vanadyl-l–Human serum albumin (HSA) system

It is well known that MS techniques become more inaccurate in the mass range of proteins under investigation, hence the differences in expected and found mass can be tolerated [20,21]. The peak at 67,000 amu corresponds to [(VO)_2_(L)_2_(HSA)] for (L = Im4COOH), while the one around 134,000 amu corresponds to [VO(HSA)_2_] (Figure S30). Other VO-l-HSA systems are presented in Figures S31 and S32. Other peaks in the MS spectra are due to the fragmentation of the proteins because of the experimental conditions; for example, the laser light energy can cause the analyte to vaporise and may cause the fragmentation of the proteins. These results are in agreement with the results obtained by potentiometry and HYPERQUAD as shown in the species distribution diagrams (for example in Figure 4).

(b) Speciation of vanadyl-l–Human serum transferrin (hTf) system

Figures S33–S35 illustrate the MALDI spectra of VO-l-hTf systems. The peak at 80,000 amu corresponds to (VO)_2_(L)_2_(hTf) for all ligand systems, and the one around 160,000 amu corresponds to VO(hTf)_2_. Other peaks are due to the fragmentation of the proteins and were not identified. These results are in agreement with the species modelled by potentiometry and HYPERQUAD as shown in species distribution diagrams at physiological pH 7.4 (for example in Figure 6).

### 2.3. Speciation of Vanadyl-l–Bioligands Using EPR

EPR spectroscopy examines the transitions between electron spin states separated by the presence of an external magnetic field. The number of lines expected for vanadium species is given by the formula (2*nI* + 1) where *n* is the number of nuclei (one for vanadium) and *I* is the spin (7/2 for vanadium), and eight lines are expected. These spin states are separated by energy, which is dependent on the *g* value of the observed species. For the theoretical free electron, *g* = 2.0023 for vanadium(IV), and this can change when the metal ion is coordinated with ligands. Values of *g* in vanadyl EPR spectra are typically lower than the free electron value. The EPR spin Hamiltonian parameters (A_׀׀_, A_⊥_, g_׀׀_, and g_⊥_) also give information on the geometry of the oxidovanadium(IV) complexes by comparing them with model complexes. The spin Hamiltonian parameters can be calculated from EPR spectra using the computer program developed by Rockenbauer and Korecz [22].

#### 2.3.1. Speciation of Vanadyl-l–Small Bioligand Systems

**Figure 7:**
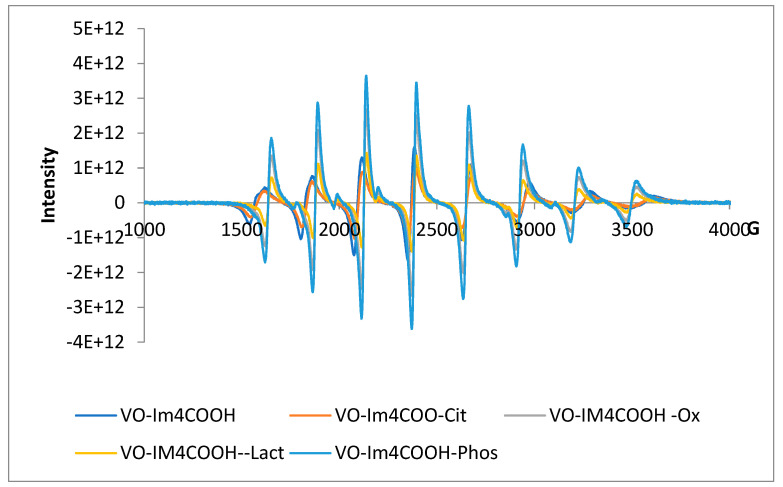
EPR spectra of VO(IV) with Im4COOH (LH) and low-molecular-weight bioligands of human plasma, pH = 7.4.

The EPR spectra of [VO(Im4COOH)_2_], [VO(Im2COOH)_2_] and [VO(MeIm2COOH)_2_] are provided in Figure 7 and Figures S36 and S37, respectively. The hyperfine structures for the nuclear spin of vanadium have parallel coupling constants A_׀׀_ = 167 × 10^−4^ cm^−1^, 165 × 10^−4^ cm^−1^ and 167 × 10^−4^ cm^−1^ for [VO(Im4COOH)_2_], [VO(Im2COOH)_2_] and [VO(MeIm2COOH)_2_], respectively (Table 3). This confirms the presence of the species [VOL_2_] because the values are between 160 × 10^−4^ cm^−1^ for VO-NH-R and 170 × 10^−4^ cm^−1^ for VO-O_2_C-R [23,24]. The perpendicular coupling constants (A⊥) of 60 × 10^−4^ cm^−1^, 60 × 10^−4^ cm^−1^, 58 × 10^−4^ cm^−1^ were observed (Table 3) for [VO(Im4COO)_2_], [VO(Im2COO)_2_] and [VO(MeIm2COO)_2_], respectively. The g_⊥_ values were 1.936, 1.936 and 1.938 for [VO(Im4COO)_2_], [VO(Im2COO)_2_] and [VO(MeIm2COO)_2_], respectively (Table 3), and these are lower than that of [VO(dpp)_2_] (1.986), which has strong ligands resulting in greater stability [24].

Table 3 also shows the EPR parameters of the ternary VO(IV) complexes formed by the chosen carrier ligands and small bioligands. The spectra of VO-Im4COOH-Cit, VO-Im2COOH-Cit and VO-MeIm2COOH-Cit (Figure 7) present the EPR parameters existing between the one presented by VO-L systems and the VO-OH and VO-O_2_CR of citric acid as well as the VO-O_2_C-R of carboxylic acid. For example, the g(⊥) = 1.980, 1.980 and 1.982 for VO-Im4COOH-Cit, VO-Im2COOH-Cit and VO-MeIm2COOH-Cit, respectively. This confirms the presence of the species [VOLCit]^2+^ and [VOLCitOH]^3−^ that exist in physiological pH (7.4) because the g_⊥_ value is 1.979 for VO-O_2_C-Ar from the carboxylic acid group of the ligands (L = Im4COOH, Im2COOH and MeIm2COOH) and 1.980 for citric acid [25]. The g_׀׀_ of 1.943, 1.943 and 1.941 were observed for the complexes VO-Im4COO-Cit_,_ VO-Im2COO-Cit_,_ and VO-MeIm2COO-Cit, respectively, and these are in the same range as VO-O-Ar in [VO(malt)Cit] and [VO(malt)CitOH] (g_׀׀_ = 1.942) [25]. However, the A_׀׀_ values for the VO-l–Cit system are more on the VO–carboxylate end. Other ligand systems (Im2COOH and MeIm2COOH) and bioligands are presented in Figures S36 and S37 as well as Table 3. The parameters for the phosphate system are similar to those of the citrate system, while the lactate system differs in the magnitude of the coupling constants. However, these are not far off the VO–amine system.

#### 2.3.2. Speciation of Vanadyl–Large Bioligand Systems

**Figure 8:**
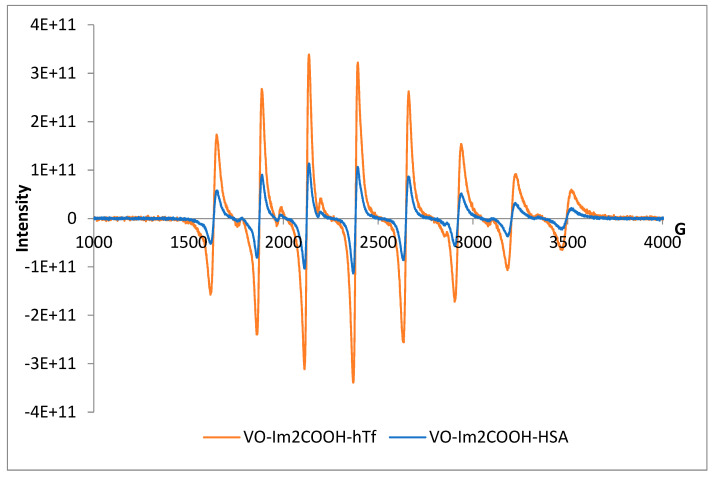
EPR spectra of VO(IV) with Im2COOH (LH) and high-molecular-weight ligands of human plasma, pH = 7.4.

In general, the EPR parameters g_II_ and A_II_ as well as g_⊥_ and A_⊥_ for the ligand MeIm2COOH are higher than those for unsubstituted Im4COOH and Im2COOH for the ternary complexes with human serum albumin and transferrin (Table 4). Spectra are provided in Figure 8 and Figures S38 and S39 for Im2COOH, Im4COOH and MeIm2COOH, respectively. For example, the g_⊥_ values are 1.980, 1.980 and 1.981 for Im4COOH, Im2COOH and MeIm2COOH, respectively. Vanadyl complexes of serum albumin and serum transferrin, which are the major carriers of vanadium in the blood, have been examined using different techniques and species of the form VOL_2_, [VO(HSA)_2_] and [(VO)_2_(L)_2_(HSA)] for human serum albumin, and [VOL_2_], [VO(hTf)_2_], and [(VO)_2_(L)_2_(hTf)] have been identified for human serum transferrin. These species were confirmed by comparing the experimental g⊥ of 1.980, 1.980 and 1.981 for VO-Im4COOH-HSA, VO-Im2COOH-HSA and VO-MeIm2COOH-HSA systems with the one reported for vanadyl–albumin (1.979) [25] as well as 1.936, 1.936 and 1.938 for VO-Im4COOH, VO-Im2COOH and VO-MeIm2COOH, respectively. All the experimental parameters of transferrin systems are slightly higher than those of albumin systems and this is probably due to its VO(IV)-binding ability (Table 4).

### 2.4. Cytotoxicity

**Figure 9:**
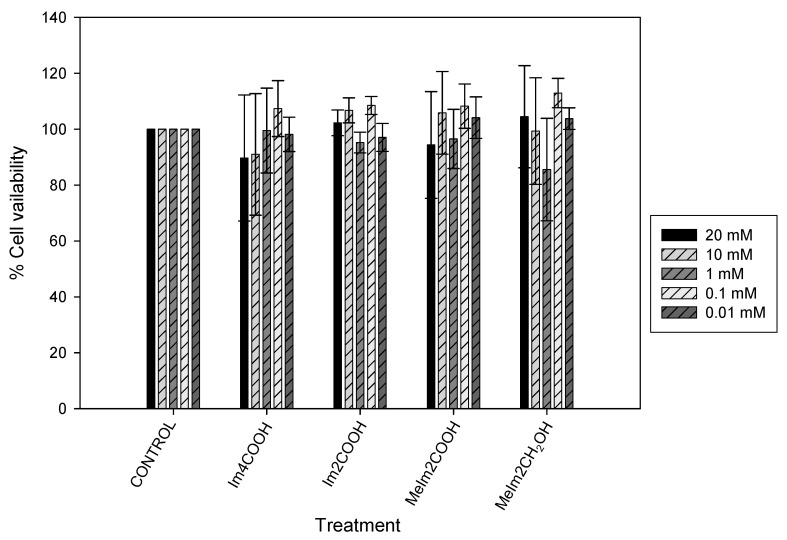
Cell viability of Im2COOH, Im4COOH, MeIm2COOH and MeImCH_2_OH at 20, 10, 1, 0.1, 0.1 and 0.01 µM on INS-1E cells. Error bars indicate SEM (n = 3).

**Figure 10:**
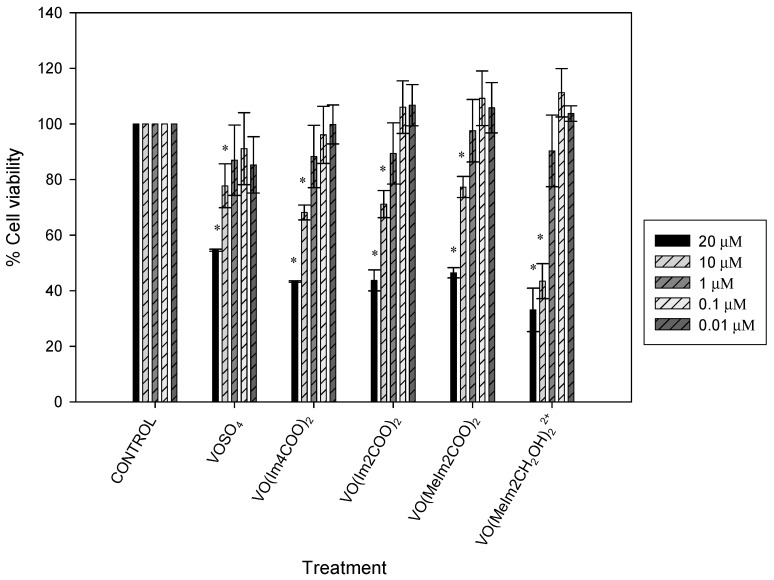
Cell viability of VOSO_4_, VO(Im4COO)_2,_ VO(Im2COO)_2_, VO(MeIm2COO)_2_ and VO(MeImCH_2_O)_2_^2+^ at 20, 10, 1, 0.1, 0.1 and 0.01 µM on INS-1E cells. Error bars indicate SEM (n = 3), * (*p* < 0.05) relative to the control.

The results of cell viability for INS-1E exposed to ligands and oxidovanadium(IV) complexes are presented in Figure 9 and Figure 10, respectively. The concentrations used during this experiment were 20, 10, 1, 0.1 and 0.01 µM for the ligands and oxidovanadium(IV) complexes. The absorbance values that are lower than the control indicate a reduction in the rate of cell proliferation. The values that are higher than the control indicate an increase in cell proliferation and sometimes an increase in proliferation may be offset by cell death. The ligands did not appear to significantly contribute to the toxicity. The oxidovanadium(IV) complexes showed no cytotoxicity between 0.01 and 1 µM in the INS-1E cells tested but showed cytotoxicity from 10 to 20 µM.

The best concentration, which did not affect cell viability, was found to be 1 µM, for all of the oxidovanadium(IV) compounds and, therefore, it was used during this study. The ligand treatments on INS-1E cells did not affect cell viability. The ligands were complexed to vanadium as carriers that assist with the absorption of vanadium into the bloodstream. The ligands would be substituted with biological ligands such as transferrin and albumin during medication intake and for this reason, it was necessary to test the cytotoxicity of ligands in their free form in the INS-1 E cells. Testing for glucose-stimulated insulin secretion using ligands was not necessary in this study.

### 2.5. Glucose-Stimulated Insulin Secretion

The action of vanadium compounds used in this study has been shown to be through PTP inhibition [9]; however, there is still the possibility of other mechanisms acting in parallel with the PTP inhibition mechanisms. As an additional biochemical study, we embarked on the investigation of the effect of the oxidovanadium(IV) compounds on glucose-stimulated insulin secretion and a stimulatory index was calculated [11,12]. From the standard curve generated (Figure S40), the insulin content of each unknown sample was determined.

#### 2.5.1. Chronic Insulin Release

**Figure 11:**
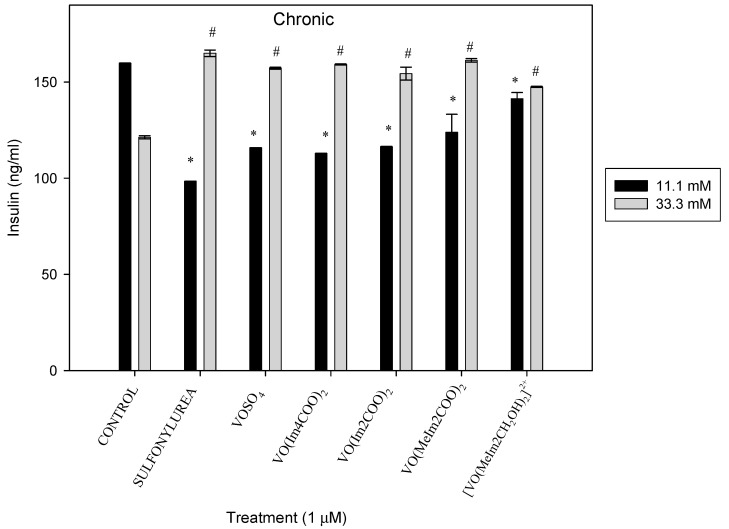
Chronic insulin release after 48 h exposure to the oxidovanadium(IV) complexes (1 µM) in RPMI media containing 11.1 mM and 33.3 mM glucose. * *p* < 0.01 and # *p* < 0.05, indicating significance relative to the 11.1 mM and 33.3 mM glucose control, respectively; error bars indicate SEM (n = 3).

Exposure of islets in 11.1 mM glucose media to the oxidovanadium(IV) complexes for 48 h decreased the insulin secretion of the INS-IE cells by 160, 138, 141, 139, 129 and 113%, (* *p* < 0.01) (1.6-, 1.38-, 1.41-, 1.39-, 1.29- and 1.13-fold, respectively) for the positive control (sulfonylurea: gliclazide), VOSO_4_, VO(Im4COO)_2_, VO(Im2COO)_2_, VO(MeIm2COO)_2_ and VO(MeImCH_2_O)_2_^2+^, respectively. Islet culture in 33.3 Mm glucose media was exposed to the same compounds and it showed an increase in the release of insulin by 136, 129, 131, 127, 133 and 121 % (# *p* < 0.05) (1.36-, 1.29-, 1.31-, 1.27-, 1.33- and 1.21-fold) for the positive control (sulfonylurea: gliclazide), VOSO_4,_ [VO(Im4COO)_2_], [VO(Im2COO)_2_], [VO(MeIm2COO)_2_] and [VO(MeImCH_2_O)_2_^2+^], respectively. Under hyperglycaemic conditions (33.3 mM) (Figure 11), the insulin release was much higher than in normoglycaemic conditions (11.1 mM) and this was expected because chronic hyperglycaemia generally increases insulin release for the positive control (sulfonylurea: gliclazide). All treatments generally increased insulin release as compared to the normoglycaemic levels.

#### 2.5.2. Basal Insulin Secretion

**Figure 12:**
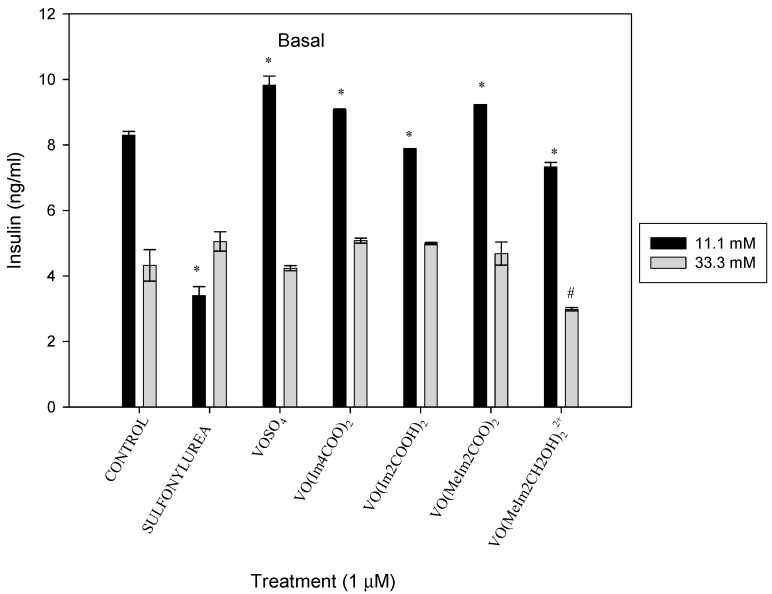
Basal insulin after 48 h exposure to the oxidovanadium(IV) complexes (1 µM) in RPMI media containing 11.1 mM and 33.3 mM glucose. * *p* < 0.01 and # *p* < 0.05, indicating significance relative to the 11.1 mM glucose control and 33.3 mM glucose control, respectively; error bars indicate SEM (n = 3).

INS-1E cells cultured in 11.1 mM glucose media and exposed to the oxidovanadium(IV) compounds had a decreased basal insulin secretion by 2.8-fold, 1.05-fold and 1.13-fold for the positive control (sulfonylurea), [VO(Im2COO)_2_] and [VO(MeImCH_2_O)_2_^2+^], respectively, and an increased basal insulin secretion by 1.12-fold, 1.09-fold and 1.11-fold for VOSO_4,_ [VO(Im4COO)_2_] and [VO(MeIm2COO)_2_] relative to the respective untreated normoglycaemic conditions, (* *p* < 0.01). This has also been observed by Mnonopi upon treatment of INS-1 cells with marrubin [26]. There was no effect of the oxidovanadium(IV) complexes on the islet culture under hyperglycaemic conditions (33.3 mM) except for the cationic compound, [VO(MeImCH_2_O)_2_^2+^], which showed a decrease in insulin by 1.45-fold (Figure 12).

#### 2.5.3. Stimulated Insulin Secretion

**Figure 13:**
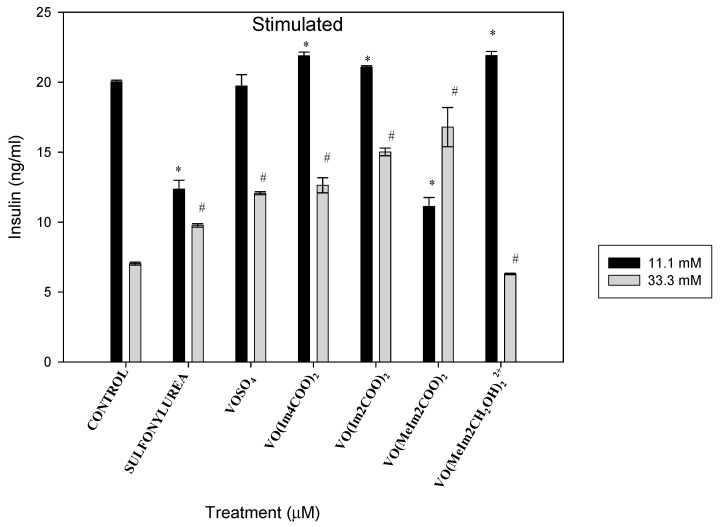
Stimulated insulin secretion after 48 h exposure to the oxidovanadium(IV) complexes (1 µM) in RPMI media containing 11.1mM and 33.3 mM glucose. * *p* < 0.01 and # *p* < 0.05, indicating significance relative to the 11.1mM and 33.3mM glucose control, respectively; error bars indicate SEM (n = 3).

Pancreatic INS-1E cells exposed to the oxidovanadium(IV) complexes (1 µM) showed the same trend in stimulating the insulin control in 11.1 mM glucose media, with the exception of sulfonylurea (gliclazide) and [VO(Meim2COO)_2_], where insulin stimulation was slightly lower than the untreated control as well as lower than other compounds by 1.62-fold and 1.79-fold, respectively. The islets cultured in hyperglycaemia media of 33.3 mM glucose concentration increased the insulin stimulation by 1.39, 1.72, 1.8, 2.14 and 2.39-fold, respectively, for the positive control (sulfonylurea: gliclazide), VOSO_4,_ [VO(Im4COO)_2_], [VO(Im2COO)_2_] and [VO(MeIm2COO)_2_] relative to the relevant control cells (# *p* < 0.05). The cationic compound, [VO(MeImCH_2_O)_2_^2+^], decreased the insulin by 111% (1.11-fold), as shown in Figure 13.

#### 2.5.4. Insulin Content

**Figure 14:**
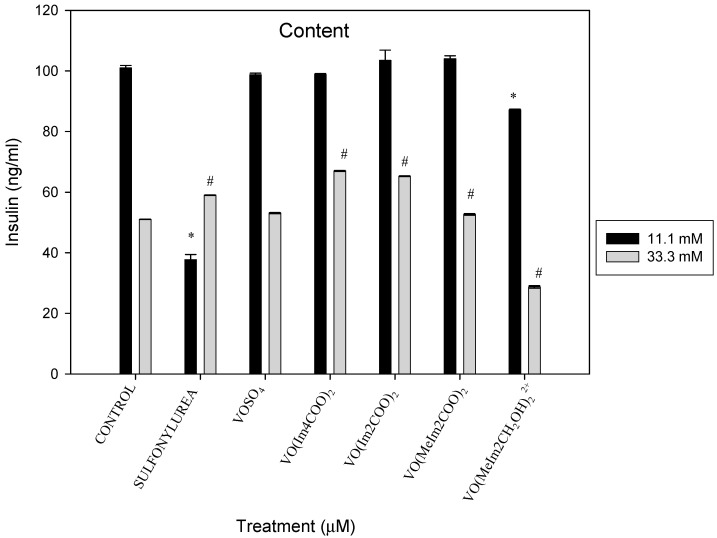
The insulin content of the islets after 48 h exposure to the oxidovanadium(IV) complexes (1 µM) in RPMI media containing 11.1 mM and 33.3 mM glucose (* *p* < 0.01 and # *p* < 0.05), indicating significance relative to the 11.1 mM glucose control and 33.3 mM glucose control, respectively; error bars indicate SEM (n = 3).

The 11.1 mM INS-1E cells exposed to the test compounds showed similar insulin content to that of the relevant control (Figure 14) islets except for the cationic compound, [VO(MeImCH_2_O)_2_^2+^], which decreased the insulin content by 115% relative to the control (* *p* < 0.01). Under hyperglycaemic conditions (33.3 mM glucose media), the oxidovanadium(IV) compounds significantly increased insulin relative to the control (# *p* < 0.05) for sulfonylurea, [(VO(Im4COOH)_2_] and [VO(Im2COOH)_2_] by 1.15, 1.31 and 1.27-fold, respectively. The salt (VOSO_4_) and [VO(MeIm2COO)_2_] had the same trend as the control and the cationic compound decreased insulin release by 1.77-fold.

#### 2.5.5. Stimulatory Index

**Figure 15:**
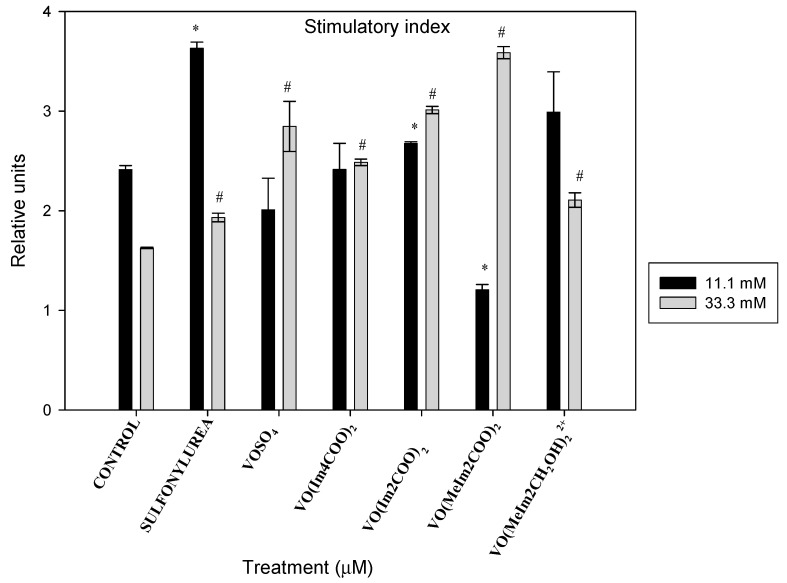
The stimulatory index of the islets after 48 h exposure to the oxidovanadium(IV) complexes (1 µM) in RPMI media containing 11.1 mM and 33.3 mM glucose (* *p* < 0.01 and # *p* < 0.05), indicating significance relative to the 11.1 mM glucose control and 33.3 mM glucose control, respectively; error bars indicate SEM (n = 3).

In the INS-1E cells in 11.1 mM glucose media, the oxidovanadium(IV) compound showed a significant increase in the stimulatory index relative to the control by 1.51-fold for sulfonylurea and decreased the insulin release by 2-fold for [VO(MeIm2COO)_2_], and others had the same trend as the control (* *p* < 0.01). However, the islets exposed to oxidovanadium(IV) complexes under hyperglycaemic conditions (33.3 mM glucose media) displayed a significant increase in the stimulatory index: 1.19, 1.75, 1.53, 1.85, 2.20 and 1.29 for the positive control (sulfonylurea), VOSO_4,_ [VO(Im4COO)_2_], [VO(Im2COO)_2_], [VO(MeIm2COO)_2_] and [VO(MeIm2CH_2_OH)_2_^2+^], respectively (# *p* < 0.05). Figure 15 illustrates the stimulatory index of the islets after 48 h exposure to the oxidovanadium(IV) complexes (1 µM) in RPMI media containing 11.1 mM and 33.3 mM glucose. The work completed showed that under normoglycaemic conditions (11.1 mM), there is not much effect related to insulin release by the oxidovanadium(IV) compounds on the cells unlike when using sulfonylurea. However, there is a significant increase in insulin secretion under hyperglycaemic conditions with [VO(MeIm2COO)_2_], proving it to be the best compound for this application.

## 3. Experimental Section

### 3.1. Materials and Instrumentation

Oxidovanadium(IV) sulphate hydrate was obtained from BDH Limited (Brighouse, UK). 1-Methylimidazole (MeIm) (99%) and Im4COOH (98%) were obtained from Sigma-Aldrich (Burlington, MA, USA). Imidazole-2-carboxaldehyde (97%) was obtained from Fluka (Ballwin, MO, USA). All solvents were obtained from Merck Chemicals (SA) (Madrid, Spain) and were of reagent grade and used without further purification. Other reagent-grade chemicals were also obtained from commercial sources and used as received. ELISA kits, human serum albumin (98% globulin-free), human serum transferrin (97%, iron-free), lactic acid (99%), oxalic acid (99%), phosphoric acid (99%) and sulfonylurea were obtained from Merck. RPMI-1640 medium, tetramethylammonium chloride and tetramethylammonium hydroxide were obtained from SIGMA Aldrich. MALDI chromatograms were recorded on a Bruker AutoFLEX III Smart beam TOF/TOF mass spectrometer. EPR spectra were recorded on a Bruker BioSpin GmbH Electro paramagnetic spectroscope. HPLC chromatograms were recorded on an Agilent 1100 High-Performance Liquid Chromatograph (HPLC) fitted with a DAD detector and the Kinetex 2.6 µm PFP 100A (150 × 4.60 mm) column. Chromatographic separations were also carried out on a liquid chromatograph equipped with an ion trap mass spectrometer (LCQ Duo, Thermo Finnigan, San Jose, CA, USA) with an electrospray (ES) ion source fitted with an LCQ detector and the Kinetex 2.6 µm PFP 100A (150 × 4.60 mm) column. A Bio-Tek KC4 Powerwave XS microtiter plate reader was used to measure 3-[4,5-dimethylthiazol-2-yl]-2,5-diphenyltetrazolium bromide (MTT) and for the glucose-stimulated insulin secretion studies. Potentiometric studies were performed with a Metrohm 888 Basic Titrando equipped with Tiamo software version 2.3, a Metrohm LL electrode and a stirrer. A Haake SC100 thermoregulator (equipped with a Haake A28 water bath) was used to maintain a constant temperature during the titration process. Nitrogen gas was used to control the medium and to make sure that V(IV) was not oxidised to V(V). The pH measurements were performed on a Metrohm 827 pH meter.

### 3.2. Preparative Work

The ligands and oxidovanadium(IV) complexes have been synthesised and characterised previously [9]. However, some characterisation data towards the reproduction of the compounds is also available in the supplementary information.

### 3.3. Potentiometric Studies

The protonation and stability constants for the ligands and oxidovanadium(IV) complexes were determined by potentiometric titration of approximately 25 mL samples. All solutions were prepared using freshly boiled and degassed deionised Milli-Q water to ensure the removal of dissolved oxygen and carbon dioxide. The ratios of metal:ligand:bioligand of 1:2:1, 1:2:2 and 1:2:6 were used in duplicate, respectively. Titrations were performed over the pH range of 2–11 under a continuous flow of purified nitrogen using HCl and tetramethylammonium hydroxide (TMAOH). The vanadium stock solution containing 0.10 M HCl was standardised by titration with permanganate. The ionic strength of the titration solutions was kept constant at 0.10 M tetramethylammonium chloride (TMACl). Titrations were controlled using Tiamo software, the titration rate used was 0.01 mL/min and the pausing time was 60 s. The glass electrode was calibrated for a strong acid–base reaction by the Gran method [27] using the program GLEE [28] to determine the standard potential E°. The ionic product of water (pK_w_) of 13.83(1) at 25.0 ± 0.1 °C in 0.10 M TMACl was used in all calculations [29]. The hydrolysis model of an oxidovanadium(IV) system was included in the model ([VO(OH)_3_] − (logβ = −18.0) and [(VO)_2_(OH)_5_] − (logβ = −22.0)), while [VO(OH)]^+^ (logβ = −5.94) and [(VO)_2_(OH)_2_]^2+^ (logβ = −6.95) [30] did not fit. The concentration stability constants β_pqr_ = [MpLqHr]/[M]p [L]q [H]r were calculated by using the computer program HYPERQUAD [31].

### 3.4. Electron Paramagnetic Resonance Spectroscopy

The hyperfine structure of EPR spectra is sensitive to small structural changes in the complexes; hence, it was used to investigate the speciation of oxidovanadium(IV) complexes with low and high molecular ligands of human plasma. The parameters that gave well-resolved spectra at pH 7.4 are as follows: microwave frequency of 9.716, power of 20.000 mV, central field of 35,000.000 G, sweep width of 2000.000 G and a sweep time of 100.000 s.

### 3.5. High-Performance Liquid Chromatography (HPLC) and Liquid Chromatography–Mass Spectrometry (LC-MS)

#### 3.5.1. High-Performance Liquid Chromatography (HPLC)

This technique was used to separate the binary and ternary oxidovanadium(IV)-l-bL species (L = ligand, and bL = bioligands). This study was carried out to complement the pH-metric chemical speciation modelling study performed on the VO-l-bL system by potentiometry and HYPERQUAD.

#### 3.5.2. Liquid Chromatography–Mass Spectrometry (LC-MS)

In this study, this technique was used to identify oxidovanadium(IV) complexes formed with different small bio-ligands. Quantification was not completed since there were no available standards for these species. A Kinetex 2.6 µm PFP column was used for this isocratic method. Twenty-microliter samples were injected and the mobile phase was 0.01 M phosphate buffer:methanol (8:2) (pH 7.4). The run time was 15 min at a flow rate of 0.6 mL/min. The wavelength used was 250 nm on the DAD (Diode Array Detector).

### 3.6. Matrix-Assisted Laser Desorption Ionisation–Time of Flight-Mass Spectrometry (MALDI-TOF-MS)

In MALDI analysis, the analyte is first co-crystallised with a large molar excess of a matrix compound, usually a UV-absorbing weak organic acid [32]. Then, the laser radiation of this analyte–matrix mixture results in the vaporisation of the matrix that carries the analyte with it. The matrix therefore plays an important role of strongly absorbing the laser light energy and this in turn causes the analyte to vaporise. The matrix also serves as a proton donor and receptor, acting to ionise the analyte in both positive and negative ionisation modes, respectively. Sinapinic acid was used as a matrix for analysing VO-l-bL systems (where bL refers to high-molecular-weight bioligands in this case). Quantification was not performed since there are no available standards for these species, and the operating parameters that gave us good resolution were a wavelength of 355 nm, Nd:YAG laser as an ionising source and positive ion mode.

### 3.7. Biological Studies

The in vitro testing for the biological anti-diabetic activity of the oxidovanadium(IV) complexes was completed as follows: Pancreatic cell culture lines (INS-1E) were used to investigate the potential of the oxidovanadium(IV) complexes on glucose-stimulated insulin secretion (GSIS). Cell viability tests were performed before the glucose-stimulated insulin secretion studies to establish non-toxic concentrations of the oxidovanadium(IV) compounds.

#### 3.7.1. Maintenance of Cells

Pancreatic cells (INS-1E) were maintained in RPMI-1640 medium (Sigma, Fukushima, Japan) supplemented with 10% FBS. These were incubated at 37 °C in a humidified incubator with 5% CO_2_. Cells were subcultured at 70% confluence and seeded at a density of 50,000 cells/well (for INS-1E) in 96-well culture plates. They were supplemented with RPMI 1640 media containing glutamax, 5% fetal bovine serum (FBS, Belize City, Belize), 10 mM HEPES, 50 mM 2-mercaptoethanol and 1 mM sodium pyruvate. When the cells reached 70% confluency, they were subcultured using a 1:3 split ratio. The INS-1E cells were cultured in both normoglycaemic (11.1 mM glucose) and hyperglycaemic conditions (33.3 mM glucose).

#### 3.7.2. Cytotoxicity (MTT) Assay

The MTT–cell proliferation assay is a quantitative colourimetric assay for measurements of cellular proliferation, viability and cytotoxicity [33,34,35]. The assay is based on the cleavage of the yellow tetrazolium salt, MTT, which forms water-insoluble, dark blue formazan crystals. This cleavage only takes place in living cells by the mitochondrial enzyme succinate-dehydrogenase [35]. The water-insoluble formazan can be solubilised using isopropanol or another organic solvent. The optical density of the dissolved material is measured spectrophotometrically, yielding absorbance as a function of the concentration of converted dye, which directly correlates to the number of metabolically active cells in the culture [33]. Cells were seeded in 96-well plates (Nunc, Merck) at densities of 50,000 for INS 1E cells/mL/well. After overnight attachment, the culture medium was replaced with a medium containing the test compounds at a range of concentrations (0.01–20 μM). Cells were incubated for 48 h at 37 °C, after which the MTT assay was performed [33].

#### 3.7.3. Glucose Stimulation Insulin Secretion

The glucose-stimulated insulin secretion (GSIS) is controlled by glucose-derived signals in pancreatic β-cells [12]. During hyperglycaemic conditions, insulin enhances glucose uptake and utilisation [13]. GSIS was performed to determine whether oxidovanadium(IV) complexes as well as vanadyl sulphate (vanandium salt) had an effect on insulin secretion in INS-1E cells under normo- and hyperglycaemic conditions. For all the experiments completed, pancreatic β-cells (INS-1E) were seeded and incubated in RPMI+glutamax media containing glucose concentrations of 11.1 mM at 33.3 mM for 48 h (chronic). This media was replaced with media containing low (16.3 mM) and high (33.3 mM) glucose concentrations (one hour each) as well as the test compounds (oxidovanadium compounds, and vanadium salt) and sulfonylurea as a positive control. The total insulin (content) as well as the insulin secreted under basal (3.3 mM) and stimulated (16.3 mM) conditions was quantified using an insulin ELISA KIT (Merck). The stimulatory index was determined using the following Equation (1):

Stimulatory index = glucose stimulated insulin secretion ÷ basal insulin secretion.(1)

#### 3.7.4. Statistical Analysis

Error bars indicate the standard error of the mean (SEM) unless specified otherwise (n = 3). The two-tailed paired test was used to determine the significance of the results: (*p* < 0.05) and (*p* < 0.01).

## 4. Conclusions

The stability constant studies for binary and ternary complexes of oxidovanadium(IV) complexes with imidazolyl–carboxylate and small bioligands such as oxalate, citrate, lactate and phosphate were studied using potentiometry and solved with HYPERQUAD. The species distribution diagrams were dominated by ternary species of the types [VOL_2_], [VOL(bL)] and [VOL(bL)OH] identified under physiological pH when small bioligands were involved. The interaction of the vanadyl ion and the chosen carrier ligands in the presence of large bioligands such as human serum albumin and transferrin was also studied by potentiometry, HPLC, LC-MS, MALDI-TOF-MS and EPR. The dominant species seem to be (VO)_2_(L)_2_(bL) and VO(bL)_2_ under physiological pH for the large bioligands. The human serum transferrin showed a strong vanadyl-binding ability compared to human serum albumin, and the use of MeIm2COOH as a carrier ligand seems to present a strong competitor for binding vanadyl. It can, therefore, be concluded that if the complex ought to stay in the complexed form (with the carrier ligand) for the prolonged activity of vanadium compounds, then the use of the VO(MeIm2COO)_2_ complex in the treatment of diabetes mellitus would be promising.

Oxidovanadium(IV) complexes reduced insulin stimulation significantly under normoglycaemic levels (11.1 mM glucose media) compared with sulfonylurea, and these complexes showed positive effects on insulin secretion under hyperglycaemic conditions (33.3 mM glucose media). The islets exposed to oxidovanadium(IV) complexes under hyperglycaemic conditions (33.3 mM glucose media) displayed a significant increase in the stimulatory index with 1.19, 1.75, 1.53, 1.85, 2.20 and 1.29 for the positive control (sulfonylurea:gliclazide), VOSO_4,_ VO(Im4COO)_2_ VO(Im2COO)_2_, VO(MeIm2COO)_2_ and VO(MeIm2CH_2_OH)_2_^2+^, respectively. It can be seen that VO(MeIm2COO)_2_ almost always performed better than other neutral complexes such as VO(Im4COO)_2_ and VO(Im2COO)_2_, as well as the salt (VOSO_4_) and the cationic complex [VO(MeIm2CH_2_OH)_2_]^2+^. The vanadium compounds presented herein are also superior to gliclazide (sulfonylurea) in promoting insulin secretion under hyperglycaemic conditions while reducing insulin secretion under normoglycaemic conditions. This result shows another potential advantage for the use of oxidovanadium(IV) complexes in the treatment of diabetes mellitus type II.

## Figures and Tables

**Table 1:** Stability constants for ternary complexes of small bioligands (bL = oxalate, citrate, phosphate or lactate) and ligands (L = Im4COOH, Im2COOH or MeIm2COOH) with VO^2+^ ions at 25(±0.1) °C and *I* = 0.10 M (N(ME)_4_Cl). Comparison with other V^IV^-(6-methylpicolinic acid, picolinic acid, maltol) systems [19] is also presented. Standard deviations are reported in parentheses.

	VO^2+^, L, bL, H	Im4COOH	Im2COOH	MeIm2COOH	Mal ^†^	Mepic ^†^	Pic ^†^
Oxalate	1,1,1,1	18.9(0.1)	18.79(0.07)	19.86(0.03)	–	–	–
	1,1,1,0	12.2(0.1)	12.1(0.6)	12.89(0.04)	13.92(0.0)	11.22(0.02)	12.38(0.02)
	1,1,1,−1	5.4(0.1)	5.8(0.1)	5.79(0.07)	–	–	–
Citrate	1,1,1,1	16.7(0.1)	16.39(0.07)	16.36(0.03)	–	–	–
	1,1,1,0	9.5(0.1)	9.3(0.6)	9.79(0.04)	–	–	–
	1,1,1,−1	3.5(0.1)	3.8(0.1)	3.69(0.07)	–	–	–
Phosphate	1,1,1,1	27.35(0.02)	24.16(0.03)	27.42(0.04)	25.00(2)	20.94(4)	22.73(11)
	1,1,1,0	20.37(0.07)	18.26(0.04)	18.80(0.02)	18.81(2)	15.40(2)	15.52(34)
	1,1,1,−1	13.53(0.01)	10.09(0.04)	10.80(0.08)	–	–	–
Lactate	1,1,1,1	21.83(0.04)	20.98(0.04)	22.27(0.03)	–	–	–
	1,1,1,0	17.88(0.03)	17.11(0.04)	17.54(0.03)	–	–	–
	1,1,1,−1	12.78(0.04)	12.78(0.04)	13.86(0.09)	–	–	–

**^†^** *I* = 0.20 M (KCl).

**Table 2:** Stability constants of ternary complexes of VO^2+^-hTf-Im4COOH, Im2COOH and MeIm2COOH as well as VO^2+^-HSA-Im4COOH, Im2COOH and MeIm2COOH at 25(±0.1) °C; *I* = 0.10 M (N(ME)_4_Cl).

VO^2+^,L,hTf	Im4COOH	Im2COOH	MeIm2COOH	Mal ^†^
1,1,1	19.5	19.9	-	17.7
2,2,1	31.1	30.8	36.4	34.8
2,1,1	27.4	25.7	27.6	30.3
**VO,L,HSA**	**Im4COOH**	**Im2COOH**	**MeIm2COOH**	
1,1,1	17.3	17.4	-	
2,2,1	28.5	27.9	32.5	
2,1,1	24.3	23.7	24.7	

^†^ I = 0.20 M (KCl) and mal = maltol.

**Table 3:** EPR parameters of VO(IV) complexes formed by small bioligands.

Mode of Speciation	g_⊥_	*g*_׀׀_	A_⊥_ (10^−4^ cm^−1^)	A_׀׀_ (10^−4^ cm^−1^)
VO^2+^-Im4COOH	1.936	1.936	60.1	167.0
VO^2+^-Im2COOH	1.936	1.936	60.1	165.0
VO^2+^-MeIm2COOH	1.938	1.935	58.9	167.0
VO^2+^-Im4COO-Cit	1.980	1.943	59.7	170.6
VO^2+^-Im2COO-Cit	1.982	1.943	58.5	171.2
VO^2+^-MeIm2COO-Cit	1.980	1.941	60.1	174.0
VO^2+^-Im4COOH-Ox	1.938	1.966	98.0	172.0
VO^2+^-Im2COOH-Ox	1.938	1.966	98.0	172.0
VO^2+^-MeIm2COOH-Ox	1.939	1.967	98.0	171.0
VO^2+^-Im4COOH-Lact	1.972	1.950	-	161.1
VO^2+^-Im2COOH-Lact	1.972	1.951	-	161.7
VO^2+^-MeIm2COOH-Lact	1.974	1.951	-	162.1
VO^2+^-Im4COOH-Phos	1.980	1.940	-	171.0
VO^2+^-Im2COOH-Phos	1.980	1.940	-	171.7
VO^2+^-MeIm2COOH-Phos	1.981	1.938	-	170.8

**Table 4:** EPR parameters of VO(IV) complexes formed by high-molecular-weight bioligands.

Mode of Speciation	g_⊥_	*g*_׀׀_	A_⊥_ (10^4^ cm^−1^)	A_׀׀_ (10^4^ cm^−1^)
VO^2+^-Im4COOH-HSA	1.980	1.938	62.0	168.1
VO^2+^-Im2COOH-HSA	1.980	1.938	61.0	168.0
VO^2+^-MeIm2COOH-HSA	1.981	1.938	62.0	169.0
VO^2+^-Im4COOH-hTf	1.985	1.940	63.0	169.0
VO^2+^-Im2COOH-hTf	1.985	1.940	63.0	169.0
VO^2+^-MeIm2COOH-hTf	1.987	1.941	63.0	170.0

## Data Availability

All data are presented in the manuscript and in the Supplementary Information.

## Supplementary Materials

The following supporting information can be downloaded at: https://www.mdpi.com/article/10.3390/molecules29030724/s1. Refs. [36,37,38,39,40,41] are cited in Supplementary Materials.
