# Supplementary material for: Biospeciation of Oxidovanadium(IV) Imidazolyl–Carboxylate Complexes and Their Action on Glucose-Stimulated Insulin Secretion in Pancreatic Cells
Source: Molecules. 2024 Feb 4;29(3):724. doi: 10.3390/molecules29030724 (PMC10856044; doi:10.3390/molecules29030724)
Supplement: Supplementary file 1 [file molecules-29-00724-s001.zip › molecules-2677060-supplementary.pdf]

## Supplementary information

# Biospeciation of Oxidovanadium(IV) Imidazolyl–Carboxylate Complexes and Their Action on Glucose-Stimulated Insulin Secretion in Pancreatic Cells

**Vital Ugirinema [1,2,*], Frank Odei-Addo [3], Carminita L. Frost [3,*] and Zenixole R. Tshentu [1,*]**

[1] Department of Chemistry, Nelson Mandela University, P.O. Box 77000, Port Elizabeth 6031, South Africa
[2] Department of Chemistry, College of Science and Technology, University of Rwanda, Kigali P.O. Box 3900, Rwanda
[3] Department of Biochemistry and Microbiology, Nelson Mandela University, P.O. Box 77000, Port Elizabeth 6031, South Africa; frank.odeiaddo@gmail.com
* Correspondence: vital.ugirinema@gmail.com (V.U.); carminita.frost@mandela.ac.za (C.L.F.); zenixole.tshentu@mandela.ac.za (Z.R.T.)

## 1. Preparative work

This work on preparation of ligands and oxidovanadium(IV) complexes has been published before by us [1] but confirmatory details are provided below:

### 1.1 Synthesis of ligands

#### 1.1.1 Imidazole-4-carboxylic acid (Im4COOH)

This ligand (Im4COOH), was purchased from Sigma-Aldrich Chemical Company and used in subsequent steps without further purification.

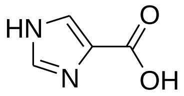


#### 1.1.2 Methylimidazole-2-carboxaldehyde (MeIm2CHO)

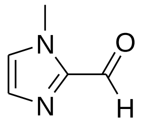


1-Methylimidazole-2-carboxaldehyde was prepared according to a literature method [2,3,4]. To a suspension of 1-methylimidazole (2.20 g, 0.020 mol) in dry diethyl ether (50 mL) was added 2.5 M butyllithium (8.40 mL, 0.021 mol) at -78 °C. After stirring for an hour, DMF (2.33 mL, 0.030 mol) was added and this solution was stirred overnight. After completion of the reaction, 2 mL of water was added followed by 15 mL of 4 N HCl. The aqueous layer was made basic by addition of potassium carbonate following which the product was extracted into chloroform. This organic layer was concentrated and the product was distilled at $3.0 \times 10^{-1}$ mBar and 80°C to yield a brownish crystalline solid after cooling. Yield: 76.1%. $^1$H ($\delta$, 400 MHz, $CDCl_3$): 4.03 (s, 3H, $NCH_3$), 7.16-7.27 (m, 2H, Im-H), 9.81 (s, 1H, CHO); $^{13}$C NMR ($\delta$, 400 MHz, $CDCl_3$): 35.20, 127.69, 131.74, 144.00, 182.37. IR (cm$^{-1}$, neat): 3200 ν(O-H); 1637 ν(C=N); 1400 ν(C=N-C=N). *Anal.* Calcd (Found) for $C_5H_6N_2O$ (%): C, 54.54 (54.07); H, 5.49 (5.52); N, 25.44 (25.28).

### 1.1.3 Imidazole-2-carboxylic acid.$H_2O$ (Im2COOH)

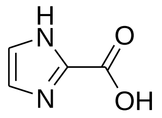

An aqueous 30% $H_2O_2$ (10 g) was added dropwise to a stirred solution of imidazole-2-carboxaldehyde (2.88 g, 0.030 mol) in water (10 mL). The reaction was allowed to proceed at room temperature for 72 hours, following which the water was removed *in vacuo* at room temperature to afford a white crystalline solid. This solid was washed with a mixture of diethyl ether/methanol/water (4:1:1) to remove the excess peroxide. *Note*: The product should be stored in an anhydrous environment since it is hygroscopic. Slow decarboxylation may occur at room temperature, thus refrigeration is suggested. Yield: 97.5%. Mp = 156-158 °C. $^{1}$H NMR ($\delta$, 400 MHz, $D_2O$): 7.56 (2H, s, Im-H); $^{13}$C NMR ($\delta$, 400 MHz, $D_2O$): 158.86, 141.02, 120.49. IR (cm$^{-1}$, neat): 3400 ν(O-H); 1637 ν(C=N); 1450 ν(C=N-C=N). *Anal.* Calcd (Found) for $C_4H_6N_2O_3$ (%): C, 36.92 (37.18); H, 4.65 (4.94); N, 21.53 (21.47).

### 1.1.4 1-Methylimidazole-2-carboxylic acid.$H_2O$ (MeIm2COOH)

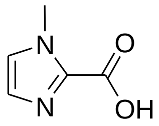

The procedure followed that of Im2COOH above except that 1-methylimidazole-2-carboxyaldehyde was used and the yield was quantitative after the removal of water under high vacuum (no washing with diethyl ether/water was necessary). *Note*: The product should be stored in an anhydrous environment since it is hygroscopic. Slow decarboxylation may occur at room temperature, thus refrigeration is suggested. Yield: 100%. Mp = 99-101 °C. $^{1}$H NMR ($\delta$, 400 MHz, $D_2O$): 7.42, 7.39 (2H, s, Im-H) and 4.08 ppm (3H, s, $NCH_3$); $^{13}$C NMR ($\delta$, 400 MHz, $D_2O$): 158.67, 139.68, 125.83, 118.46, 36.73. IR (cm$^{-1}$, neat): 3408 ν(O-H); 1637 ν(C=N); 1480 ν(C=N-C=N). *Anal.* Calcd (Found) for $C_5H_8N_2O_3$ (%): C, 41.67 (41.28); H, 5.59 (5.23); N, 19.44 (19.12).

### 1.1.5 2-Hydroxymethyl-1-methylimidazole

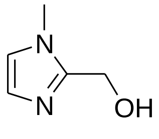

To a stirred solution of the 1-methylimidazole-2-aldehyde (3.4 mmol) in methanol (10ml) at 0°C was added $NaBH_4$ (3.4 mmol). After stirring for 2.5 hours the solution was concentrated. The residue was taken up in $Et_2O$ and extracted 3 times with water. The ethereal solution was dried with $Na_2SO_4$, filtered and $Et_2O$ was removed in *vacuo* to obtain a pure product. Yield 56.1 %. White crystaline solid, m.p. 107–110 °C, 1H NMR (400 MHz, CDCl3): C 3.75 (3H, s, NCH3), 4.66 (2H, s, CH2OH), 6.84, 6.89 (2H, s, Im-H); 13C NMR (400 MHz, CDCl3): C 32.97, 55.60, 121.58, 126.65, 148.28. IR (cm$^{-1}$, neat): 3417 ν(O-H); 1637 ν(C=N); 1499 ν(C=N-C=N). *Anal.* Calcd (found) for $C_5H_8N_2O$ (%): C, 53.56 (53.19); H, 7.19 (7.10); N, 24.98 (24.52).

## 1.2 Synthesis of oxidovanadium(IV) complexes

### 1.2.1 [VO(Im4COO)$_2$]

To a stirred aqueous solution of Im4COOH (0.254 g, 2.26 mmol) in water (10 mL), was added 10% $N(ME)_4Cl$ (1.04 mL, 1.13 mmol). To this solution was added aqueous $VOCl_2$ (1.13 mmol), prepared by the reaction of $VOSO_4$ with $BaCl_2$. The reaction was allowed to stir overnight, following which the light blue precipitate was collected, washed with methanol and ether and dried in an oven at 100 °C. Yield: 64.6%. Mp > 300ºC. *Anal.* Calcd (Found) for $C_8H_6N_4O_5V.H_2O$ (%): C, 31.29 (30.86); H, 2.63 (2.25); N, 18.24 (18.03). UV/Vis ($H_2O$) $\lambda_{max}$ (ε, $M^{-1}cm^{-1}$): 746 (25), 561 (10), 310sh (194). UV/Vis (solid reflectance), $\lambda_{max}$ (nm): 712, 570, 339. ATR- IR ($cm^{-1}$, neat): 987 ν(V=O); 1650 ν(C=N); 500 ν(V=N), 300-400 ν(V=N) and ν(V=O).

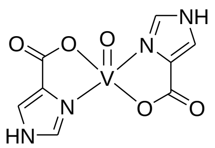

### 1.2.2 [VO(Im2COO)$_2$]

This complex was prepared in the same manner as [VO(Im4COO)$_2$], except that an aqueous solution of $VOSO_4$ was added to the ligand solution instead of aqueous $VOCl_2$. Yield: 69.9%. Mp > 300ºC. *Anal.* Calcd (Found) for $C_8H_6N_4O_5V.H_2O$ (%): C, 31.29 (30.53); H, 2.63 (2.85); N, 18.24 (17.75). UV/Vis ($H_2O$) $\lambda_{max}$ (ε, $M^{-1}cm^{-1}$): 749 (23), 550 (10), 317sh (191). UV/Vis (solid reflectance), $\lambda_{max}$ (nm): 715, 559, 333. ATR- IR ($cm^{-1}$, neat): 987 ν(V=O); 1650 ν(C=N); 500 ν(V=N), 300-400 ν(V=N) and ν(V=O).

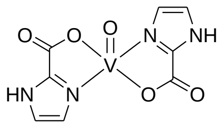

### 1.2.3 [VO(MeIm2COO)$_2$]

This complex was prepared similarly to [VO(Im4COO)$_2$]. However, upon stirring overnight a blue precipitate did not form. The blue solution was then allowed to stand for 24 hours and a light blue precipitate formed, which was filtered and washed with methanol and then ether and dried in an oven at 100 °C. Yield: 62.3%. Mp = 218-220ºC. ATR-IR ($cm^{-1}$, neat): 3134 (m), 1628(s), 1487(s), 1426(s), 1327(s), 1286(m), 1180(s), 1166(s), 963(s), 837(m), 797(s), 769(s), 698(s) $cm^{-1}$. *Anal.* Calcd (Found) for $C_{10}H_{10}N_4O_5V.H_2O$ (%): C, 35.83 (35.65); H, 3.61 (3.567); N, 16.72 (16.67)%. UV/Vis ($H_2O$) $\lambda_{max}$ (ε, $M^{-1}cm^{-1}$): 761 (23), 576 (10). UV/Vis (solid reflectance), $\lambda_{max}$ (nm): 718, 578, 352. ATR- IR ($cm^{-1}$, neat): 987 ν(V=O); 1650 ν(C=N); 500 ν(V=N), 300-400 ν(V=N) and ν(V=O).

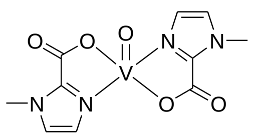

### 1.2.4 [VO(MeIm2CH$_2$OH)$_2$](PF$_6$)$_2$

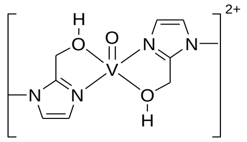

To a solution of $MeImCH_2OH$ (10 mmol) in water (1 ml) was added a solution of $VOSO_4$ (5 mmol) in water/methanol (1 ml/3 ml). This was allowed to stir for 4 hours, following which an aqueous solution of sodium hexafluoridophosphate (5 mmol) was added. An immediate light blue precipitate formed. This product was recrystallised from water/methanol (5:1) and the precipitate was washed and dried. IR ($cm^{-1}$, *Anal.* Calcd (found) for $C_{58}H_{56}B_2N_4O_3V$: C, 74.93 (77.63); H, 6.07 (6.31); N, 6.03 (6.47). UV/Vis (solid reflectance), $\lambda_{max}$ (nm): 578, 352. ATR-IR ($cm^{-1}$, neat): 1002 ν(V=O); 1650 ν(C=N); 500 ν(V=N).

## 2. Results and Discussion

In order to have a clear understanding of various species existing over the biological pH range, the stability constants approach for chemical speciation was employed.

### 2.1 Potentiometry and HYPERQUAD

#### 2.1.1 Stability constants of oxidovanadium(IV) with imidazolyl-carboxylate ligands

Previous researchers [1] in our group computed the stability constants for the interaction of these ligands (imidazole-carboxylic acids) and oxidovanadium(IV) ion. The results showed that the overall stability constant for $V^{IV}O$-1-methylimidazole-2-carboxylic acid system is far larger than other ligands such as imidazole-4-carboxylic acid and imidazole-2-carboxylic acid as shown in **Table S1**. It was explained that the high stability constant experienced with 1-methylimidazole-2-carboxylic acid (MeIm2COH) was due to the fact that the imidazole nitrogen in 1-methylimidazole-2-carboxylic acid is more basic than other nitrogen atoms in non-substituted imidazole-carboxylic acid such as imidazole-4-carboxylic acid and imidazole-2-carboxylic acid. These constants were used in this study during the refinement of binary and ternary complexes of vanadyl-ligand-small bio-ligands as well as vanadyl-ligand-high molecular weight bio-ligands systems.

**Table S1.** Protonation (log *K*) and complex formation constants (log *β*) for VO(IV)-ligand systems at 25±0.1 °C and *I* = 0.10 M (N(ME)$_4$Cl) [1].

| | **Reaction** | **Ligand** | | |
|---|---|---|---|---|
| | | **Im4COOH** | **Im2COOH** | **MeIm2COH** |
| p$K_1$ | $LH_2^+ \rightleftharpoons H^+ + LH$ | 2.70(3) | 2.72(3) | 1.30 (1) |
| p$K_2$ | $LH \rightleftharpoons H^+ + L^-$ | 6.13(1) | 6.44(2) | 6.75(3) |
| log$\beta_{110}$ | $VO^{2+} + L^- \rightleftharpoons [VO(L)]^+$ | 7.11(2) | 7.53(3) | 9.84(6) |
| log$\beta_{111}$ | $VO^{2+} + H^+ + L^- \rightleftharpoons [VO(LH)]^{2+}$ | - | - | 14.85(7) |
| log$\beta_{120}$ | $VO^{2+} + 2L^- \rightleftharpoons [VO(L)_2]$ | 11.38(8) | 11.62(6) | 15.49(9) |

The complexes formed with these ligands were considered as being ideal for application as oral agents since they would remain intact in the low pH range which prevails in the stomach up to the biological blood pH range which would allow them to be delivered intact in the blood system. This was attributed to the relative ease of coordination of the carboxylate group in the low pH range due to low p*K* value and the mild p*K* of the imidazolyl nitrogen. The species distribution diagram for the imidazole-2-carboxylate system is shown in **Figure S1** This study, therefore, explores these complexes further by considering their interaction with other biological ligands in order to gain insight in their fate *in vivo*.

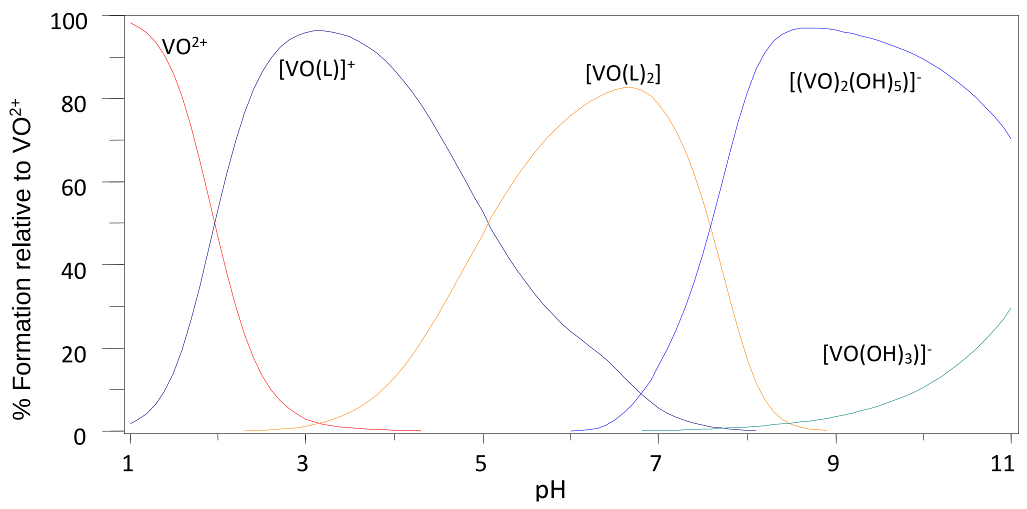


**Figure S1.** Species distribution diagram for the complexation of VO(IV) with Im2COOH (LH), $C_{VO}$ = 0.002 mol.L$^{-1}$ and $C_{ligand}$ = 0.004 mol.L$^{-1}$.

### 2.1.2 Speciation of vanadyl complexes with small bio-ligands

The bio-ligands, oxalic acid, citric acid, lactic acid and phosphate are constituents of blood serum, and because of their high affinity for hard metal ions they are the most likely low molecular mass binders of VO(IV). The binding strengths of other serum components, such as amino acids and sulfate are negligible. The protonation and overall stability constants of vanadyl-oxalate system are listed in **Table S2** together with their standard deviations and were used during the refinement of the stability constants for ternary complexes of the system $VO^{2+}$-L-Ox mentioned in the manuscript (**Table 1**).

**Table S2.** The protonation (log K) and overall stability constants of vanadyl-oxalate (log *β*) at 25°C and *I* = 0.2 molL$^{-}$ (KCl) [5].

| **Complex** | **log*K*/log*β*** |
|---|---|
| (HOx) | 3.75(2) |
| $[H_2(Ox)]$ | 1.3(2) |
| VO(Ox) | 5.77(4) |
| $[VO(Ox)_2]^{2-}$ | 10.63(2) |
| $(VO)_2(Ox)_2(OH)_2$ | 10.73(4) |
| $[VO(Ox)OH]^{-}$ | 0.44(5) |
| $[\{VO(Ox)(OH)_2]^{2-}$ | 4.02(7) |

The protonation and complexation constants of the binary VO-Oxalate system (**Table S2**) were used in the model fitting of the titration data to approximate the formation of ternary complexes. A lot of data points were available for the solution in the refinement process without termination of the calculation and the sigma values were less than 3 (as a sign of the best fitting model) (**Figure S2**).

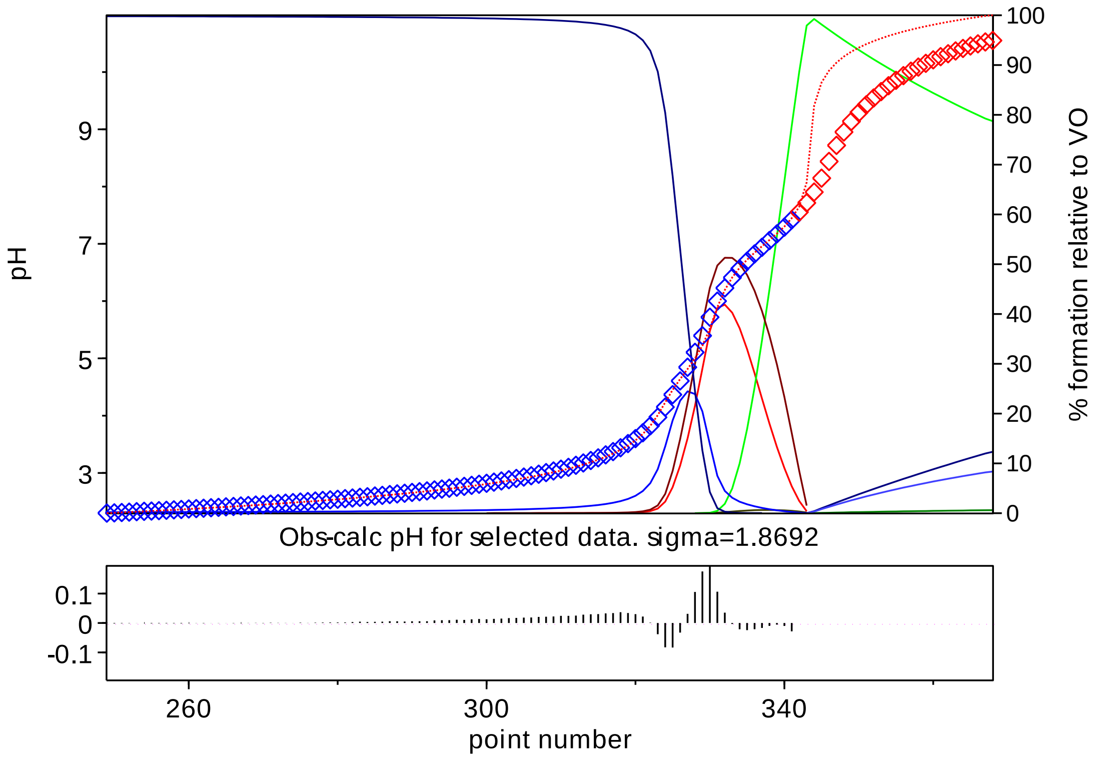


**Figure S2.** Titration and fitted curve of $VO^{2+}$-Im4COOH-oxalate system. Experimental points are represented by blue square squares and the red continuous line is the calculated line based on fitted constants. The red squares represent the experimental points that have been ignored in the refinement process. The other lines represent the species (not specified).

The formation of ternary complexes with oxalic acid was highly favoured with [VO(Ox)(Im4COO)(H)], $[VO(Ox)(Im4COO)]^{-}$ and $[VO(Ox)(Im4COO)(OH)]^{2-}$ dominating over a wide pH range (2-11). All the ligands behave the same as this system except MeIm2COOH. During the refinement of this system, the monoprotonated ternary complex (designated 1,1,1 in **Table S2**) had the formation constant of 18.90, 18.79 and 19.86 for Im4COOH, Im2COOH and MeIm2COOH, respectively. The non-protonated ternary complex (designated 1,1,0 in **Table S2**) had the constants 12.2, and 12.1 for Im4COOH and Im2COOH respectively, which are comparable to the picolinic acid system (12.38) [5]. The formation constant for MeIm2COOH as a ligand (12.89) displayed a slightly higher stability constant for the formation of this species and was comparable to maltol (mal) (13.92) and that of 6-methylpicolinic acid is much lower (11.22) [5].

**(b) Speciation of $VO^{2+}$-L-Lactate system**

The equilibrium constants for the formation of ternary species of $VO^{2+}$-L-Lactate system were also derived by best-fitting of the experimental data with a chemical model of the equilibrium system (**Figure S3**).

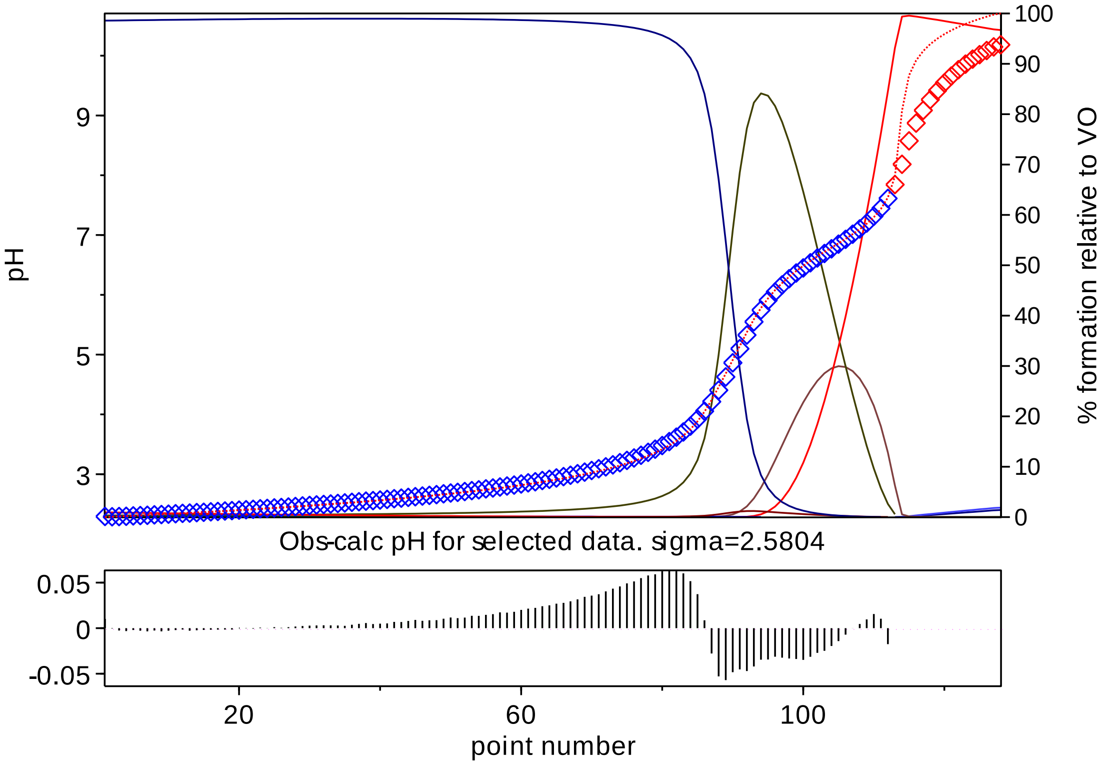


**Figure S3.** Titration and fitted curve of VO-Im2COOH-Lact system. Experimental points are represented by blue square squares and the red continuous line is the calculated line based on fitted constants. The red squares represent the experimental points that have been ignored in the refinement process. The other lines represent the species (not specified).

**(c) Speciation of $VO^{2+}$-L-Phosphate system**

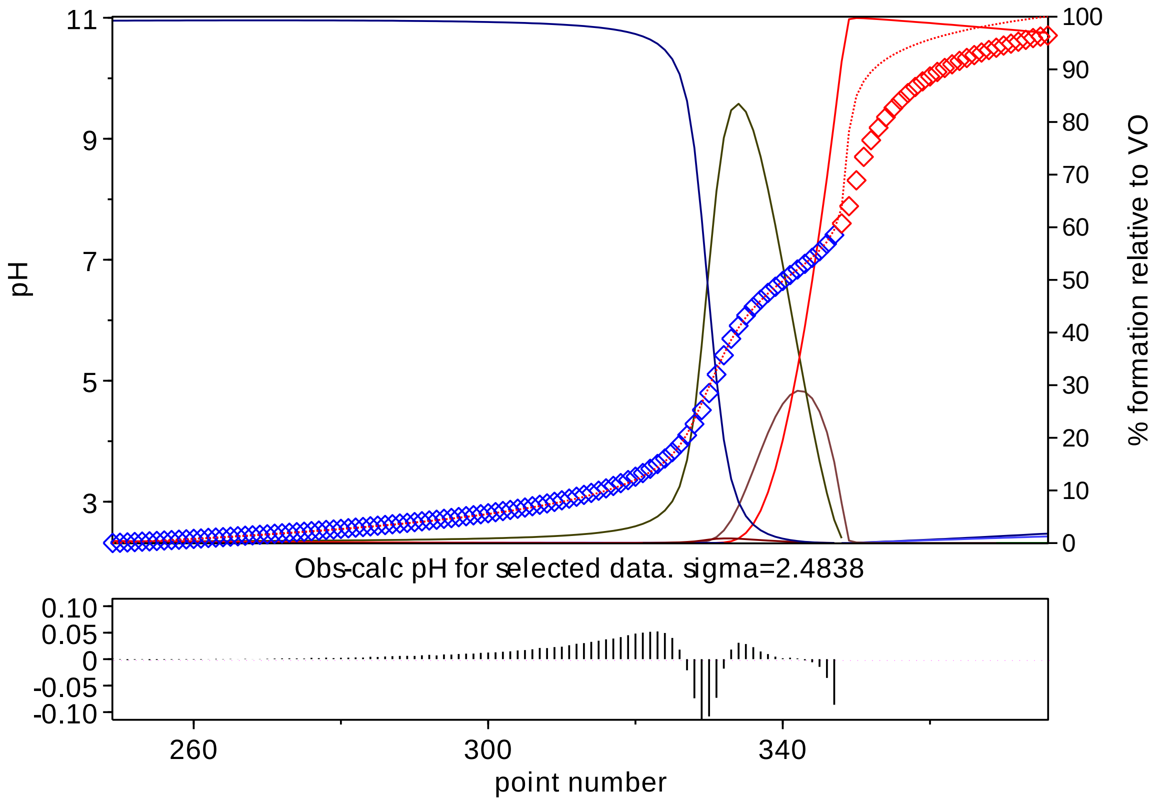


**Figure S4.** Titration and fitted curve of $VO^{2+}$-MeIm2COOH-phosphate system. Experimental points are represented by blue square squares and the red continuous line is the calculated line based on fitted constants. The red squares represent the experimental points that have been ignored in the refinement process. The other lines represent the species (not specified).

**Table S3**. Proton (log K) and Oxovanadium(IV) (log $\beta$) stability constants for the complexes of phosphate at 25ºC and $I$ = 0.20 mol.L$^{-1}$ (KCl) [6].

| **Complex** | **log*K*/log*β*** |
|---|---|
| $HPhos^{2-}$ | 11.46(0.01) |
| $H_2Phos^{-}$ | 6.63(0.01) |
| $H_3Phos$ | 1.83(0.03) |
| [VO(HPhos)] | 16.8(0.2) |
| $[VO(H_2Phos)]^{+}$ | 20.3(0.3) |
| $[VO(Phos)]^{-}$ | 10.8(0.5) |
| $[VO(Phos)(OH)_2]^{3-}$ | -3.0(0.3) |
| $[(VO)_2(Phos)_2(OH)_2]$ | 13.35(0.10) |

Phosphate complexes of VO(IV) tend to form precipitates at pH above 4 [7], however, in the presence of imidazole-carboxylic acid carrier ligands there was no precipitation and pH-metric titrations suggest the formation of ternary complexes throughout the physiological pH range studied. The species that exist at pH 7.4 are $[VOL_2]$, $[VOLPhosH]^{-}$, $[VOLPhosOH]^{3-}$ and $[VOLPhos]^{2-}$ for Im4COOH and Im2COOH. For MeIm2COOH, the species that exist at physiological pH are $[VOL_2]$, $[VOLPhosH]^{-}$ and $[VOLPhosOH]^{3-}$ and a significant quantity of $[VOL_2]$ is found at pH 7.4.

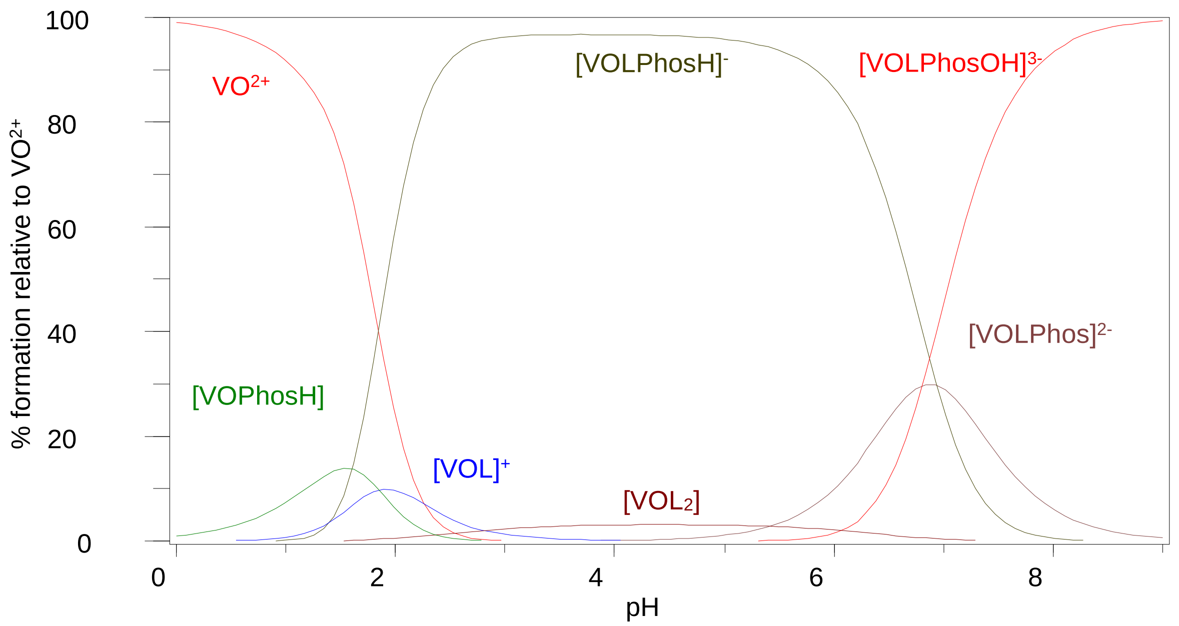


**Figure S5.** Species distribution diagram for the complexation of VO(IV) with Im4COOH (LH) and phosphoric acid (Phos), $C_{VO}$ = 0.002 mol.L$^{-1}$, VO:L:Phos (1:2:2).

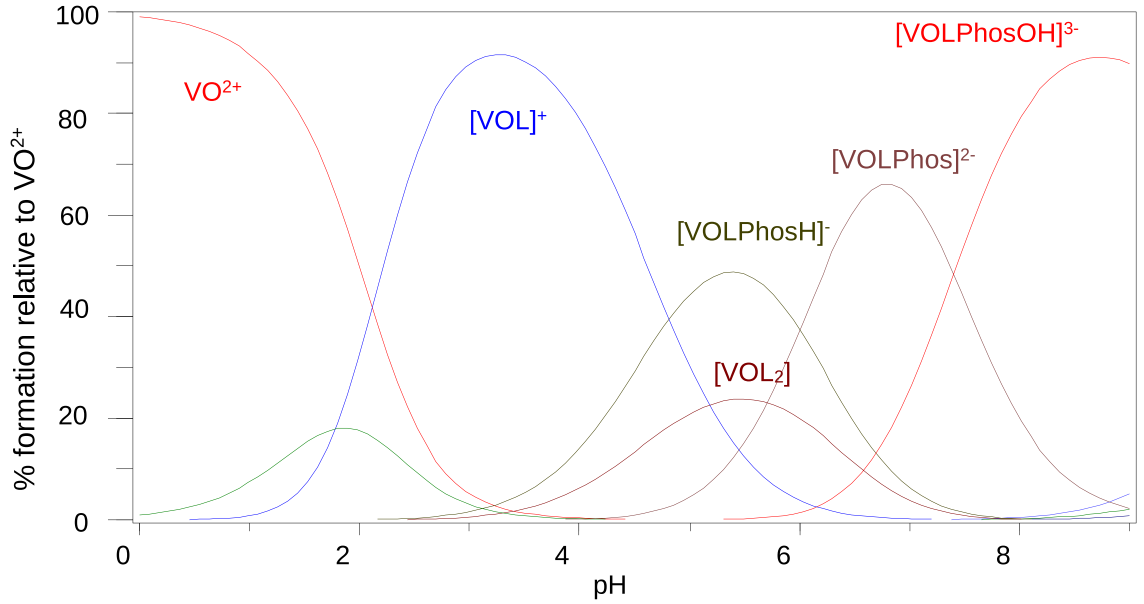


**Figure S6.** Species distribution diagram for the complexation of VO(IV) with Im2COOH (LH) and phosphoric acid (Phos), $C_{VO}$ = 0.002 mol.L$^{-1}$, VO:L:Phos (1:2:2).

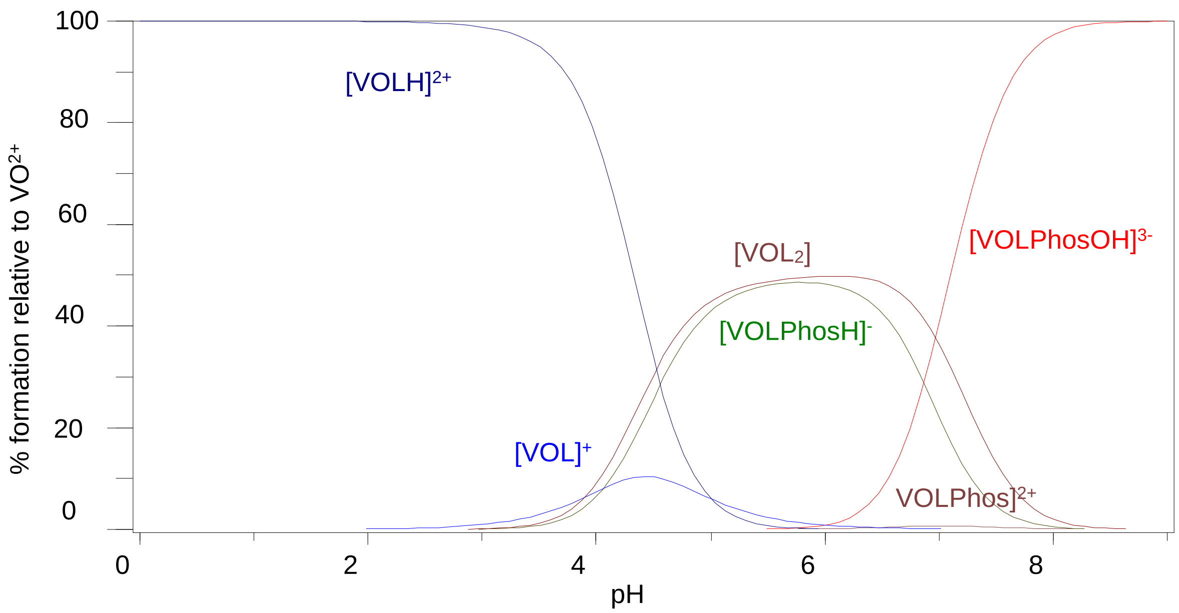


**Figure S7.** Species distribution diagram for the complexation of VO(IV) with MeIm2COOH (LH) and phosphoric acid (Phos), $C_{VO}$ = 0.002 mol.L$^{-1}$, VO:L:Phos (1:2:2).

### 2.1.3 Speciation of vanadyl complexes with proteins

#### (a) Speciation of $VO^{2+}$-L-human serum albumin (HSA) systems

The equilibrium constants for the binary and ternary complexes of vanadyl ion-ligands-HSA systems were derived by best-fitting of the experimental data with a chemical model of the equilibrium system (see **Figure S8**). The titrations were completed over a much longer period (titration rate of 0.001 ml/min) in order to obtain better titration data for this complex system. The first and second stability constants for VO-HSA and $VO(HSA)_2$ were kept constant at 9.1 and 20.9 respectively [8].

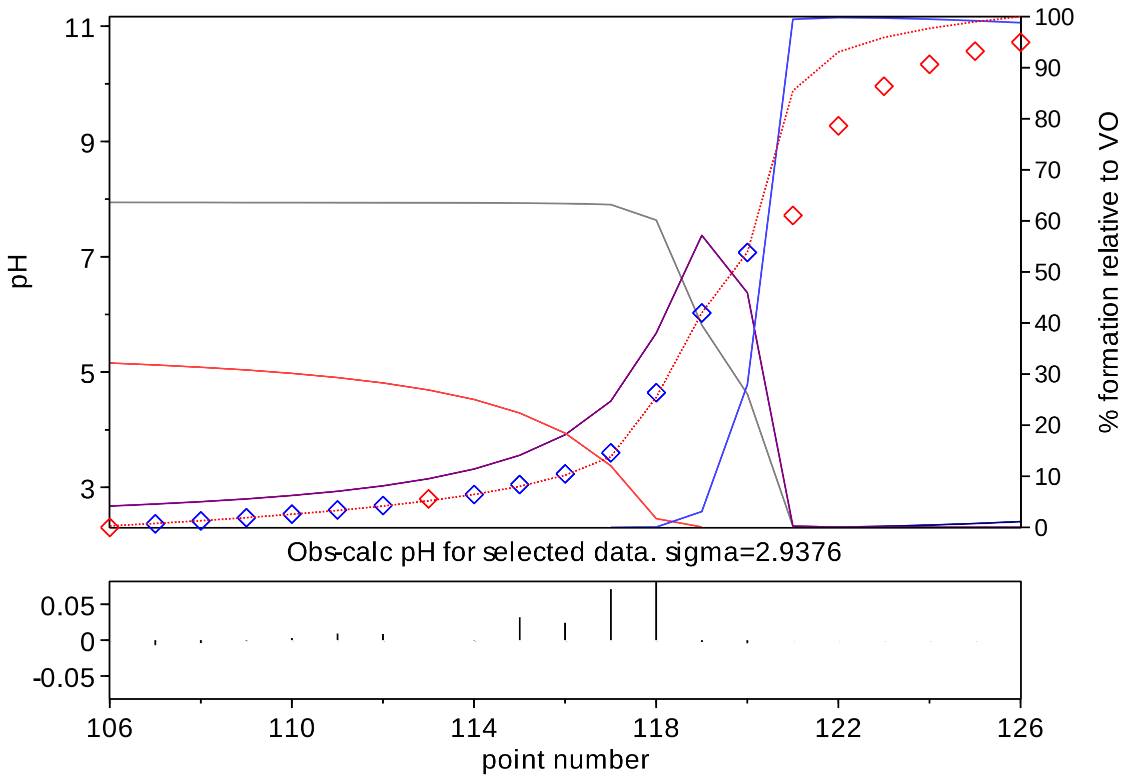


**Figure S8**. Titration and fitted curve of $VO^{2+}$-Im4COOH-HSA system. Experimental points are represented by blue squares and the calculated least-square fit by the red dotted line. The red squares represent the experimental points that have been ignored in the refinement process. The other lines represent the species (not specified).

**(b) Speciation of $VO^{2+}$-L-Human serum transferrin (hTf) systems**

The equilibrium constants for the ternary complexes of vanadyl ion-ligands-hTf systems were derived by best-fitting of the experimental titration data with a chemical model of the equilibrium system (see **Figure S9**). The titration was slowed down to get best fitting data (0.001ml/min). The following stability constants were used during modelling fitting calculations; 13.4(2) for $(V^{IV}O)$apoTf and 25.2(4) for $(V^{IV}O)_2$apoTf [8].

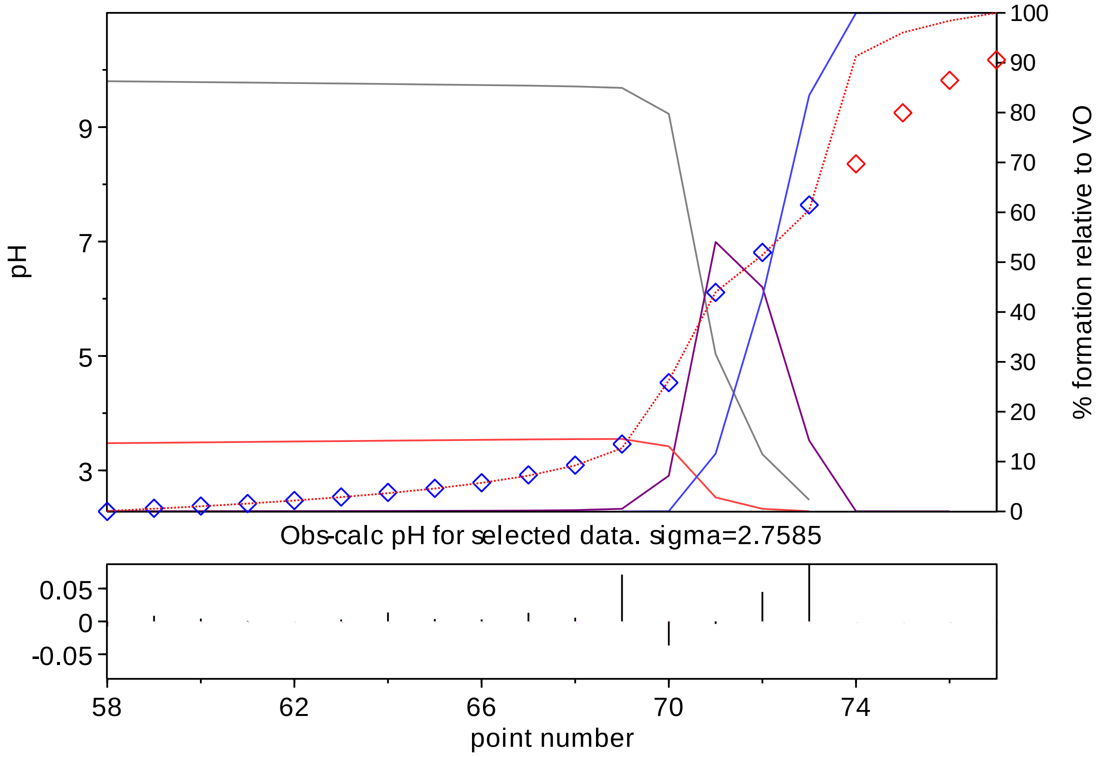


**Figure S9**. Titration and fitted curve of $VO^{2+}$-MeIm2COOH-hTf system. Experimental points are represented by blue squares and the calculated least-square fit by the red dotted line. The red squares represent the experimental points that have been ignored in the refinement process. The other lines represent the species (not specified).

This study compared the ternary stability constants of VO-L-hTf systems with other similar ligand systems in terms of structures especially those with promising anti-diabetic oxidovanadium(IV) compounds containing ligands such as pyridine-2-carboxylic acid (or picolinic acid) and maltol. The ternary stability constants designated as 2,2,1 show that the ternary complex of $VO^{2+}$, MeIm2COOH and hTf possesses a similar stability compared to the maltolato oxidovanadium(IV) complex.

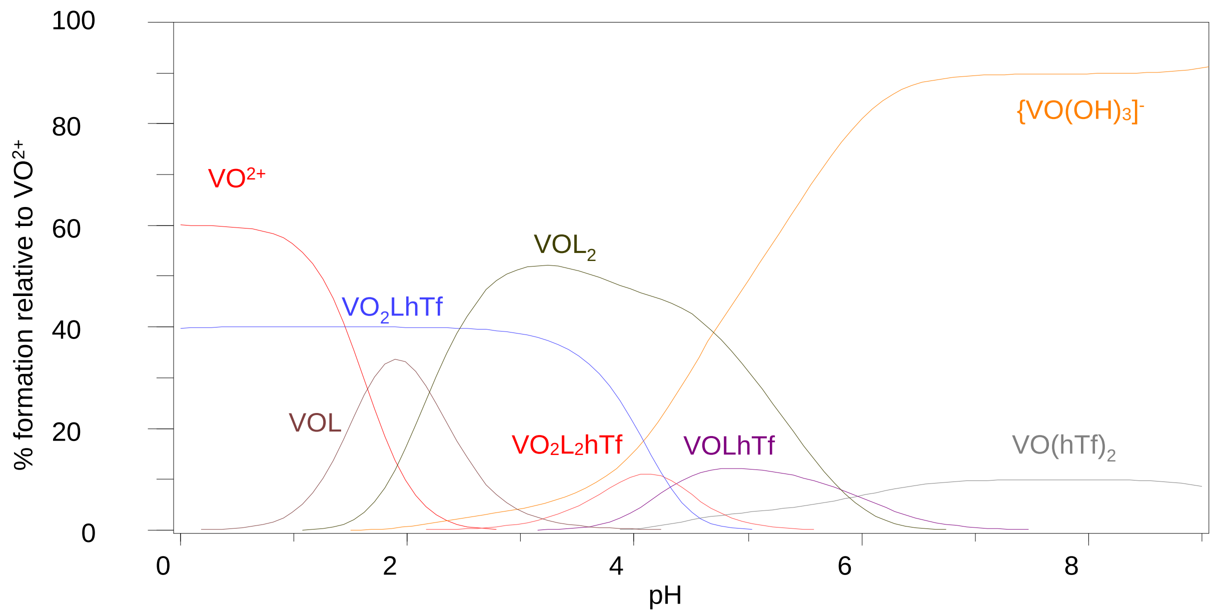


**Figure S10.** Species distribution diagram for the complexation of VO(IV) with Im4COOH (LH) and human serum transferrin (hTf), $C_{VO}$ = 0.002 mol.L$^{-1}$, VO:L:hTf (1:2:2).

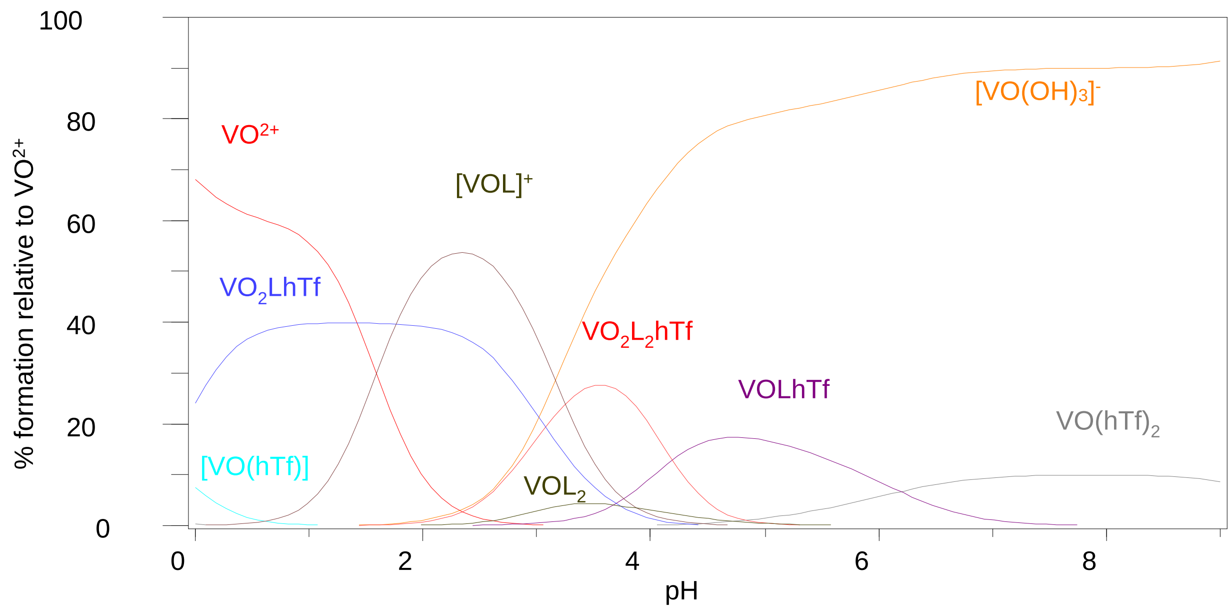


**Figure S11.** Species distribution diagram for the complexation of VO(IV) with Im2COOH (LH) and human serum transferrin (hTf), $C_{VO}$ = 0.002 mol.L$^{-1}$, VO:L:hTf (1:2:2).

### 2.2 HPLC AND LC-MS

These techniques were used to separate, identify and to confirm the species studied using potentiometry and HYPERQUAD. However, only the ternary complexes containing the small bioligands were confirmed by LC-MS (species formed at pH 7.4).

### 2.2.1 HPLC studies

#### (a) Speciation of VO-L-Phosphate systems

The HPLC method was developed such that the observed peaks could have varied retention times. There is a shoulder to the peak with a retention time is at 3.9 minutes (**Figure S12**) and it was assigned to $[VOL(H_2O)_2]^+$. The peak that appear at a retention time of 3.9 minutes was assigned to $VOL_2$ and the peak that is appearing at retention time of 4.85 minutes was assigned to $[VOPhos(H_2O)_2]^-$, where Phos is phosphate used to make phosphate buffer solution used to prepare the sample before HPLC analysis. This confirmed the observation of stability constants of phosphate which showed that it was a stronger binder compared with all the small bio-ligands investigated in this work. The peak for VO-MeIm2COOH is more intense than that VO-phosphate perhaps because MeIm2COOH is a stronger binder of the vanadyl ion compared with other imidazole-carboxylic acids as shown in the **Figure S12.**

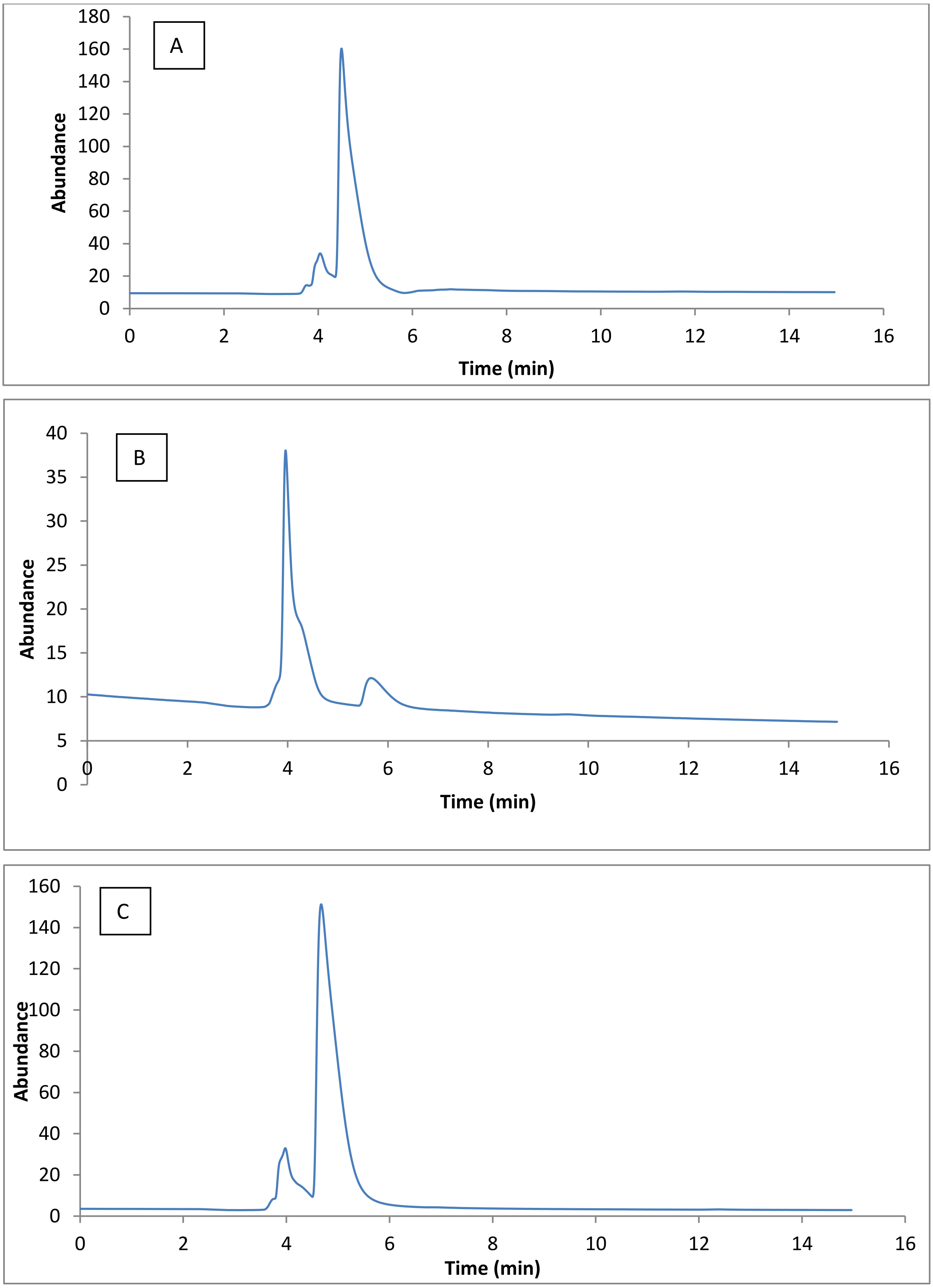


**Figure S12**. HPLC chromatograms of (A) VO(IV)-Im4COOH, (B) VO(IV)-Im2COOH, (C) VO(IV)-MeIm2COOH systems. $C_{VO} = 0.002$ mol.L$^{-1}$ and $C_L = 0.004$ mol.L$^{-1}$.

**(b) Speciation of VO-L- Cit systems**

The investigation of the interaction of oxidovadium(IV) with citric acid (Cit) and the carrier ligand in presence of a phosphate buffer using HPLC gave two broad peaks at retention times of 3.9 and 5.4 minutes as shown in **Figure S13.** The stronger peaks, identified by LC-MS, correspond to VOLCitOH and to VOLPhos, where Cit is citric acid and L represents the ligands $Im4COO^-$ and $Im2COO^-$ and $MeIm2COO^-$, respectively. The fact that these peaks are broad is due to other peaks that are masked within the broad peak (not resorved) as compared to VOLCit which is observed in species distribution diagrams derived from potentiometry [9] and LC-MS chromatograms but was not observed here.

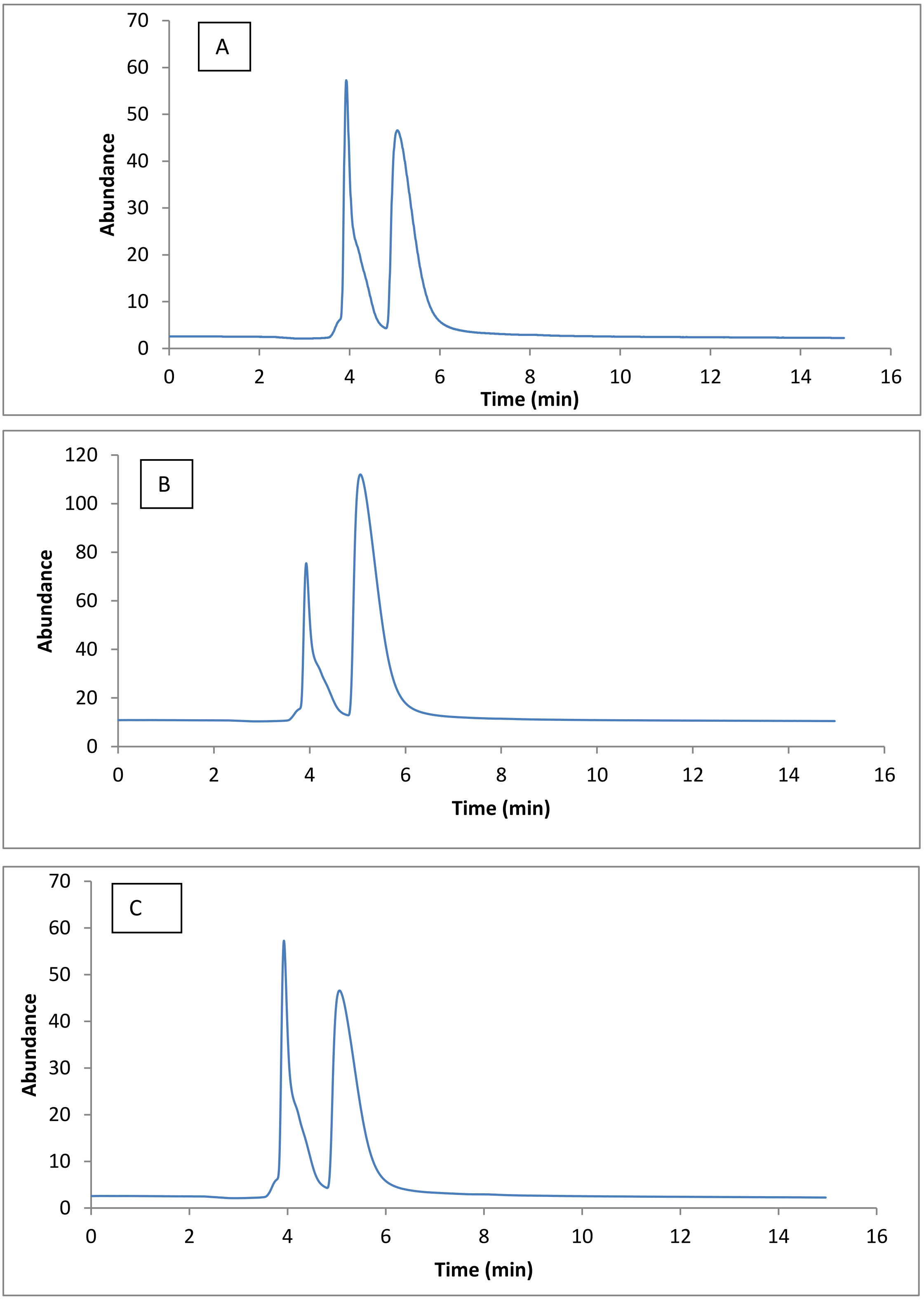


**Figure S13**. HPLC chromatograms of (A) VO(IV)-Im4COOH-Cit, (B) VO(IV)-Im2COOH-Cit, (C) VO(IV)-MeIm2COOH-Cit. $C_{VO}$ = 0.002 mol.L$^{-1}$, $C_L$ = 0.004 mol.L$^{-1}$, and $C_{Cit}$ = 0.004 mol.L$^{-1}$.

**(c) Speciation of VO-L-Ox systems**

The investigation of the interaction of oxidovadium(IV) with oxalic acid and the carrier ligands in presence of phosphate buffer using HPLC gave three sharp peaks at retention times of 3.85, 4.27 and 4.88 minutes for $Im4COO^-$ and $Im2COO^-$ and at retention times of 1.6, 2.06 and 4.16 for $MeIm2COO^-$, as shown by **Figure S14**. These strong peaks represent the intense peaks identified by LC-MS which correspond to $[VOLOX(OH)]^{2-}$, $[VOLOx]^-$ and $[VOLPhos]^{2-}$ where Ox is oxalic acid, L is a carrier ligand and Phos is the phosphate ion.

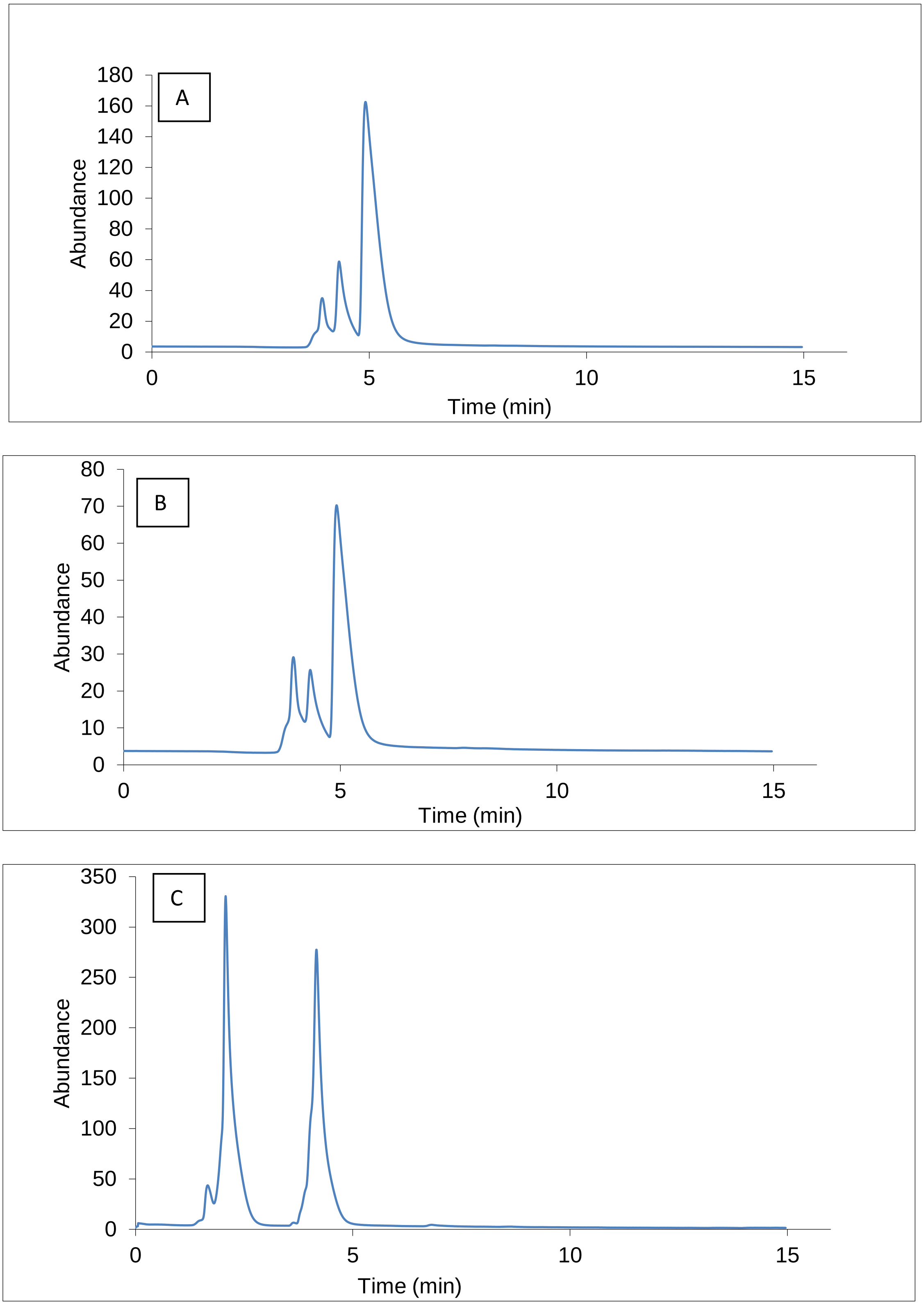


**Figure S14**. HPLC chromatograms of (A) VO(IV)-Im4COOH-Ox, (B) VO(IV)-Im2COOH-Ox, (C) VO(IV)-MeIm2COOH-Ox. $C_{VO}$ = 0.002 mol.L$^{-1}$,$C_L$ = 0.004 mol.L$^{-1}$, and $C_{Ox}$ = 0.004 mol.L$^{-1}$.

**(d) Speciation of VO-L-Lact system**

The investigation of interaction of oxidovadium(IV) with lactic acid and the carrier ligands using HPLC gave the three sharp peaks at retention times of 2.16, 2.96 and 6.97 minutes for Im4COOH and Im2COOH and 2.18, 2.70 and 6.13 for MeIm2COOH as shown in **Figure S15.** These peaks, according to LC-MS confirmation, correspond to $[VOLLact(OH)]^{-}$, VOLLact and $[VOLPhos]^{2-}$ where Lact is lactic acid, L is a carrier ligand and Phos is the phosphate ion.

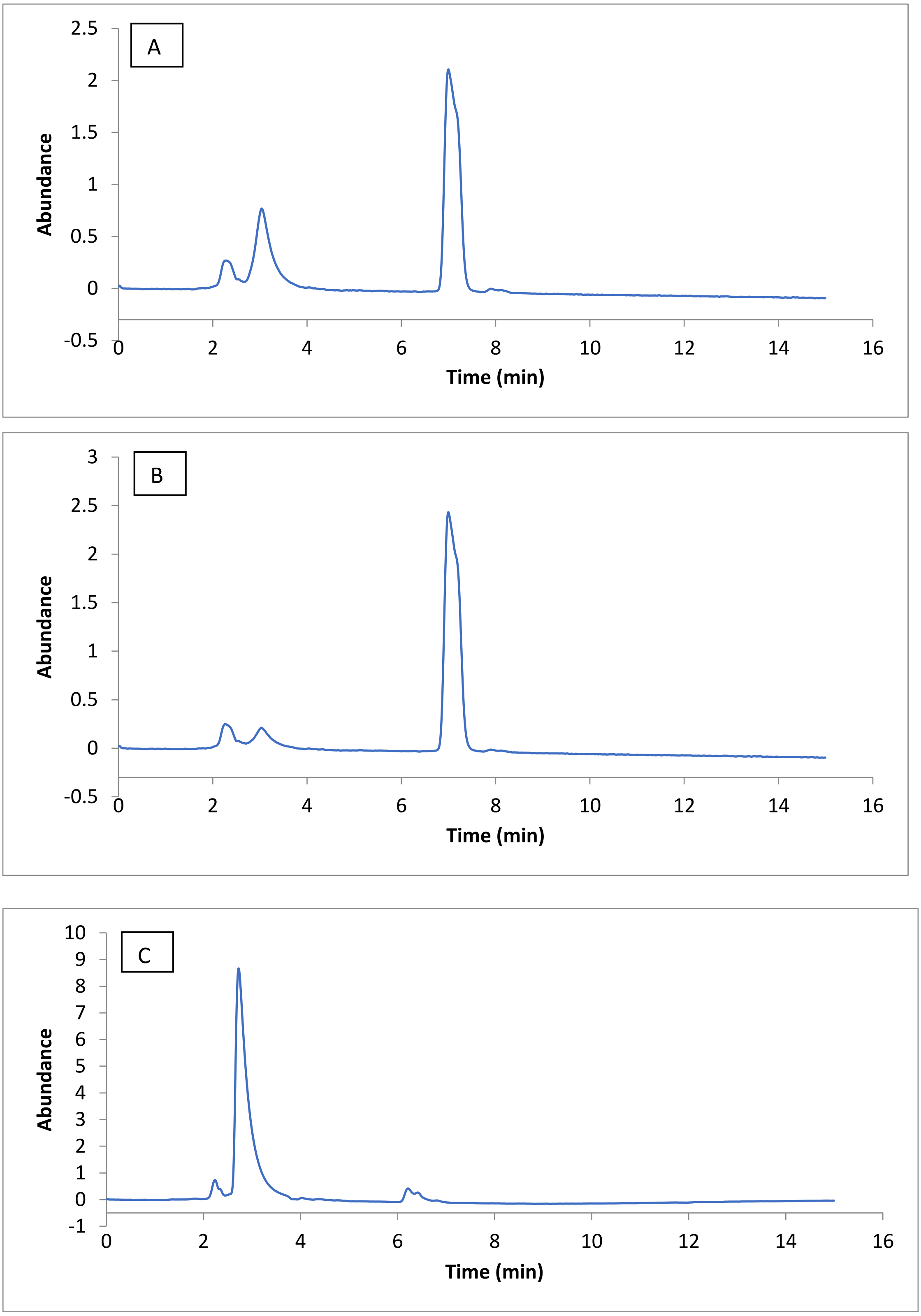


**Figure S15**. HPLC chromatograms of (A) VO(IV)-Im4COOH-Lact, (B) VO(IV)-Im2COOH-Lact, (C) VO(IV)-MeIm2COOH-Lact systems. $C_{VO}$ = 0.002 mol.L$^{-1}$,$C_{L}$ = 0.004 mol.L$^{-1}$, and $C_{Lact}$ = 0.004 mol.L$^{-1}$.

**(e) Speciation of VO-L-HSA system**

**Figure S16** represents HPLC chromatograms for (A) VO(IV)-Im4COOH-HSA, (B) VO(IV)-Im2COOH-HSA, (C) VO(IV)-MeIm2COOH-HSA respectively. The investigation of speciation of oxidovadium(IV) with human serum albumin and the carrier ligands using HPLC gave four sharp peaks at retention times of 2,18; 2.69; 6.2 and 6.3 minutes for Im4COOH and 2.21; 2.72; 6.34 and 6.44 for Im2COOH, 2.21; 6.23 and 6.44 for MeIm2COOH systems as shown in **Figure S16.** These according to MALDI-TOF-MS, correspond to $[VOPhos(H_2O)]^-$, $[VO(HSA)_2]$ and $[VOL_2]$ as well as $[VOL(HSA)_2]$ for MeIm2COOH, where HSA is human serum albumin, L is a carrier ligand and Phos is the phosphate ion.

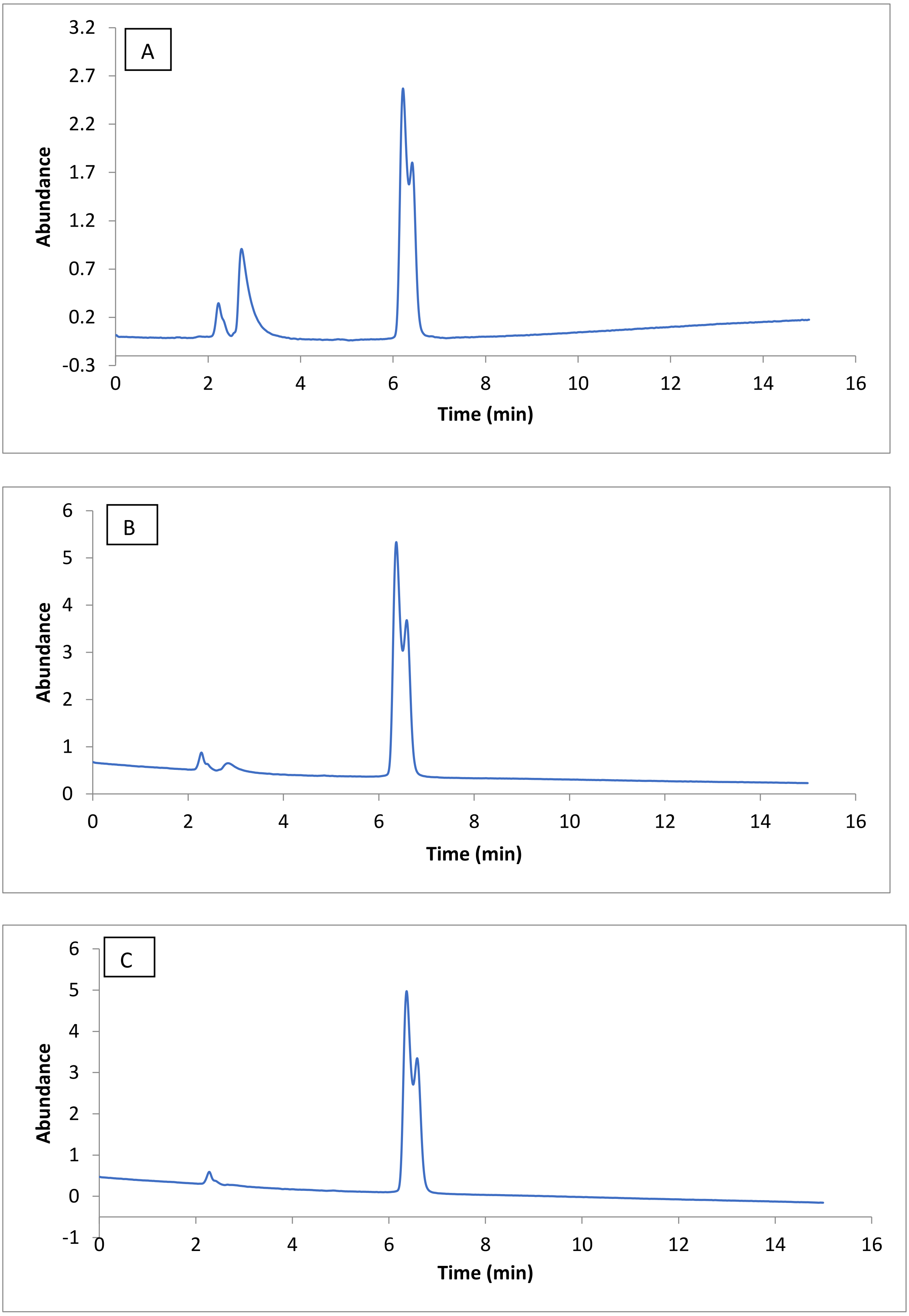


**Figure S16**. HPLC chromatograms for (A) VO(IV)-Im4COOH-HSA, (B) VO(IV)-Im2COOH-HSA, (C) VO(IV)-MeIm2COOH-HSA. $C_{VO} = 0.002$ mol.L$^{-1}$, $C_L = 0.004$ mol.L$^{-1}$, and $C_{HSA} = 0.004$ mol.L$^{-1}$.

**(f) Speciation of VO-L-hTf system**

The study of the formation of the ternary complexes of vanadium(IV) with human serum transferrin and the respective ligands using HPLC gave well separated peaks but one was particularly broad. According to MALDI-TOF-MS [**Figure S29A,B**], the peaks with retention times around 1.94, 2.68, 5.62 and 7.53 minutes correspond to [VOL(hTf)$_2$], [VO(hTf)$_2$] for Im4COOH and Im2COOH (**Figure S17**), other peak is assigned to hydrolysis specie such as VO(OH)$_3$ and the fourth one is not successfully identified. The peaks at 2.71, 3.88 and 7.69 correspond to [VOL$_2$], [VO$_2$L$_2$(hTf)] and [VOL(hTf)$_2$], for MeIm2COOH, respectively.

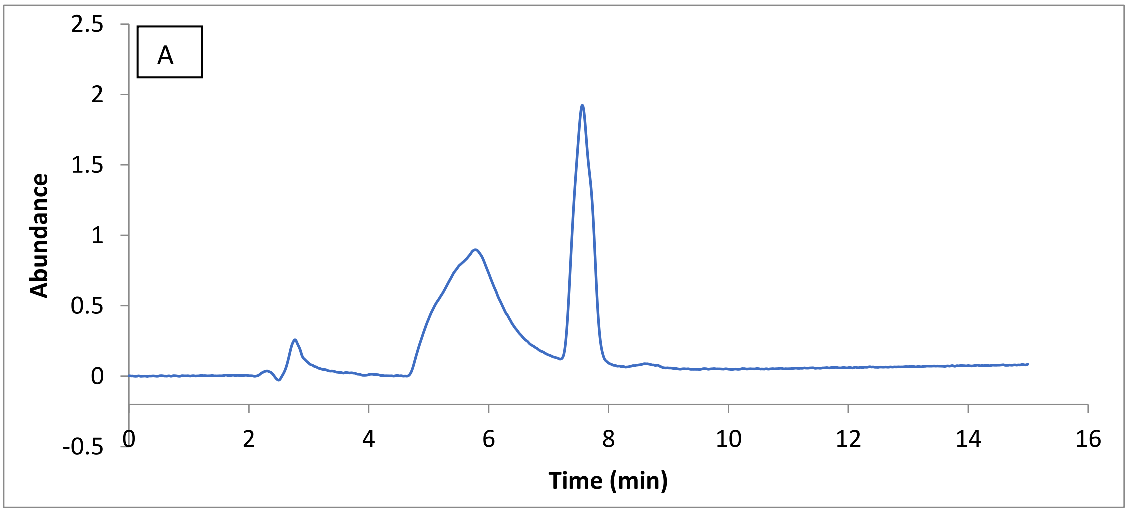

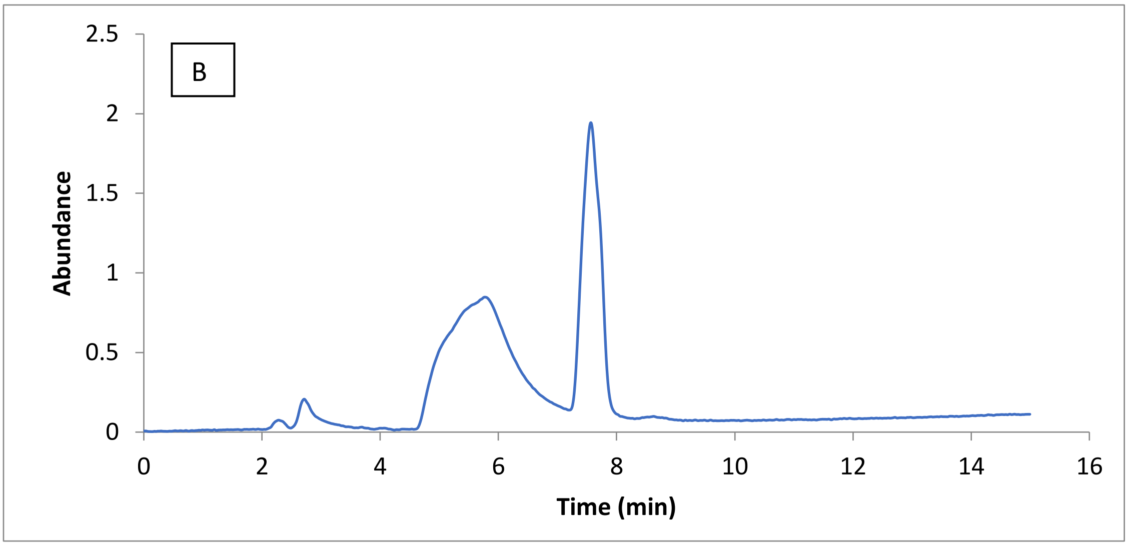


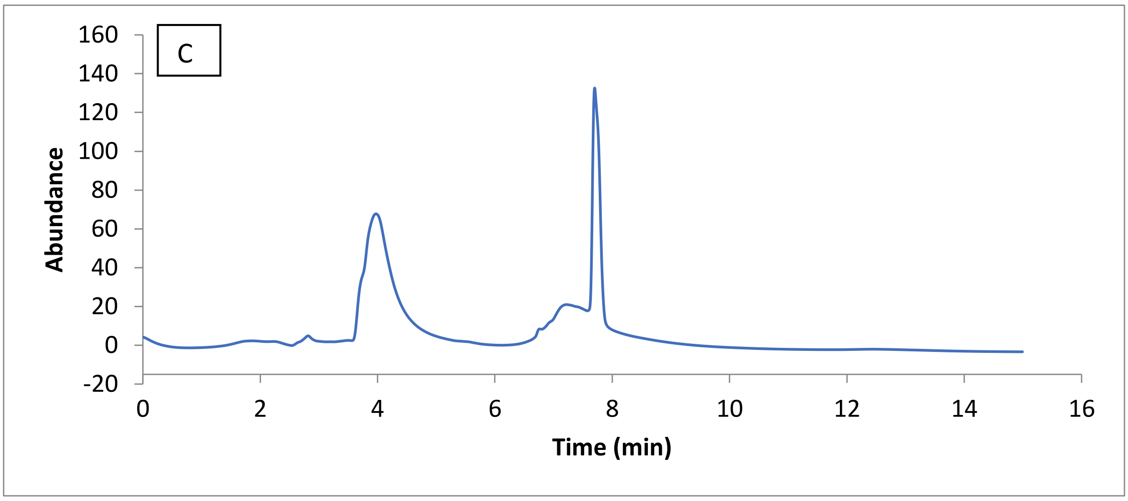


**Figure S17**. HPLC chromatograms for (A) VO(IV)-Im4COOH- hTf, (b) VO(IV)-Im2COOH- hTf and (C) VO(IV)-MeIm2COOH- hTf. $C_{VO}$ = 0.002 mol.L$^{-1}$, $C_L$ = 0.004 mol.L$^{-1}$, and $C_{hTf}$ = 0.004 mol.L$^{-1}$.

**2.2.2 LC-MS studies**

The LC-MS studies were conducted in order to complete the assignment of the HPLC peaks and some of these assignments have been briefly covered in previous section. These species also seem, to some extent, to correspond with the species that have been identified through solution chemistry modelling by potentiometry and HYPERQUAD. The reaction was carried out for 30 minutes before on LC-MS run.

**(a) Speciation of VO-L-Phosphate system**

The m/z = 289.99 corresponds to [$VOL_2$], 214.98 corresponds to [$VOL(H_2O)_2]^+$ and 279.86 was assigned to [$VO(Phos)_2(H_2O)_2]^{4-}$ (**Figures S18–S20**), where L is a ligand (Im4COOH and Im2COOH) and Phos represents the phosphate ion. For MeIm2COOH, the following species exist at pH 7.4, m/z of 226.08 corresponds to [$VOL(H_2O)_2]^+$, 279.86 was assigned to [$VO(Phos)_2(H_2O)_2]^{4-}$. These species were also identified using potentiometry and HYPERQUAD. The other peaks are related to the aqua species of vanadium(IV) as well as the phosphate species of vanadium(IV) as expected since phosphate is a strong binder of oxidovanadium(IV).

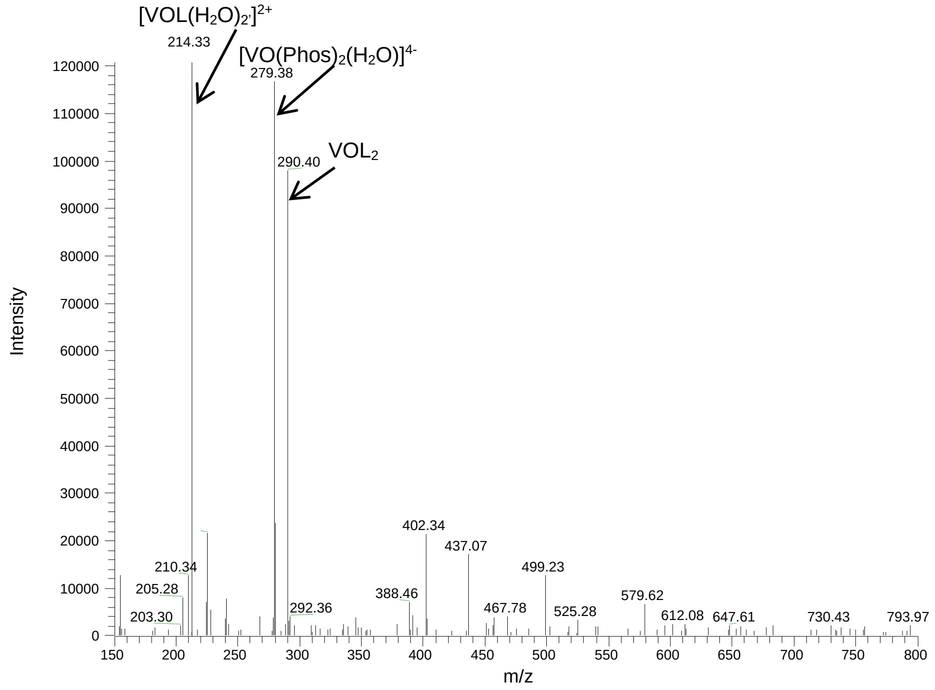


**Figure S18**. LC–MS chromatogram for $V^{IV}O$ (0.002 mol.L$^{-1}$) with Im4COOH (0.004 mol.L$^{-1}$) (A) in PBS (0.0001 mol.L$^{-1}$) (Phos) VO:L (1:2). (pH,7.4)

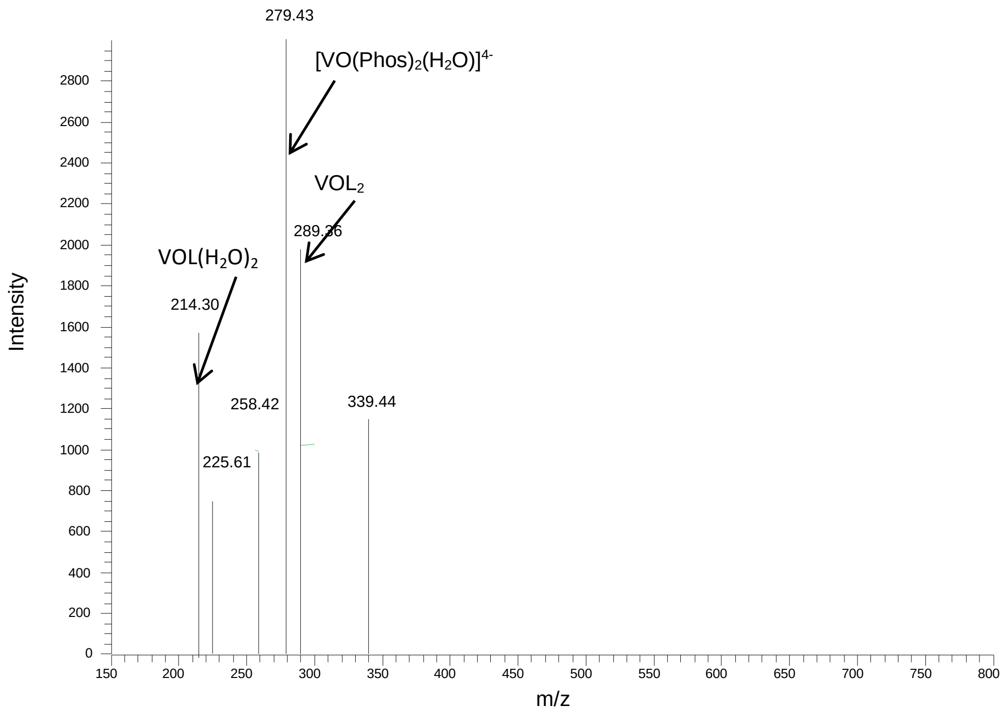


**Figure S19**. LC–MS chromatogram for $V^{IV}O$ (0.002 mol.$L^{-1}$) with Im2COOH (0.004 mol.$L^{-1}$) (A) in PBS (0.0001 mol.$L^{-1}$) (Phos). VO:L (1:2) (pH,7.4).

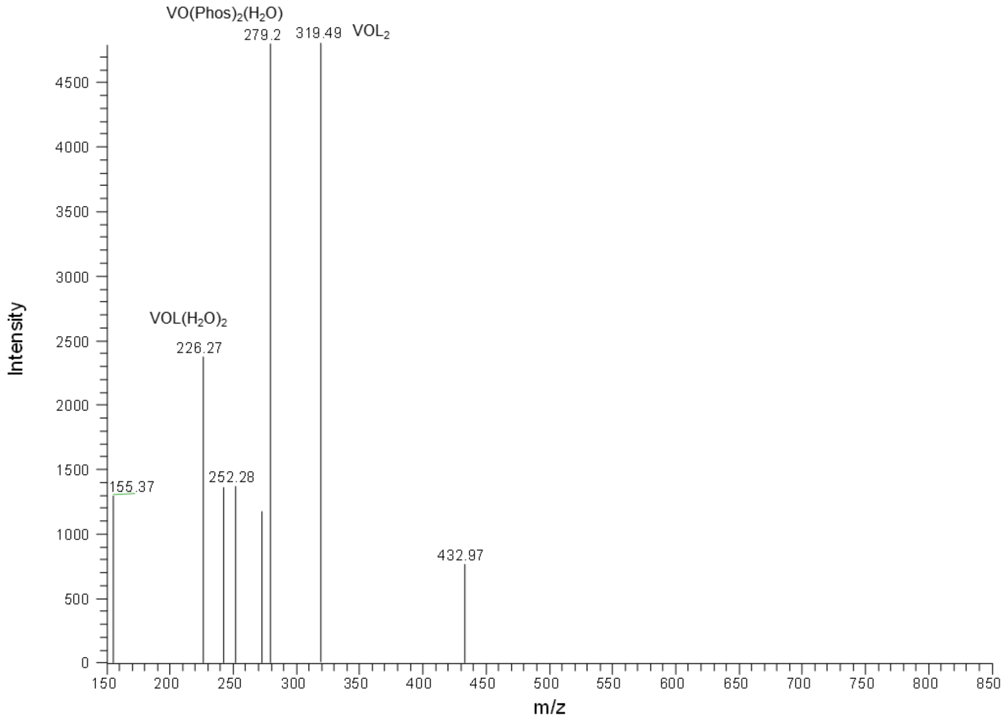


**Figure S20.** LC–MS chromatogram for $V^{IV}O$ (0.002 mol.$L^{-1}$) with MeIm2COOH (0.004 mol.$L^{-1}$) (A) in PBS (0.0001 mol.$L^{-1}$) (Phos). VO:L (1:2) (pH,7.4).

## (b) Speciation of VO-L-Cit system

The m/z of 384.76 corresponds to $[VOLCitOH]^{3-}$, 370.97 corresponds to $[VOLCit]^{2-}$, 225 matches $[VOL(H_2O)_2]^{+}$ and 290.83 was assigned to $[VOL_2]$ while 279.86 corresponds to $[VO(Phos)_2(H_2O)]^{4-}$, where Cit is citric acid, L is a ligand ($Im4COO^-$ or $Im2COO^-$), and Phos is the phosphate ion (**Figures S21 and S22**). For MeIm2COOH, the following peaks exist at pH 7.4; 380 corresponding to VOLCit and 397 is assigned to VOLCitOH as shown in **Figure S23**. These LC-MS studies confirm, in full, all the species that have been identified by potentiometry and HYPERQUAD. The other peaks are due to the aqua species of vanadium(IV) as well as the oxidovanadium(IV)-phosphate species for example the peak at 279 corresponds to $[VO(Phos)_2(H_2O)]^{4-}$, where Phos represents the phosphate ion.

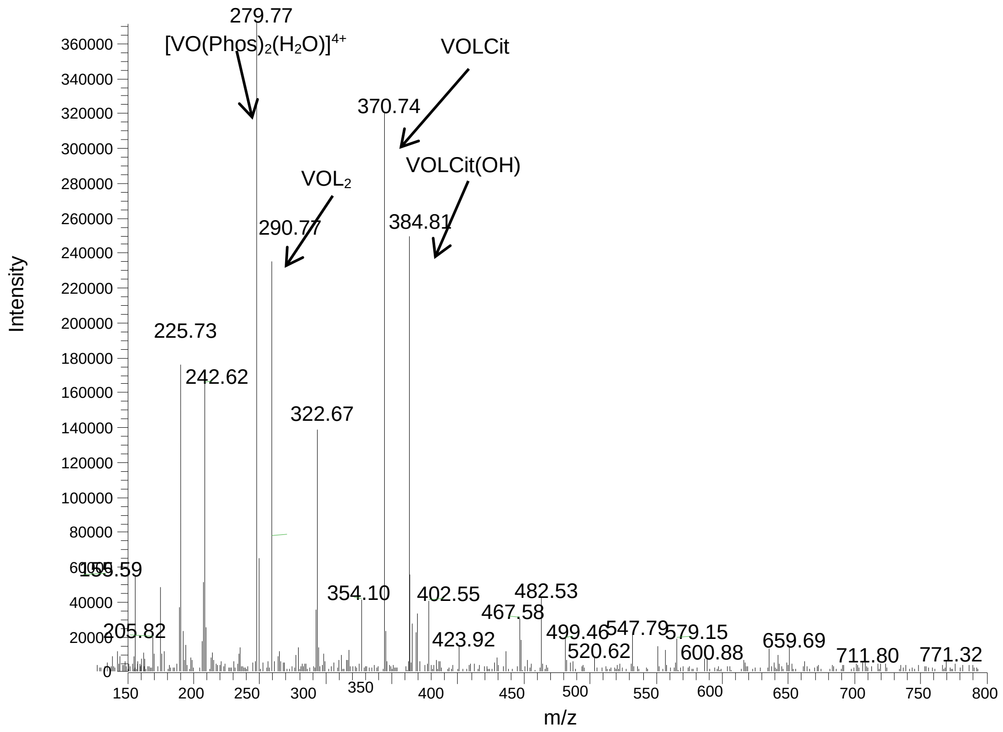


**Figure S21**. LC–MS chromatogram for $V^{IV}O$ (0.002 mol.L$^{-1}$) with Im4COOH (0.004 mol.L$^{-1}$) (L) and Citric acid in PBS (0.0001 mol.L$^{-1}$) (Phos). VO:L:Cit (1:2:2) (pH,7.4).

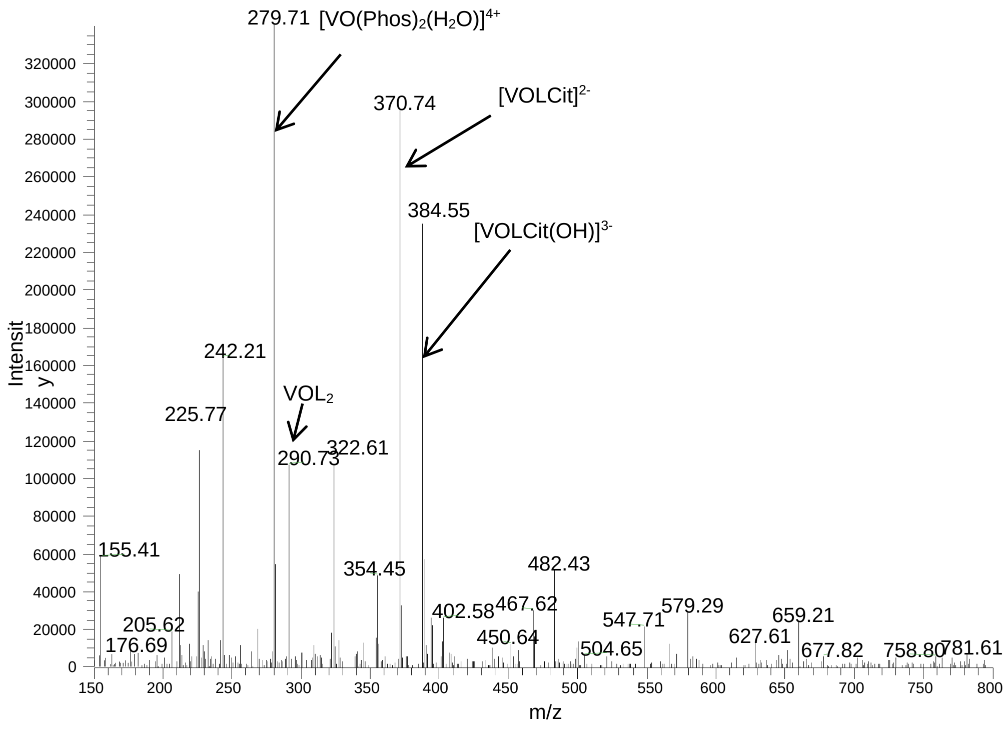


**Figure S22**. LC–MS chromatogram for $V^{IV}O$ (0.002 mol.$L^{-1}$) with Im2COOH (0.004 mol.$L^{-1}$) (L) and Citric acid in PBS (0.0001 mol.$L^{-1}$) (Phos). VO:L:Cit (1:2:2) (pH,7.4).

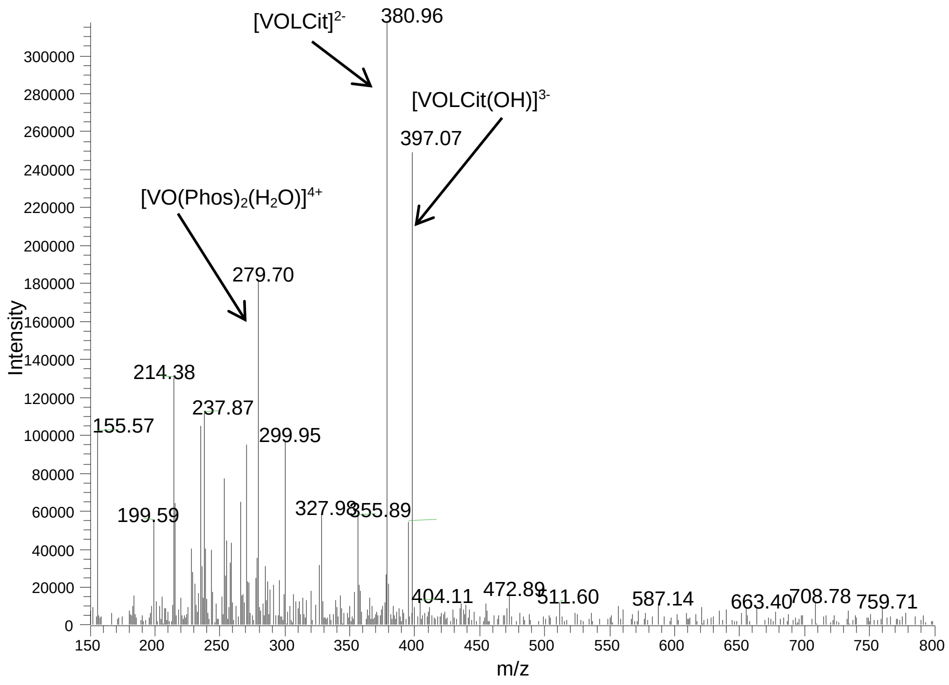


**Figure S23**. LC–MS chromatogram for $V^{IV}O$ (0.002 mol.L$^{-1}$) with MeIm2COOH (0.004 mol.L$^{-1}$) (L) and Citric acid in PBS (0.0001 mol.L$^{-1}$) (Phos). VO:L:Cit (1:2:2) (pH,7.4).

**(c) Speciation of VO-L-Ox system**

The peak at m/z = 268.99 corresponds to [VOLOx]$^{-}$ and 283.95 is assigned to [VOLOx(OH)]$^{2-}$ for Im4COO$^{-}$ and Im2COO$^{-}$ (**Figures S24** and **S25)**. The m/z = 280.97 corresponds to [VOLOx(OH)]$^{2-}$ and 320.03 corresponds to [VOL$_2$] (intense peak) for MeIm2COO$^{-}$ (**Figure S26**), and these are the same species that were identified using potentiometry and HYPERQUAD at pH 7.4. The additional peaks are related to aqua and phosphate species, and the one at m/z = 155 corresponds to [VO(H$_2$O)$_5$]$^{2+}$ while the one at m/z = 279.86 was assigned to [VO(Phos)$_2$(H$_2$O)]$^{4+}$.

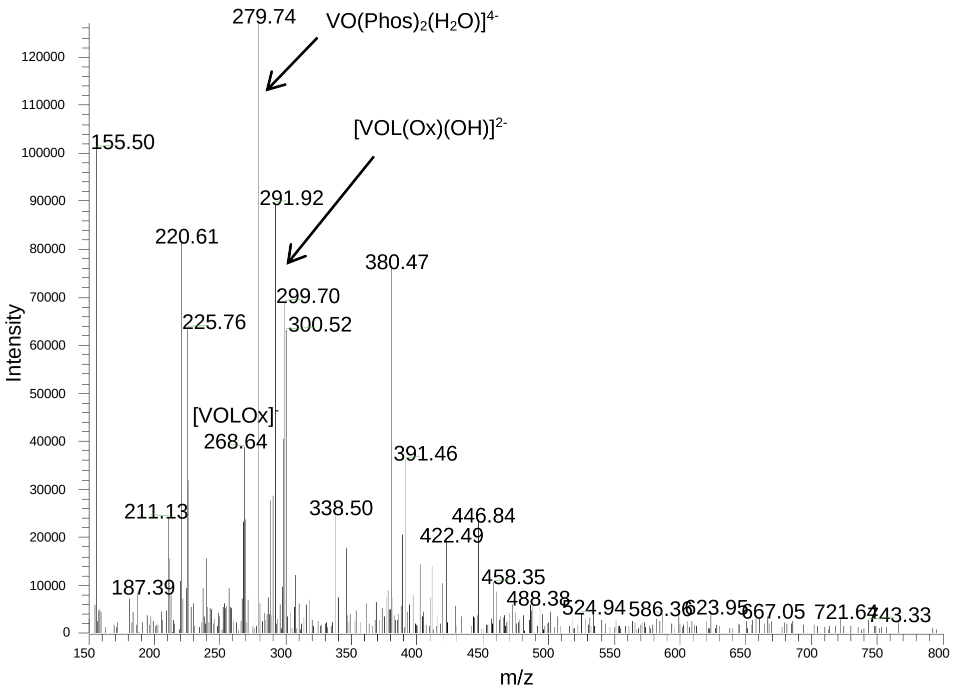


**Figure S24**. LC–MS chromatogram for $V^{IV}O$ (0.002 mol.$L^{-1}$) with Im4COOH (0.004 mol.$L^{-1}$) (L) and Oxalic acid in PBS (0.0001 mol.$L^{-1}$) (Phos). VO:L:Ox (1:2:2) (pH,7.4).

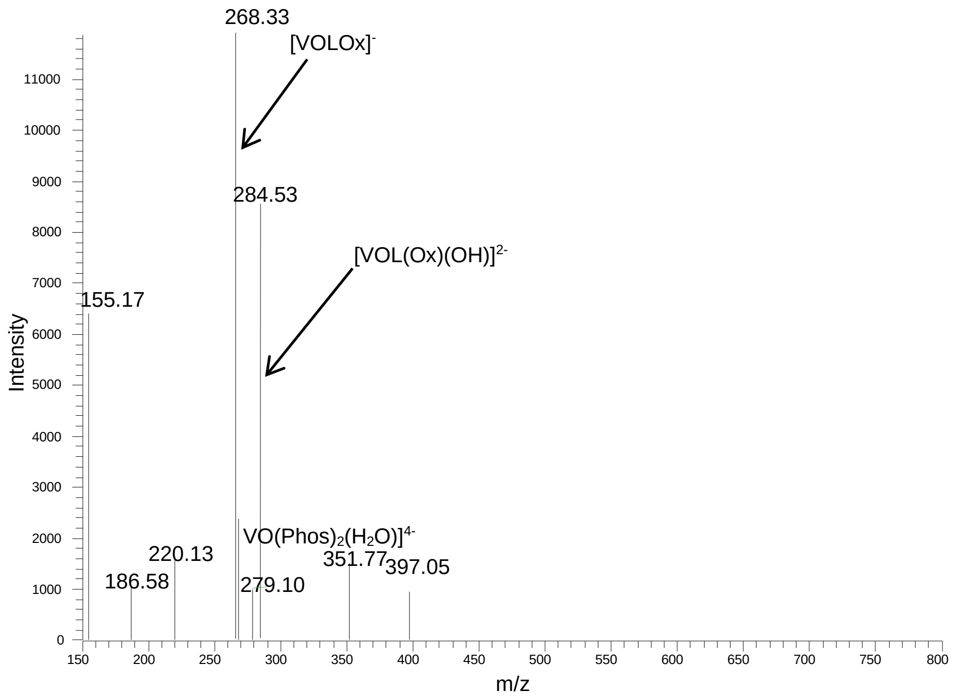


**Figure S25**. LC–MS chromatogram for $V^{IV}O$ (0.002 mol.$L^{-1}$) with Im2COOH (0.004 mol.$L^{-1}$) (L) and Oxalic acid in PBS (0.0001 mol.$L^{-1}$) (Phos) VO:L:Ox (1:2:2). (pH,7.4).

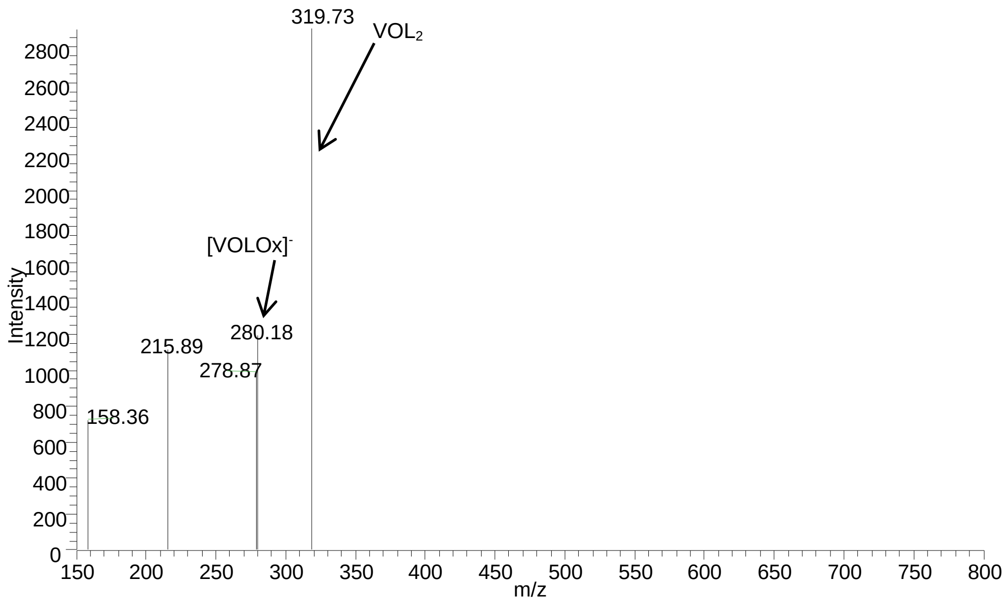


**Figure S26**. LC–MS chromatogram for $V^{IV}O$ (0.002 mol.L$^{-1}$) with MeIm2COOH (0.004 mol.L$^{-1}$) (L) and Oxalic acid in PBS (0.0001 mol.L$^{-1}$) (Phos). VO:L:Ox (1:2:2) (pH,7.4).

**(d) Speciation of VO-L-Lactic acid (Lact) system**

The peak at m/z = 267.98 was assigned to VOL(Lact) and the peak at m/z = 287.12 was assigned to $[VOL(Lact)OH]^-$ for Im4COOH and Im2COOH as shown in **Figures S27** and **S28**. The m/z = 280.11 corresponds to [VOL(Lact)]. These are the same species that were identified using potentiometry and HYPERQUAD at pH 7.4. The additional peaks are related to the aqua and phosphato species of oxidovanadium(IV), for example the one at m/z = 155 corresponds to $[VO(H_2O)_5]^{2+}$ and the one at 279.03 was assigned to $[VO(Phos)_2(H_2O)]^{4-}$. Species for MeIm2COOH are presented in **Figure S29**.

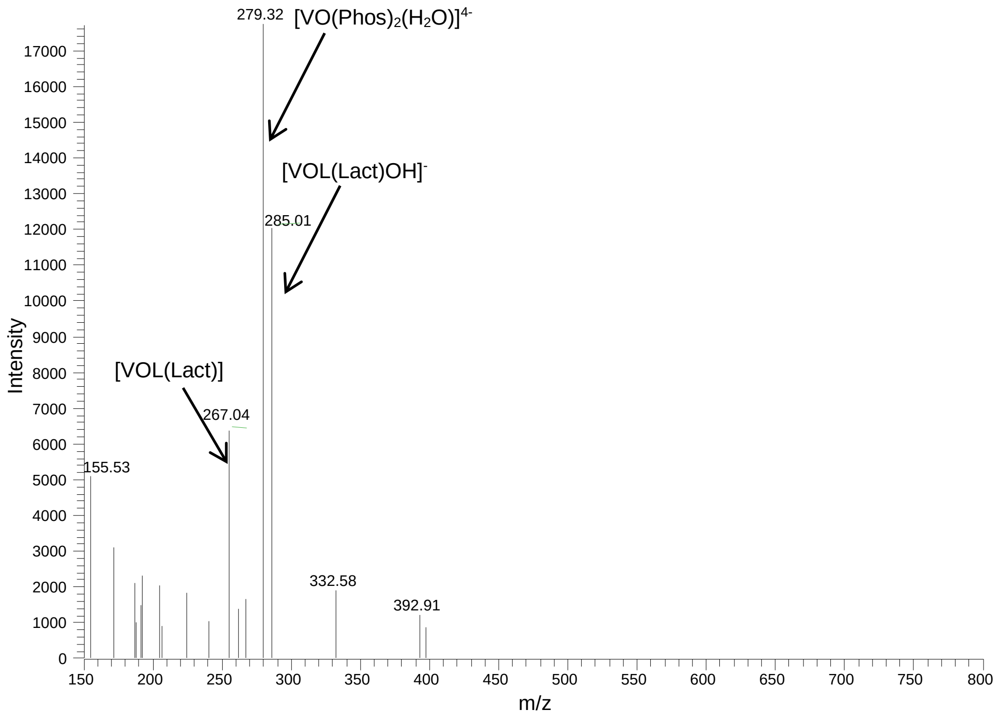


**Figure S27**. LC–MS chromatogram for $V^{IV}O$ (0.002 mol.$L^{-1}$) with Im4COOH (0.004 mol.$L^{-1}$) (L) and Lactic acid in PBS (0.0001 mol.$L^{-1}$) (Phos) VO:L (1:2:2) (pH,7.4).

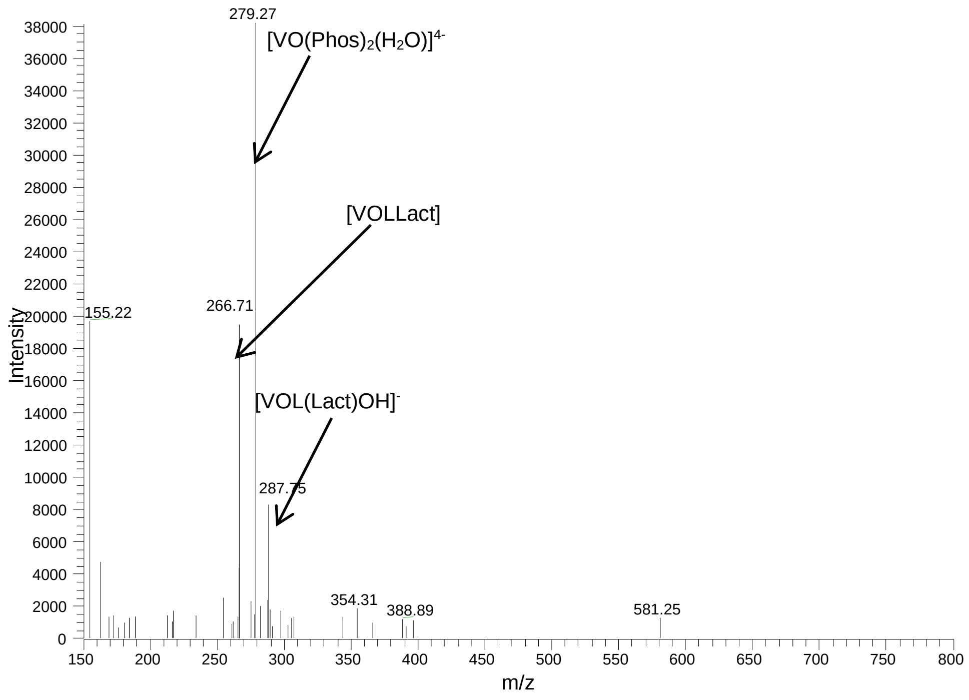


**Figure S28**. LC–MS chromatogram for $V^{IV}O$ (0.002 mol.$L^{-1}$) with Im2COOH (0.004 mol.$L^{-1}$) (L) and Lactic acid in PBS (0.0001 mol.$L^{-1}$) (Phos). VO:L (1:2:2) (pH,7.4).

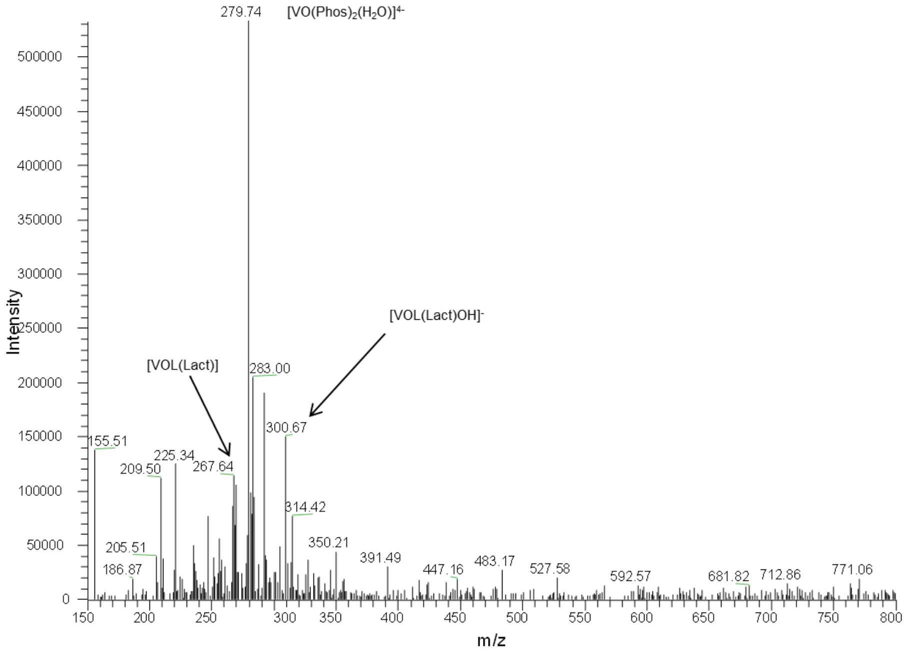


**Figure S29.** LC–MS chromatogram for $V^{IV}O$ (0.002 mol.$L^{-1}$) with MeIm2COOH (0.004 mol.$L^{-1}$) (L) and Lactic acid in PBS (0.0001 mol.$L^{-1}$). (Phos) VO:L (1:2:2) (pH,7.4).

### 2.2.3 Speciation of vanadyl-L-HMM system using MALDI-TOF-MS

### (a) Speciation of vanadyl-L-Human serum albumin (HSA) system

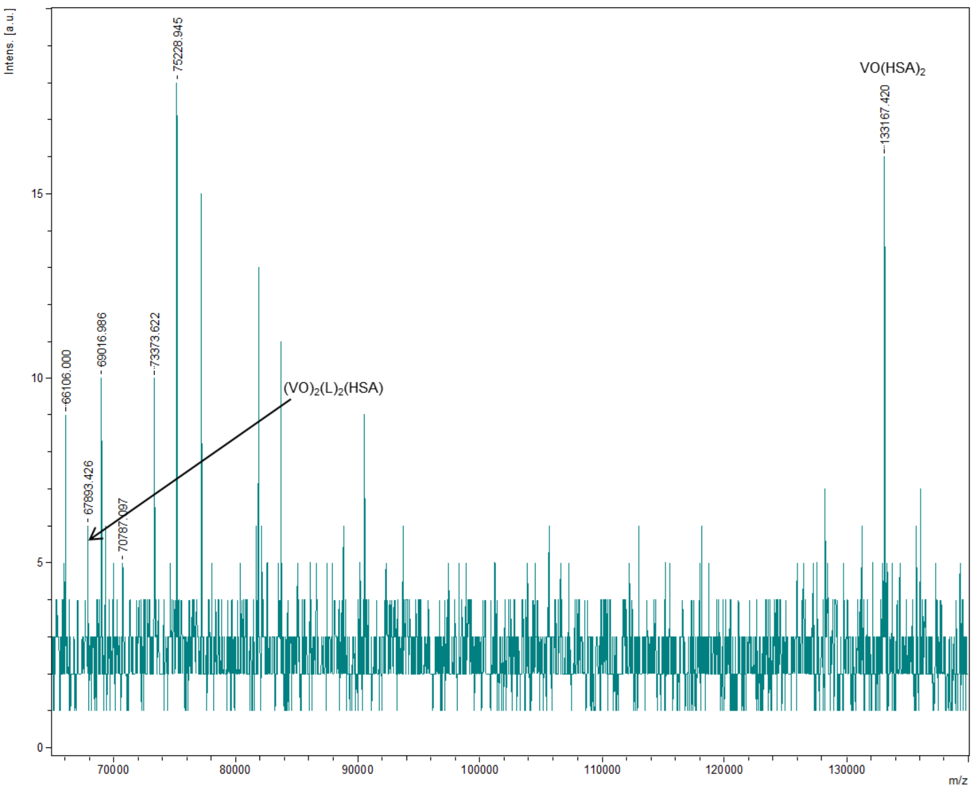


**Figure S30.** MALDI spectra of oxidovanadium(IV)-L with human serum albumin (L = Im4COOH).

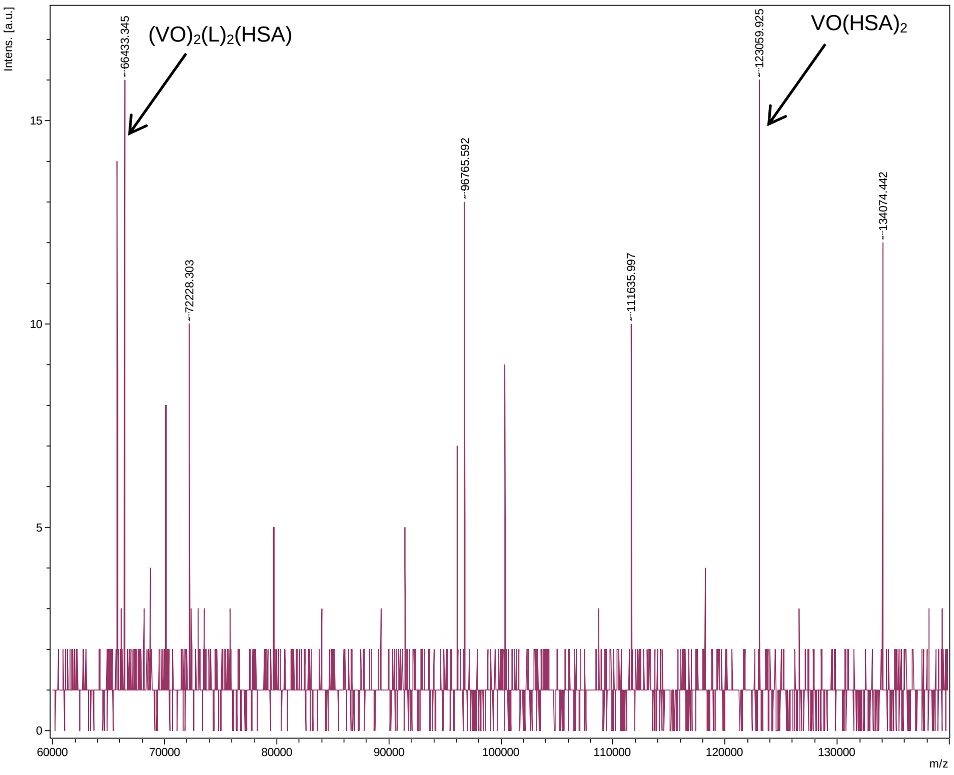


**Figure S31.** MALDI spectra of oxidovanadium(IV)-L with human serum albumin (L = Im2COOH).

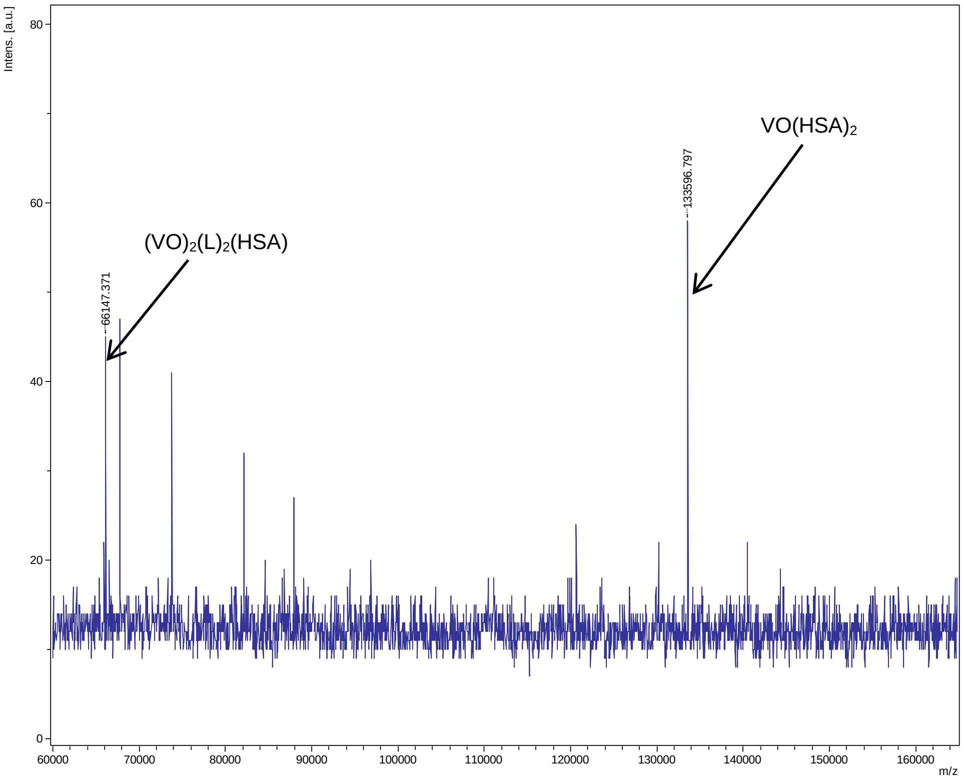


**Figure S32.** MALDI spectra of oxidovanadium(IV)-L with human serum albumin (L = MeIm2COOH).

## (b) Speciation of vanadyl-L-Human serum transferrin (hTf) system

**Figures S33–S35** illustrates the MALDI spectra of VO-L-hTf systems. The peak at 80000 amu corresponds to $(VO)_2(L)_2(hTf)$ for all ligand systems, and the one around 160000 amu corresponds to $VO(hTf)_2$. Other peaks are due to the fragmentation of the proteins and were not identified. These results are in agreement with the species modelled by potentiometry and HYPERQUAD as shown in species distribution diagrams at physiological pH 7.4.

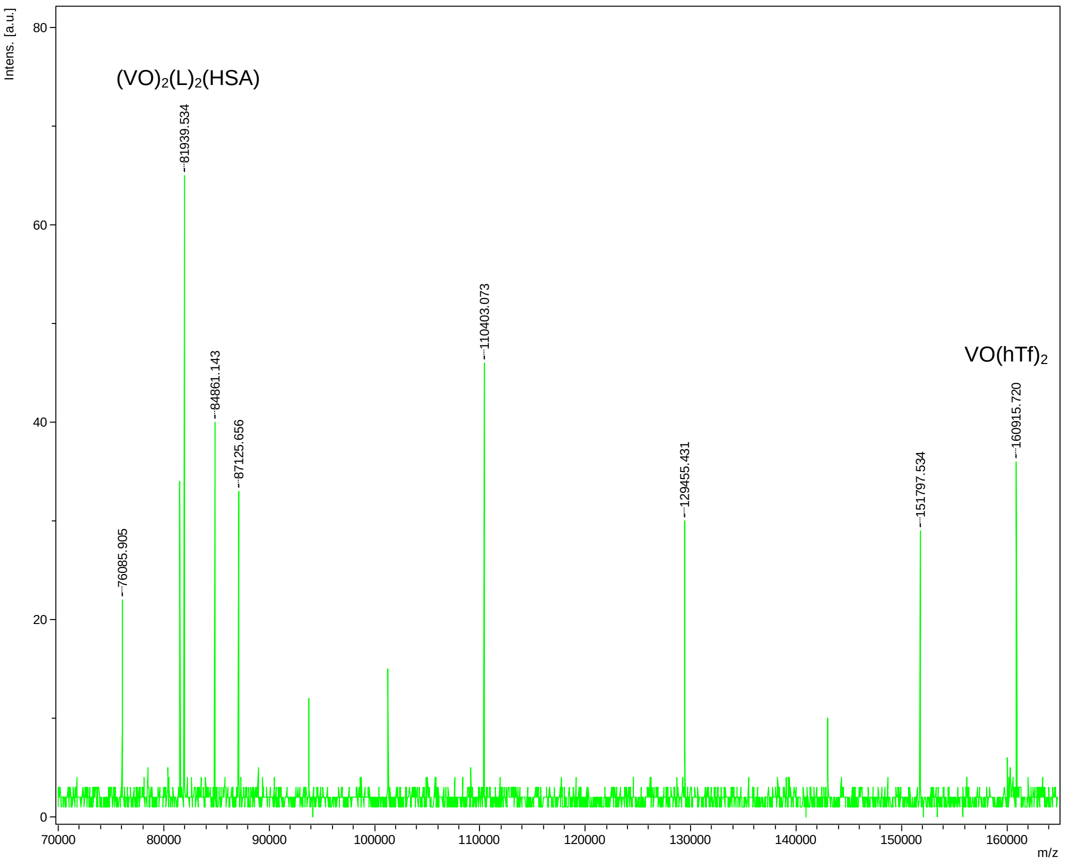


**Figure S33.** MALDI spectra of oxidovanadium(IV)-L with human serum transferrin (L = Im4COOH). VO:L:hTf (1:2:2) (pH,7.4).

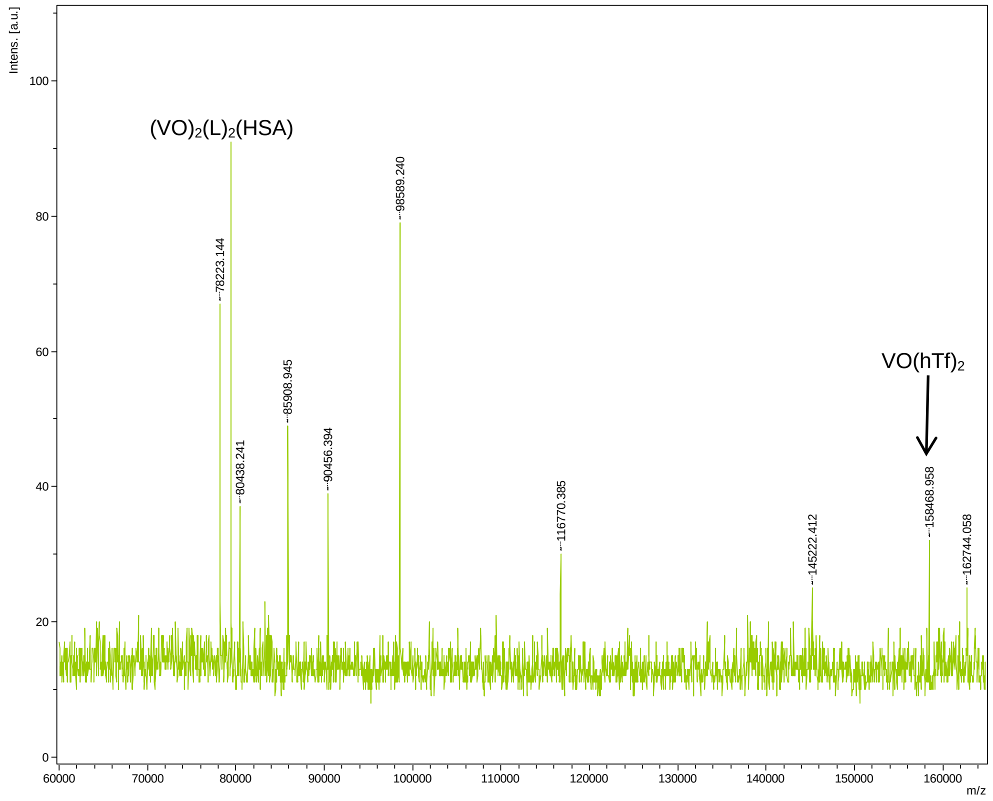


**Figure S34.** MALDI spectra of oxidovanadium(IV)-L with human serum transferrin (L = Im2COOH). VO:L:hTf (1:2:2) (pH,7.4).

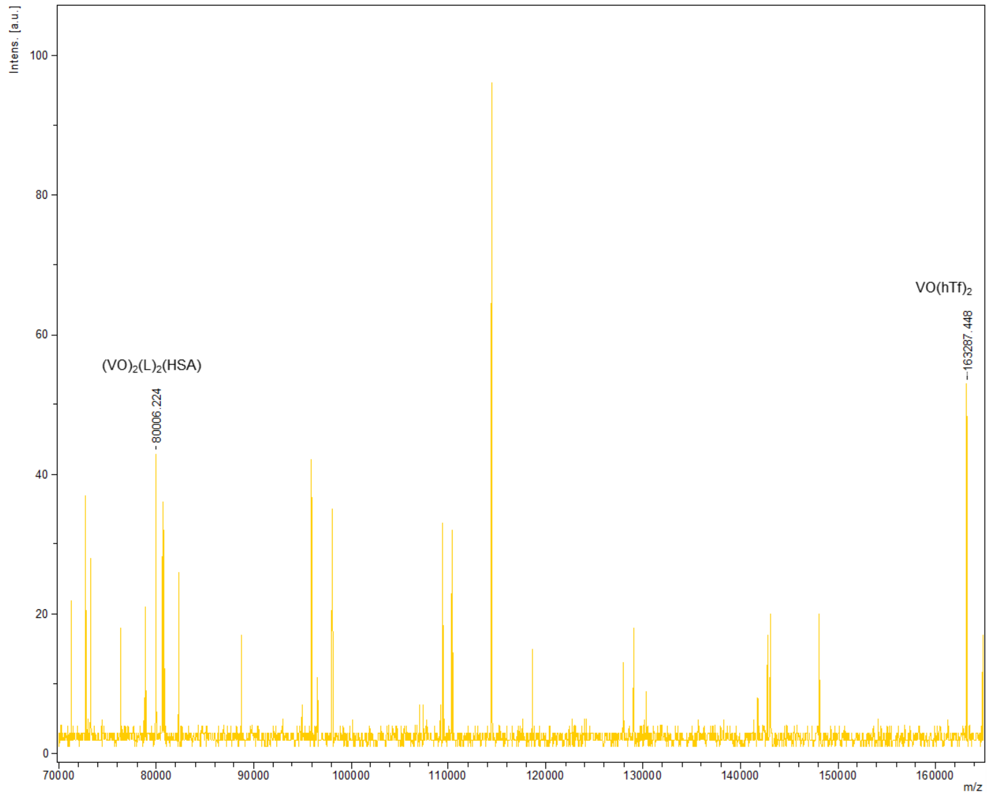


**Figure S35**. MALDI spectra of oxidovanadium(IV)-L with human serum transferrin (L = MeIm2COOH), VO:L:hTf (1:2:2) (pH,7.4).

### 3.3.3 Speciation of vanadyl-L-bioligands using EPR

It is a technique for studying materials with unpaired electrons and radicals. Unpaired electrons change their spin state by absorbing microwave energy in the presence of a magnetic field whereby only paramagnetic systems can show EPR signals, and there is no interference from diamagnetic background. The number of lines expected for vanadium species is given by the formula ($2nI + 1$) where n is the number of nuclei (one for vanadium) and I is the spin (7/2 for vanadium), and 8 lines are expected. The *g*-factor also gives information about the paramagnetic centres.

EPR spectroscopy examines the transitions between electron spin states separated by the presence of an external magnetic field. These states are separated by energy which is dependent on the *g* value of the observed species. For the theoretical free electron, $g = 2.0023$ for vanadium(IV) and this can change when the metal ion is coordinated with ligands. Values of *g* in vanadyl EPR spectra are typically less than the free electron value. The EPR spin Hamiltonian parameters ($A_{\parallel}$, $A_{\perp}$, $g_{\parallel}$, and $g_{\perp}$) also give information on the geometry of the oxidovanadium(IV) complexes by comparing them with model complexes. The spin Hamiltonian parameters can be calculated from EPR spectra using the computer program developed by Rockenbauer and Korecz [10].

#### (a) Speciation of vanadyl-L-small bioligands systems

**Figures S36 and S37** show overlaid EPR spectra of vanadyl and its interaction with the respective carrier ligands (Im2COOH and MeIm2COOH) and bioligands, and the results indicated that there are some structural changes with the complexes and are due to interaction of vanadyl with the ligands (L) and small bioligands.

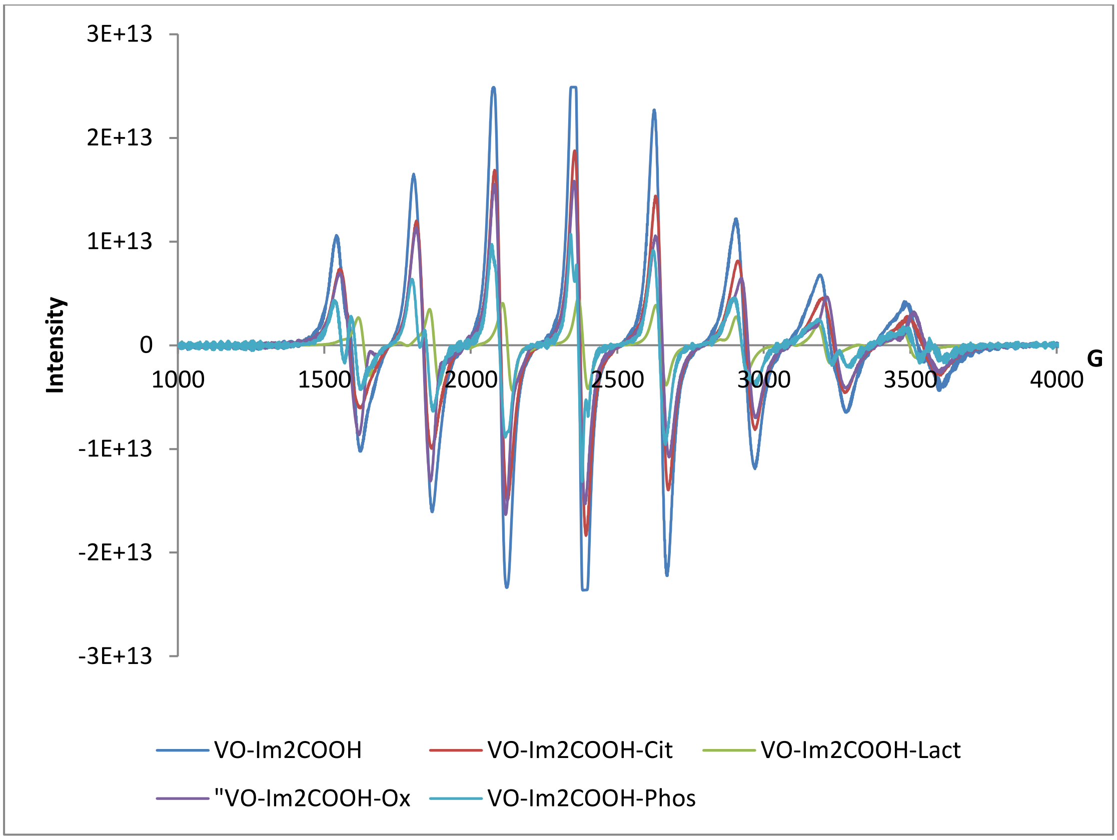


**Figure S36.** EPR spectra of VO(IV) with Im2COOH (LH) and low molecular bio-ligands of human plasma, pH = 7.4.

The spectra of VO-Im4COOH-Lact, VO-Im2COOH-Lact and VO-MeIm2COOH-Lact present the EPR parameters existing between the one presented by VO-$O_2$C and the complexes [VO(Im4COO)$_2$], [VO(Im2COO)$_2$], [VO(MeIm2COO)$_2$]. For example the hyperfine structures for the nuclear spin of vanadium with parallel $g_{\perp}$ = 1.972, 1.972 and 1.974 for VO-Im4COOH-Lact, VO-Im2COOH-Lact and VO-MeIm2COOH-Lact systems respectively, and are higher (1.936, 1.936, 1.938) than for [VOL$_2$] of (Im4COOH, Im2COOH and MeIm2COOH respectively) (**Table 3**). This confirms the presence of the species [VOLLact] and [VOL(Lact)OH] that exist at physiological pH (7.4).

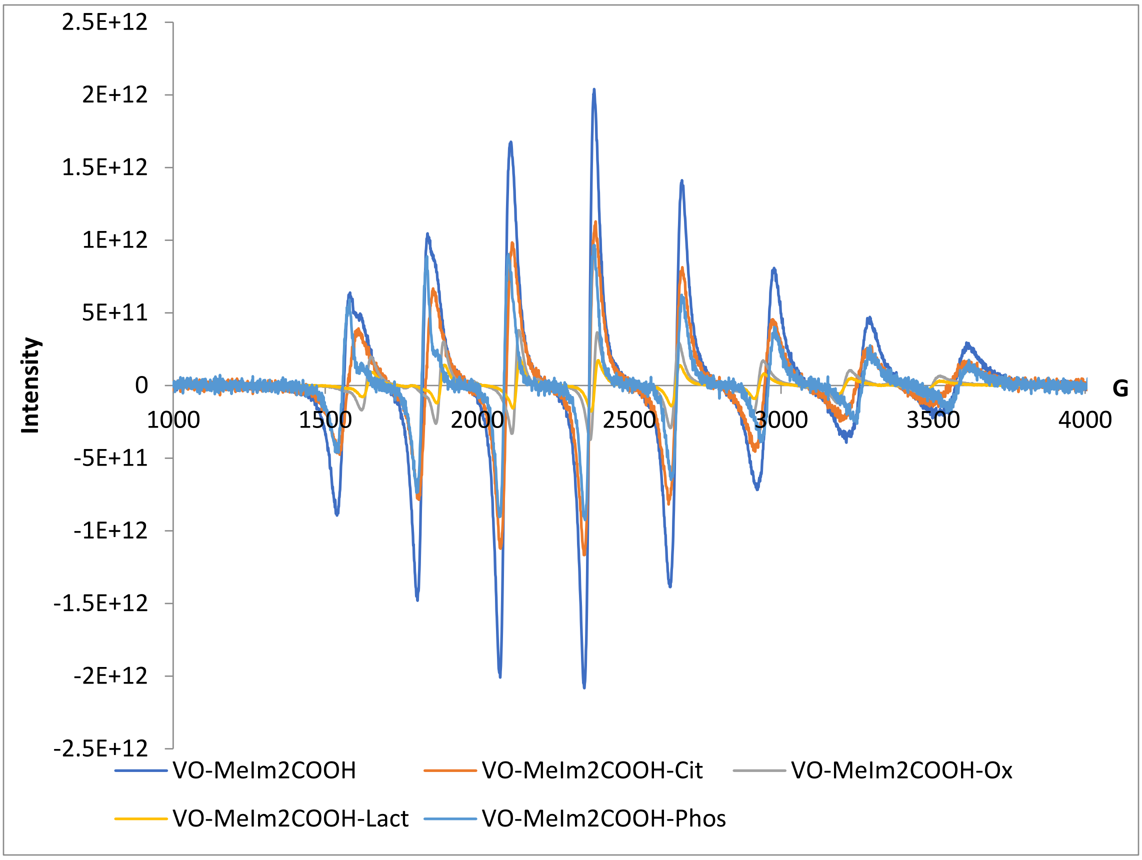


**Figure S37.** EPR spectra of VO(IV) with MeIm2COOH (LH) and small bioligands of human plasma, pH = 7.4.

**(b) Speciation of vanadyl-large bioligands systems**

**Figures S38** and **S39** represent the EPR spectra of VO(IV) with Im4COOH and MeIm2COOH with high molecular weight ligands of human plasma respectively at pH = 7.4. In general, EPR parameters, $g_{II}$ and $A_{II}$ and $g_{\perp}$ and $A_{\perp}$ for the ligand MeIm2COOH are much higher than those for unsubstituted Im4COOH and Im2COOH. For example, $g_{\perp}$ values are 1.980, 1.980 and 1.981 for Im4COOH Im2COOH and MeIm2COOH, respectively. Vanadyl complexes of serum albumin and serum transferrin, which are the major carriers of vanadium in the blood, have been examined using different techniques and the species of the form $VOL_2$, $[VO(HSA)_2]$ and $[(VO)_2(L)_2(HSA)]$ for human serum albumin, and $[VOL_2]$, $[VO(hTf)_2]$, and $[(VO)_2(L)_2(hTf)]$ for human serum transferrin have been identified. These species were confirmed by comparing the experimental g⊥ of 1.980, 1.980 and 1.981 for VO-Im4COOH-HSA, VO-Im2COOH-HSA, VO-MeIm2COOH-HAS systems with the one reported for vanadyl–albumin (1.979) [11] as well as 1.936, 1.936 and 1.938 for VO-Im4COOH, VO-Im2COOH and VO-MeIm2COOH, respectively. All the experimental parameters of transferrin systems are slightly much higher than those of albumin systems and this is probably due to its VO(IV) binding ability.

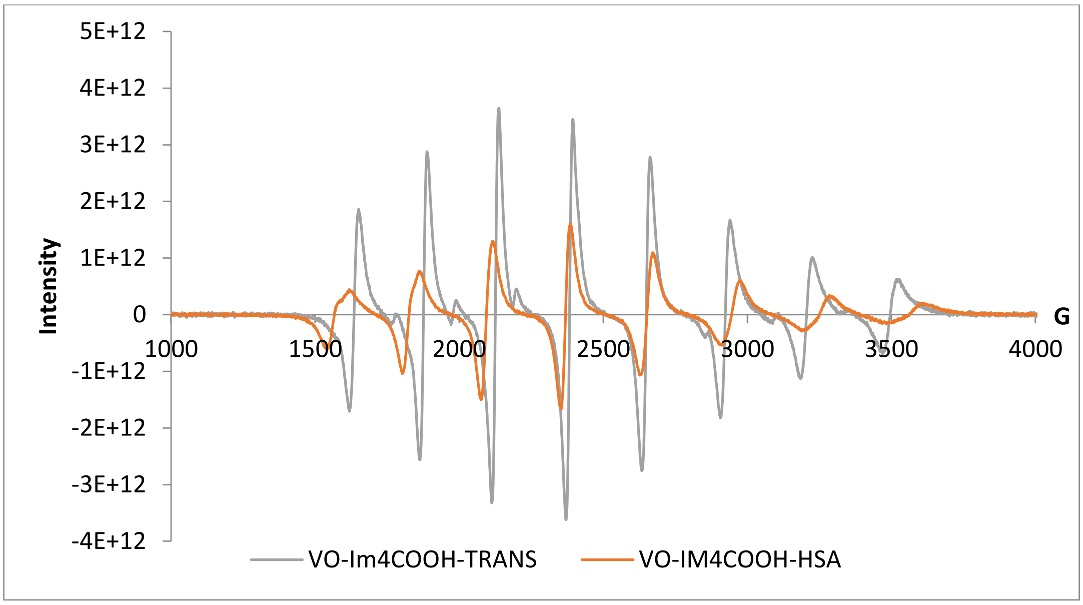


**Figure S38**. EPR spectra of VO(IV) with Im4COOH (LH) and high molecular weight ligands of human plasma, pH = 7.4.

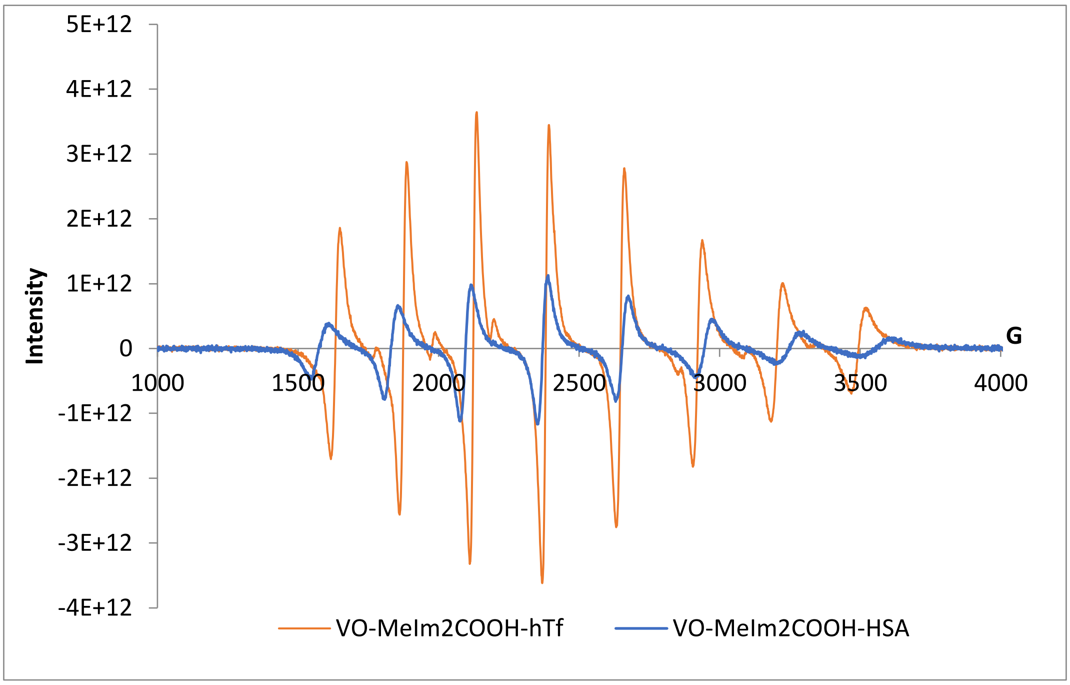


**Figure S39**. EPR spectra of VO(IV) with MeIm2COOH (LH) and high molecular weight bioligands of human plasma, pH = 7.4.

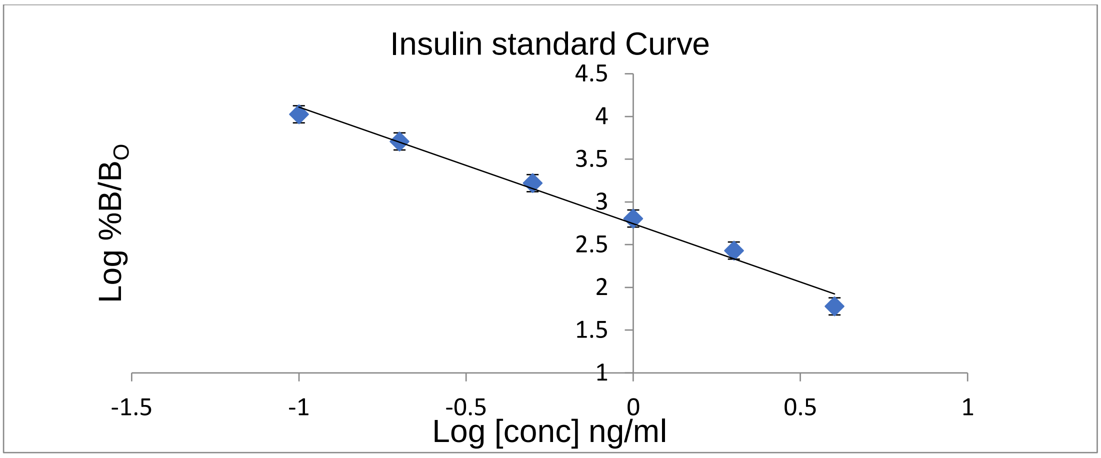


**Figure S40**. An example of an ELISA kit standard curve of rat insulin standards (0.2- 10 ng/ml) used during experimentation, error bars indicate SEM of triplicate standards, $R^2$ = 0.987, (n=3).

## References

[1] I.Z. Gundhla, V. Ugirinema, R.S. Walmsley, N.O. Mnonopi, E. Hosten, R. Betz, C.L. Frost and Zenixole R. Tshentu. pH-metric chemical speciation modeling and *in vitro* anti-diabetic studies of *bis*[(imidazolyl)carboxylato]oxovanadium(IV) complexes. *J. Inorg. Biochem.*, 145, 2015, 11–18.

[2] K. J. Oberhausen, J. F. Richardson, R. M. Buchanan, W. Pierce, Synthesis, structure and properties of a N3 tridentate bis-imidazolyl ligand with copper (II). *Polyhedron*, 8, (1989), 659.

[3] L. I. Kruse, C. Kaiser, W. E. DeWolf, J. A. Finkelstein, J. S. Frazee, E. L. Hilbert, S. T. Ross, K. E. Flaim, J. L. Sawyer, Some benzyl-substituted imidazoles, triazoles, tetrazoles, pyridinethiones, and structural relatives as multisubstrate inhibitors of dopamine. beta.-hydroxylase. 4. Structure-activity relationships at the copper binding site. *J. Med. Chem*., 33 (1990), 781.

[4] P. Kleyi, R.S. Walmsley, I. Z. Gundhla, A.T. Walmsley, T.I. Jauka, J. Dames, R. B. Walker, N. Torto, Z. R. Tshentu, Z.R. Syntheses, protonation constants and antimicrobial activity of 2-substituted N-alkylimidazole derivatives. *S. Afr. J. Chem*, 65, (2012), 231.

[5] T.T. Kiss, E. Kiss, G. Micera, D. Sanna.The formation of ternary complexes between VO(maltolate)2 and small bioligands, *Inorg. Chim. Acta*, 283, 1998, 202.

[6] P. Buglyó, E. Kiss, I. Fábián, T. Kiss, D. Sanna, E. Garribba, G. Micera. Interaction between the low molecular mass components of blood serum and the VO(IV)–DHP system (DHP = 1,2-dimethyl-3- hydroxy-4(1H)-pyridinone), *Inorg. Chim. Acta*, 34, 2000, 174.

[7] J. K. Lewis, J. Wei, G. Siuzdak.Matrix-assisted Laser Desorption/Ionization Mass Spectrometry in Peptide and Protein Analysis, In Enc. Anal. Chem R.A. Meyers (Ed.), 2000, 5880.

[8] T. Jakusch, D. Hollender, E.A. Enyedy, C.S. Gonzalez, M.M. Bayon, A.S. Medel, J.C. Pessoa, T.T. Kiss.Vanadium in Biological Action: Chemical, Pharmacological Aspects, and Metabolic Implications in Diabetes Mellitus, *J. Inorg. and Anal. Chem.,* 13, 2009, 2428.

[9] R.S. Walmsley, Z.R. Tshentu, M.A. Fernandes and C.L. Frost. Synthesis, characterization and anti-diabetic effect of *bis*[(1-R-imidazolinyl)phenolato]oxovanadium(IV) complexes. *Inorg. Chim. Acta*, 363, 2010, 2215–2221.

[10] A. Rockenbauer and L. Korecz, (1996), Automatic computer simulations of ESR spectra, *Appl. Magn. Reson*, 10, 1996, 29.

[11] C.R. Cornman, P. Edward, Y.D. Zovinka, K. M. Boyajian, R.M. Geiser-Bush, P.D. Boyle and P. Singh. Correlation of the EPR hyperfine constant with ring orientation, *Inorg. Chem.*, 34, 1995, 4213.
